# Stretching vibrational frequencies and p*K*_a_ differences in H-bond networks of protein environments

**DOI:** 10.1016/j.bpj.2023.10.012

**Published:** 2023-10-14

**Authors:** Masaki Tsujimura, Keisuke Saito, Hiroshi Ishikita

**Affiliations:** 1 Department of Advanced Interdisciplinary Studies, The University of Tokyo, Meguro-ku, Tokyo, Japan; 2 Department of Applied Chemistry, The University of Tokyo, Bunkyo-ku, Tokyo, Japan; 3 Research Center for Advanced Science and Technology, The University of Tokyo, Meguro-ku, Tokyo, Japan

## Abstract

The experimentally measured stretching vibrational frequencies of O–D [*ν*_O–D_(donor)] and C=O [*ν*_C=O_(donor)] H-bond donor groups can provide valuable information about the H-bonds in proteins. Here, using a quantum mechanical/molecular mechanical approach, the relationship between these vibrational frequencies and the difference in p*K*_a_ values between H-bond donor and acceptor groups [Δp*K*_a_(donor … acceptor)] in bacteriorhodopsin and photoactive yellow protein environments was investigated. The results show that *ν*_O–D_(donor) is correlated with Δp*K*_a_(donor … acceptor), regardless of the specific protein environment. *ν*_C=O_(donor) is also correlated with Δp*K*_a_(donor … acceptor), although the correlation is weak because the C=O bond does not have a proton. Importantly, the shifts in *ν*_O–D_(donor) and *ν*_C=O_(donor) are not caused by changes in p*K*_a_(donor) alone, but rather by changes in Δp*K*_a_(donor … acceptor). Specifically, a decrease in Δp*K*_a_(donor … acceptor) can lead to proton release from the H-bond donor group toward the acceptor group, resulting in shifts in the vibrational frequencies of the protein environment. These findings suggest that changes in the stretching vibrational frequencies, in particular *ν*_O–D_(donor), can be used to monitor proton transfer in protein environments.

## Significance

The stretching vibrational frequencies of O–D [*ν*_O–D_(donor)] and C=O [*ν*_C=O_(donor)] bonds in proteins are valuable tools for understanding H-bond characteristics in proteins. This study used a quantum mechanical/molecular mechanical approach to investigate the relationship between these vibrational frequencies and the difference in p*K*_a_ between H-bond donor and acceptor groups [Δp*K*_a_(donor … acceptor)] in bacteriorhodopsin and photoactive yellow protein environments. The results reveal that *ν*_O–D_(donor) is strongly correlated with Δp*K*_a_(donor … acceptor), providing a means of measuring p*K*_a_ values that are otherwise often difficult to directly obtain. This finding has significant implications for understanding biological processes such as proton transport, protein stability, and enzyme catalysis.

## Introduction

**Figure 1:**
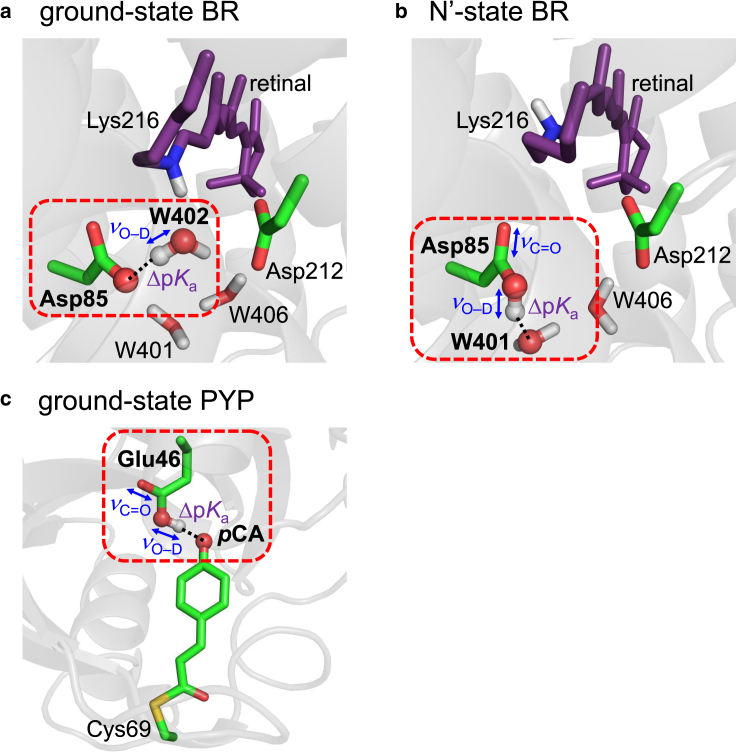
H-bonds investigated in this study. (*a*) [DO_W402_–D … ^–^OOC-Asp85] in the ground-state BR (PDB: 5ZIM (18)). W402 serves as an H-bond donor, whereas Asp85 serves as an H-bond acceptor. (*b*) [Asp85-COOD … O_W401_D_2_] in the N′-state BR (PDB: 1P8U (19)). Asp85 serves as an H-bond donor, whereas W401 serves as an H-bond acceptor. (*c*) [Glu46-COOD … ^–^O-*p*CA] in the ground-state PYP (PDB: 1OT9 (20)). Glu46 serves as an H-bond donor, whereas *p*CA serves as an H-bond acceptor. Dotted lines indicate H-bonds. To see this figure in color, go online.

Bacteriorhodopsin (BR) is a light-driven proton pump membrane protein (1,2). The chromophore is all-*trans* retinal covalently attached to Lys216 via the protonated Schiff base (Fig. 1 *a*) (2). The identification of water molecules at the Schiff base moiety was initially based on findings from Fourier transform infrared (FTIR) spectroscopy (3,4). These findings were subsequently confirmed in the X-ray crystal structure, revealing the presence of conserved water molecules identified as W402, W401, and W406 in the ground-state BR (2). In the ground state, the protonated Schiff base forms a pentagonal H-bond network with Asp85, Asp212, W402, W401, and W406 (Fig. 1 *a*) (2). Specifically, W402 accepts an H-bond from the Schiff base and also donates H-bonds to Asp85 and Asp212. W401 and W406 donate H-bonds to Asp85 and Asp212, respectively. The photocycle is initiated by the all-*trans* to 13-*cis* photoisomerization of the retinal chromophore (5). The proton released from the retinal Schiff base is transferred toward the extracellular side (5,6,7). Proton transfer can be investigated using FTIR spectroscopy and measuring the vibrational frequencies of H-bonds along the proton transfer pathway, because H-bond formation decreases the O–D stretching vibrational frequency for D_2_O, *ν*_O–D_(D_2_O) (= ∼2700 cm^−1^ in the gas phase (8)) to ∼2000–2600 cm^−1^ (9,10). A common feature of proton-pumping microbial rhodopsins is the presence of a water molecule with *ν*_O–D_(D_2_O) < 2400 cm^−1^ in the ground state (11,12). In particular, the lowest *ν*_O–D_(D_2_O) was observed at ∼2200 cm^−1^ in the ground-state BR using FTIR spectroscopy (9,10). The water molecule (W402 (2)), which donates H-bonds to Asp85 and Asp212 near the Schiff base, has the lowest *ν*_O–D_(D_2_O) (Fig. 1 *a*) (9,10,13,14). In the two H-bonds, the W402 … Asp85 H-bond exhibits the lowest *ν*_O–D_(D_2_O) in the ground state BR, which plays a key role in the proton-pumping activity (9,10,13,14).

Before exploring the details of H-bonds, it is crucial to clarify the difference between “H^+^ donor/acceptor” and “H-bond donor/acceptor.” During proton transfer (i.e., transient state), the proton migrates from the “(more acidic) H^+^ donor” moiety toward the “(more basic) H^+^ acceptor” moiety. However, when considering H-bonds (i.e., resulting equilibrium state), the proton is predominantly localized at the “(more basic) H-bond donor” moiety rather than the “(more acidic) H-bond acceptor” moiety (15,16). Thus, in standard H-bonds, p*K*_a_[(H-bond) donor] is higher than p*K*_a_[(H-bond) acceptor], resulting in a positive p*K*_a_ difference between the H-bond donor and acceptor moieties [Δp*K*_a_(donor ... acceptor) = p*K*_a_(donor) – p*K*_a_(acceptor) > 0]. Notably, the concept of low-barrier hydrogen bonds arises when Δp*K*_a_(donor ... acceptor) = 0 (17). In this context, a decrease in *ν*_O–D_(D_2_O) corresponds to a decrease in Δp*K*_a_(donor … acceptor), leading to the migration of the proton toward the H-bond acceptor moiety (14,16). This explanation provides a comprehensive framework for understanding why low *ν*_O–D_(D_2_O) values are indicative of proton-pumping activity.

In BR, the H-bond network that proceeds from the intracellular side via the retinal Schiff base toward the extracellular side involves not only water molecules but also acidic residues, e.g., Asp85, Asp96, Glu194, and Glu204, all of which form proton transfer pathways (5,6,7). The C=O stretching vibrational frequency (*ν*_C=O_) for protonated carboxylate (1710–1770 cm^−1^) is significantly higher than that for deprotonated carboxylate (∼1400 and ∼1570 cm^−1^ for the symmetric and asymmetric stretching vibrations, respectively) (21). Thus, from the remarkable shift in *ν*_C=O_, a change in the protonation state can be unambiguously detected, although carboxylate is more likely deprotonated due to the low p*K*_a_ value.

Notably, protonated carboxylate is reported in an intermediate state of BR. The release of the proton from protonated Asp96 toward the deprotonated retinal Schiff base occurs in the M to N transition (5,7). Accordingly, the adjacent counterion, Asp85, is fully protonated in the M state with p*K*_a_(Asp85) = 13 (22) and *ν*_C=O_(Asp85) = 1761 cm^−1^ (23) and partially protonated in the N state with p*K*_a_(Asp85) = 7 (22) and *ν*_C=O_(Asp85) = 1756 cm^−1^ (Fig. 1 *b*) (23). The observed shift in *ν*_C=O_ is clearly distinct with respect to the spectral resolution, which is approximately in the range of 2–4 cm^−1^, as reported in (23,24,25). p*K*_a_(Asp85) = 7 in the N state was estimated based on the N′-state structure of the V49A mutant BR (22). The H-bond partner of Asp85 is a water molecule (W401 (19)) (Fig. 1 *b*). Because the Schiff base is closer to Asp85 (5.5 Å (19)) than to W401 (7.7 Å (19)), protonation of the Schiff base decreases p*K*_a_(Asp85) more significantly than p*K*_a_(W401). Thus, the decrease in p*K*_a_(Asp85) in the M to N transition corresponds to the decrease in the p*K*_a_ difference between Asp85 and the H-bond acceptor water molecule [Δp*K*_a_(Asp85 … O_W401_H_2_)]. The M to N state transition is the only case among the photocycle intermediates in BR, where both *ν*_C=O_ and p*K*_a_ are reported.

A water-soluble photosensor protein, photoactive yellow protein (PYP) (26,27,28), is also one of the limited cases in which both p*K*_a_ and *ν*_C=O_ have been reported for some intermediate states during the photocycle. The *trans* to *cis* photoisomerization of *p*-coumaric acid (*p*CA) leads to the ground (pG) to pR state transition (29,30,31,32). In the pG state, protonated Glu46 donates an H-bond to *p*CA (Fig. 1 *c*) (33,34,35,36,37,38,39). In contrast, Glu46 forms a low-barrier H-bond with *p*CA in the pR state, which leads to proton transfer from Glu46 toward *p*CA (16,35,40). The p*K*_a_ difference between Glu46 and the H-bond acceptor *p*CA [Δp*K*_a_(Glu46 … *p*CA)] decreases from ∼3 to ∼0 in the pG to pR state transition (33,35), as *ν*_C=O_(Glu46) decreases from 1740 to 1732 cm^−1^ (24).

Both Asp85 in the M to N transition of BR and Glu46 in the pG to pR transition of PYP commonly show that *ν*_C=O_(donor) decreases as Δp*K*_a_(donor … acceptor) decreases. This tendency is consistent with that observed for saturated carboxylates in water (i.e., in the absence of the protein environment) (22). The decrease in Δp*K*_a_(donor … acceptor) corresponds to migration of the carboxylate proton toward the H-bond acceptor group due to formation of a low-barrier H-bond (16). The decrease in *ν*_C=O_(donor) also corresponds to the migration of the carboxylate proton toward the H-bond acceptor group (24,35). Based on these observations, Δp*K*_a_(donor … acceptor) may be estimated from experimentally measured *ν*_C=O_(donor) in the protein environment as long as the carboxylate exists in the same protein environment. This may also hold true for Δp*K*_a_(donor … acceptor) and experimentally measured *ν*_O–D_(donor) in the protein environment. However, to the best of our knowledge, successful examples of estimating Δp*K*_a_(donor … acceptor) from experimentally measured *ν*_O–D_(donor)/*ν*_C=O_(donor) in protein environments have not been reported. Thus, it remains unclear whether these vibrational frequencies are directly linked with the p*K*_a_ values of the H-bonds.

Here, we investigated how *ν*_O–D_(donor) and *ν*_C=O_(donor) are linked with the p*K*_a_ difference between the H-bond donor and acceptor moieties in the protein environment. Since the H-bond pairs, in which both Δp*K*_a_(donor … acceptor) and *ν*_O–D_(donor)/*ν*_C=O_(donor) are reported, are limited (e.g., Glu46 … *p*CA in the ground state of PYP (24,33,35)), the influences of the residues on Δp*K*_a_(donor … acceptor) and *ν*_O–D_(donor)/*ν*_C=O_(donor) values for the focused H-bond pairs are also calculated in the protein environments, using a quantum mechanical/molecular mechanical (QM/MM) approach.

## Methods

### Electrostatic calculations

The geometries of 15 H-bond pairs were optimized using the restricted density functional theory method with the B3LYP functional and the 6-31G^∗^ basis set, using the Jaguar program code (41). The H-bonds in the protein environments are investigated using the x-ray crystal structures of the ground-state BR (PDB: 5ZIM (18)), the N′-state BR (V49A mutant) (PDB: 1P8U (19)), and the ground-state PYP (PDB: 1OT9 (20)). The H atom positions were optimized using the CHARMM program code (42). Atomic charges and force field parameters were obtained from the CHARMM22 parameter set (43) for amino acids and water and CHARMM-GUI (44) for the retinal Schiff base. Atomic charges of *p*CA were determined by fitting the electrostatic potential using the restrained electrostatic potential procedure (45) as empirically recommended for the subsequent step (solving the linear Poisson-Boltzmann equation) (46,47). To ensure internal consistency, the restrained electrostatic potential charges were also used for constructing the initial geometry of *p*CA in this study. It is worth mentioning that *p*CA was included in the QM region and the geometry was QM/MM-optimized when calculating *ν*_O–D_(donor), *ν*_C=O_(donor), and Δp*K*_a_(donor … acceptor) (see below).

Using the resulting atomic coordinates (Data S1), the protonation pattern in the ground- and N′-state BR was determined based on the electrostatic continuum model, solving the linear Poisson-Boltzmann equation with the MEAD program (48). The experimentally measured p*K*_a_ values employed as references were 12.0 for Arg, 4.0 for Asp, 9.5 for Cys, 4.4 for Glu, 10.4 for Lys, 9.6 for Tyr, (49), and 7.0 and 6.6 for the N_ε_ and N_δ_ atoms of His, respectively (50,51,52). The dielectric constants were set to 4 for the protein interior and 80 for water. All water molecules were considered implicitly. All computations were performed at 300 K, pH 7.0, and with an ionic strength of 100 mM. The linear Poisson-Boltzmann equation was solved using a three-step grid-focusing procedure at resolutions of 2.5, 1.0, and 0.3 Å. The ensemble of the protonation pattern was sampled by the Monte Carlo method with the Karlsberg program (53). The Monte Carlo sampling yielded the protonation probabilities of each residue. Residues whose calculated protonation states are nonstandard (i.e., protonated acidic and deprotonated basic groups) are listed in Tables S1 and S2. All titratable groups in the ground-state PYP were kept in the standard protonation states except for the Glu46 … *p*CA pair, as done in previous studies (40). For the Glu46 … *p*CA pair, the Glu46-COOH … ^−^O-*p*CA protonation pattern was adopted (40).

To calculate the p*K*_a_ values of the H-bond donor and acceptor under investigation and compare with the Δp*K*_a_(donor … acceptor) values calculated using a QM/MM approach (Tables S3–S5), the difference in the p*K*_a_ value in the protein relative to the reference system was calculated and then added to the known reference p*K*_a_ value. All other titratable sites were fully equilibrated to the protonation state of the target site during titration. A bias potential was applied to obtain an equal amount of both protonation states ([protonated] = [deprotonated]), yielding the p*K*_a_ value as the resulting bias potential.

### QM/MM calculations

#### Geometry optimization

The geometry was optimized using a QM/MM approach. The restricted density functional theory method was employed with the B3LYP functional and the 6-31G^∗^ basis set, using the QSite program code (54). The QM region was first defined as follows (Fig. S1): the retinal, the side chains of Lys216 (Schiff base), Tyr57, Arg82, Asp85, Trp86, Thr89, Tyr185, and Asp212, and the three water molecules at the Schiff base moiety (W402, 401, and 406) for the ground-state BR; the side chains of Tyr57, Asp85, and Asp212 and the adjacent water molecules (W401, 406, and 407 (19)) for the N′-state BR; and the entireties of *p*CA, Glu46, Thr50, and Cys69 and the side chain of Tyr42 for the ground-state PYP. All atomic coordinates were fully relaxed in the QM region. In the MM region, the positions of H atoms were optimized using the OPLS2005 force field (55), while the positions of the heavy atoms were fixed. The protonation pattern of titratable residues was implemented in the atomic partial charges of the corresponding MM region.

#### Analysis of electrostatic contributions to stretching vibrational frequency and ΔpK_a_ values

The electrostatic contribution of a focusing residue in the MM region to *ν*_O–D_(donor), *ν*_C=O_(donor), and Δp*K*_a_(donor … acceptor) in the QM region was evaluated through the following steps: 1) removing the atomic charges of the focusing side chain in the MM region, thereby eliminating its electrostatic influence (i.e., absence of the residue’s electrostatic contribution), 2) reoptimizing the geometry using a QM/MM approach, and 3) calculating the resulting shifts in *ν*_O–D_(donor), *ν*_C=O_(donor), and Δp*K*_a_(donor … acceptor). To facilitate this analysis, the QM region was redefined to solely include the H-bond donor and acceptor under investigation: W402 and the side chain of Asp85 for the ground-state BR; the side chain of Asp85 and W401 for the N′-state BR; and the entirety of *p*CA and the side chains of Glu46 and Cys69 for the ground-state PYP (Fig. S1). All atomic coordinates were fully relaxed in the QM region, while all atomic coordinates were fixed in the MM region. This approach was taken to avoid unrealistic displacements of superficial H atoms in the MM region caused by the removal of atomic charges, since such displacements might potentially introduce artifacts to the resulting shifts in *ν*_O–D_(donor), *ν*_C=O_(donor), and Δp*K*_a_(donor … acceptor).

To obtain the potential energy profiles for proton transfer, the quantum-chemically optimized geometry was used as the initial geometry. The H atom under investigation was moved from the H-bond donor atom (D) toward the acceptor atom (A) by 0.05 Å, after which the geometry was optimized by constraining the H–A distance, and the energy was calculated. These procedures were repeated until the H atom reached the A atom. The H-bond acceptor moiety was defined at the point where the H–A distance was 1.00 Å. All atomic coordinates were fully relaxed in the QM region, whereas all atomic coordinates were fixed in the MM region. The energy difference between the [D–H … A] and [D … H–A] states [Δ*E*(donor … acceptor)] was converted into Δp*K*_a_(donor … acceptor) using Eq. 1 (see below).

Vibrational frequencies were calculated using the same level of theory as the geometry optimizations based on the quantum-chemically optimized structures. The calculated frequencies were scaled by using a standard factor of 0.9614 for the B3LYP functional (56). *ν*_O–D_ was calculated by deuterating only the hydrogen atom of the H-bond donor moiety and avoiding mode coupling. *ν*_C=O_ was calculated for the COOH group (not for the deuterated COOD group).

## Results and discussion

### Estimation of **Δ**p*K*_a_(donor … acceptor) in H-bonds

**Figure 2:**
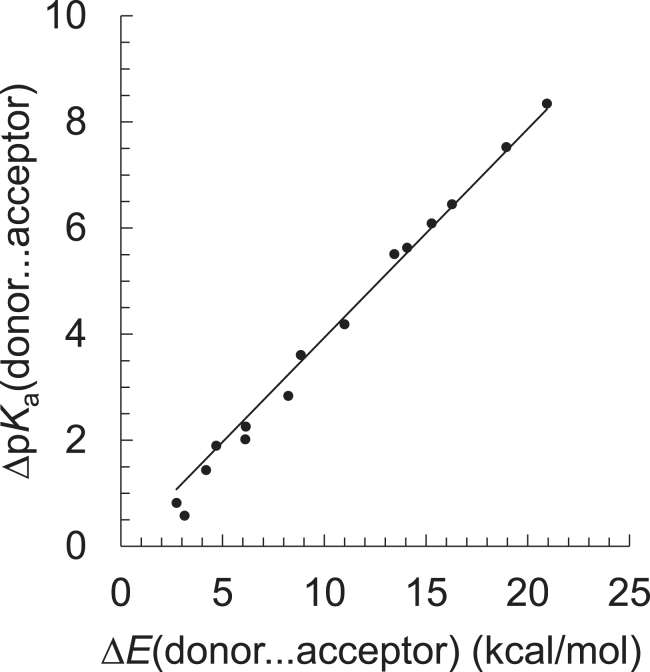
Δp*K*_a_(donor … acceptor) and Δ*E*(donor … acceptor) obtained from 15 H-bond pairs. See Table 1 for the H-bond donor/acceptor groups.

To calculate Δp*K*_a_(donor … acceptor) in the protein environments using a QM/MM approach, the potential-energy profiles for all 15 possible H-bond pairs from 6 alcohol/phenol molecules (Table 1) are analyzed. In all H-bond pairs, the H-bond donor is –OH. The energy difference between the [O_donor_–H … ^−^O_acceptor_] and [O_donor_^−^ … H–O_acceptor_] states [Δ*E*(donor … acceptor)] obtained from the potential-energy profile is highly correlated with the experimentally measured Δp*K*_a_(donor … acceptor) at 298 K for the H-bond pair, as suggested previously (15,16,17,57) (Fig. 2; Table 1). The correlation is best described by the following equation (coefficient of determination *R*^2^ = 0.99):

(1) $$\Delta pK_{a}\left(\text{donor }...\;\text{acceptor}\right)=0.39\;\left[\text{mol}/\text{kcal}\right]\;\Delta E\left(\text{donor }...\;\text{acceptor}\right)$$

**Table 1:** Isolated 15 H-bond pairs

H-bond donor		H-bond acceptor		
	p*K*_a_^a^		p*K*_a_^a^	Δp*K*_a_
methanol	15.5	2-propyn-1-ol	13.6	1.9
		phenol	9.99	5.5
		4-chlorophenol	9.41	6.1
		4-cyanophenol	7.97	7.5
		4-nitrophenol	7.15	8.4
2-propyn-1-ol	13.6	phenol	9.99	3.6
		4-chlorophenol	9.41	4.2
		4-cyanophenol	7.97	5.6
		4-nitrophenol	7.15	6.5
phenol	9.99	4-chlorophenol	9.41	0.58
		4-cyanophenol	7.97	2.0
		4-nitrophenol	7.15	2.8
4-chlorophenol	9.41	4-cyanophenol	7.97	1.4
		4-nitrophenol	7.15	2.3
4-cyanophenol	7.97	4-nitrophenol	7.15	0.82

a Ref. (58).

The accuracy of the Δp*K*_a_ predictions is demonstrated in Fig. 2, showing a deviation of only 0.26 p*K*_a_ units. This approach has also been successfully employed and validated in a previous study (57). p*K*_a_ is linked to the reaction Gibbs free energy for deprotonation with a factor of 1/(*RT* ln10) (= 0.73 mol/kcal at 298 K). As Δ*E*(donor … acceptor) in Eq. 1 was calculated in vacuum, the difference between the factor of 0.73 in 1/(*RT* ln10) and the factor of 0.39 in Eq. 1 is primarily attributed to the insufficient consideration of the solvent reorganization in the surrounding environment.

In the ground-state PYP, Glu46 donates an H-bond to *p*CA, forming a standard H-bond (33,34,35,36,37,38,39). p*K*_a_(Glu46) = 8.6 and p*K*_a_(*p*CA) = 5.4 (i.e., Δp*K*_a_(Glu46 … *p*CA) = 3.2) are reported for the ground-state PYP (33). In the present study, Δ*E*(Glu46 … *p*CA) = 10.2 kcal/mol deduced from the potential-energy profile for the Glu46 … *p*CA H-bond leads to Δp*K*_a_(Glu46 … *p*CA) = 4.0 using Eq. 1, which is comparable with the reported value. These observations validate the reliability and consistency of the present approach. Below, Δp*K*_a_(donor … acceptor) is calculated by analyzing the potential-energy profile for the H-bond and using Eq. 1.

### ***ν***_O–D_(donor) and **Δ**p*K*_a_(donor … acceptor) in H-bonds

To understand the relationship between *ν*_O–D_(donor) and Δp*K*_a_(donor … acceptor), the influence of the residue on *ν*_O–D_(donor) and Δp*K*_a_(donor … acceptor) is analyzed using the following three H-bonds in the protein crystal structures.

**Figure 3:**
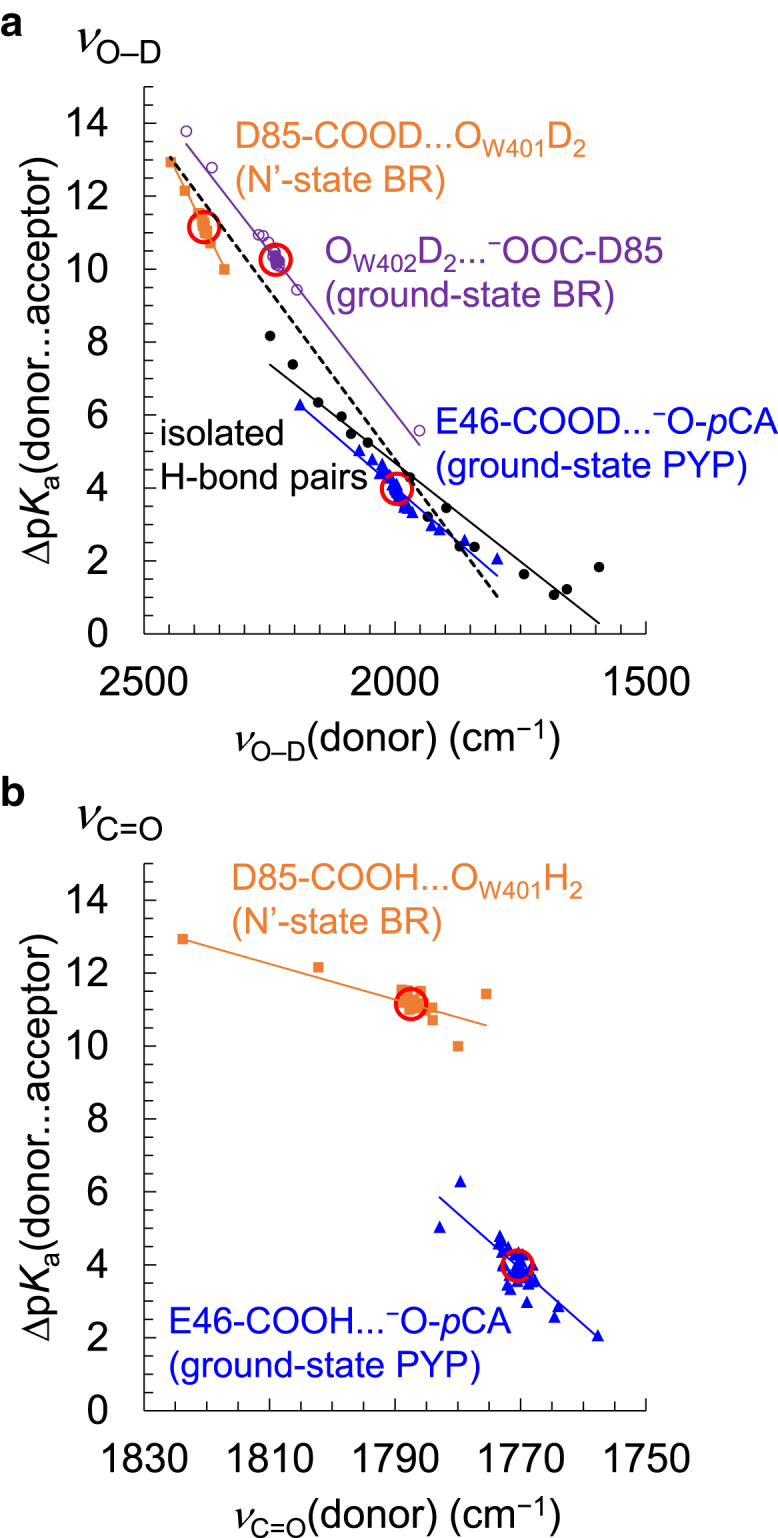
*ν*_O–D_(donor)/*ν*_C=O_(donor) and Δp*K*_a_ in the protein environments obtained from the contribution of each residue. (*a*) *ν*_O–D_(donor) and Δp*K*_a_(donor … acceptor) in the ground-state BR (*purple open circles*, *R*^2^ = 0.99), the N′-state BR (*orange squares*, *R*^2^ = 0.97), the ground-state PYP (*blue triangles*, *R*^2^ = 0.93), and isolated 15 H-bond pairs listed in Table 1 (*black closed circles*, *R*^2^ = 0.93). The dashed black line indicates the fitting line for all data points of the ground-state BR, the N′-state BR, and the ground-state PYP (*R*^2^ = 0.90). (*b*) *ν*_C=O_(donor) and Δp*K*_a_(donor … acceptor) in the N′-state BR (*orange squares*, *R*^2^ = 0.66) and the ground-state PYP (*blue triangles*, *R*^2^ = 0.70). The values in the original (not charge-depleted) structures are red circled. See Figs. S2–S4 for the details of each data point. To see this figure in color, go online.

**Figure 4:**
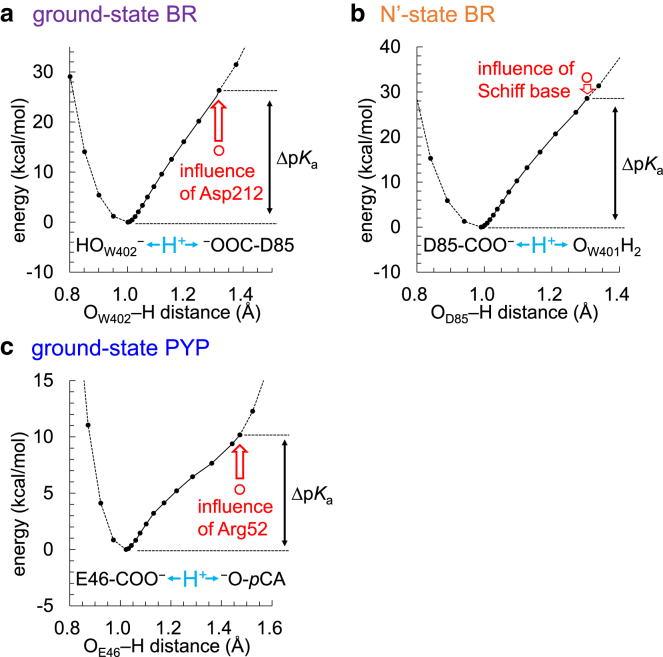
Potential energy profiles for the H-bonds. (*a*) Ground-state BR. (*b*) N′-state BR. (*c*) Ground-state PYP. Red open circles indicate the energy calculated in the absence of the electrostatic influence of the representative group (Asp212 in the ground-state BR, retinal Schiff base in the N′-state BR, and Arg52 in the ground-state PYP). Red arrows indicate the electrostatic contribution of the representative group to Δp*K*_a_(donor … acceptor). To see this figure in color, go online.
2) In contrast, protonated Asp85 donates an H-bond to W401 in the N′ state. Thus, *ν*_O–D_(Asp85) and Δp*K*_a_(Asp85-COOD … O_W401_D_2_) are calculated for the H-bond between protonated Asp85 and W401 in the N′-state structure (Fig. 3 *a*). In addition, the contributions of all BR residues (226 residues, excluding Asp85) to *ν*_O–D_(Asp85) and Δp*K*_a_(Asp85-COOD … O_W401_D_2_) are also calculated (Fig. 4 *b*).
3) In the ground-state PYP, protonated Glu46 donates an H-bond to deprotonated *p*CA. Thus, *ν*_O–D_(Glu46) and Δp*K*_a_(Glu46-COOD … ^–^O-*p*CA) are calculated for the H-bond between protonated Glu46 and deprotonated *p*CA in the ground-state PYP (Fig. 3 *a*). In addition, the contributions of all PYP residues (124 residues, excluding Glu46) to *ν*_O–D_(Glu46) and Δp*K*_a_(Glu46-COOD … ^–^O-*p*CA) are also calculated (Fig. 4 *c*).

1) In the ground-state BR, ionized Asp85 accepts an H-bond from the adjacent water molecule (W402). Thus, *ν*_O–D_(W402) and Δp*K*_a_(DO_W402_–D … ^–^OOC-Asp85) are calculated for the H-bond between deprotonated Asp85 and W402 in the ground-state structure (Fig. 3 *a*). In addition, the contributions of all BR residues (227 residues, excluding Asp85) to *ν*_O–D_(W402) and Δp*K*_a_(DO_W402_–D … ^–^OOC-Asp85) are also calculated (Fig. 4 *a*).

Remarkably, *ν*_O–D_(donor) is correlated with Δp*K*_a_(donor … acceptor) among the three protein crystal structures, using the following equation (the *dashed black line* in Fig. 3 *a*):

(2) $$\Delta pK_{a}\left(\text{donor }...\;\text{acceptor}\right)=0.019\;\left[\text{cm}\right]\;v_{O-D}\left(\text{donor}\right)\;–\;32$$

Eq. 2 can also reproduce *ν*_O–D_(donor) and Δp*K*_a_(donor … acceptor) for the 15 H-bond pairs listed in Table 1, even in the absence of the protein environment (Fig. 3 *a*). These results suggest that Δp*K*_a_(donor … acceptor) is predominantly determined by *ν*_O–D_(donor) irrespective of the presence (BR and PYP) and absence of the protein environment.

**Figure 5:**
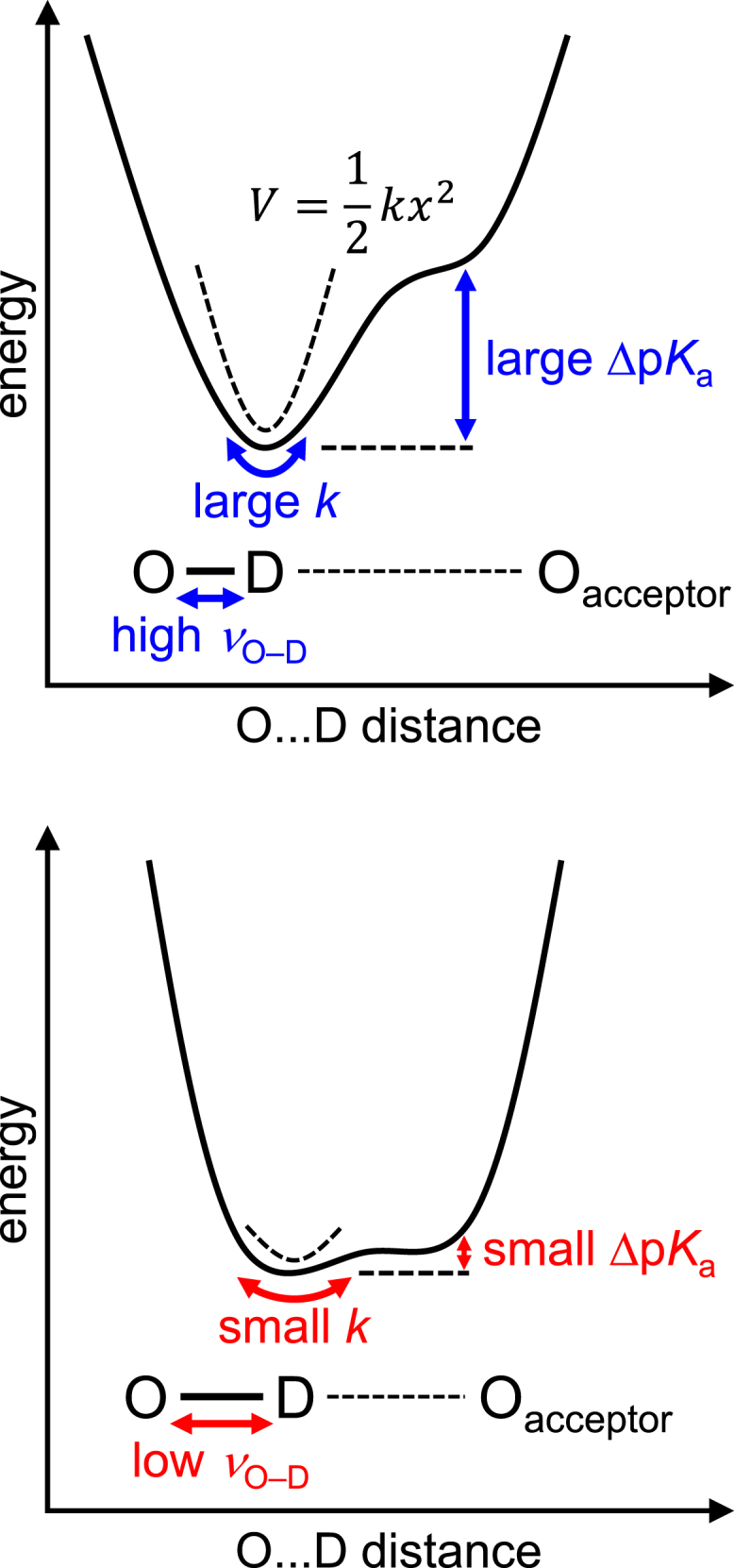
Relationship between *ν*_O–D_(donor) and Δp*K*_a_(donor … acceptor). The dotted curve indicates the fitting of the H-bond potential energy curve using a harmonic oscillator potential, *V* = *kx*^2^/2, where *k* is the spring constant and *x* is the O–D bond distance. To see this figure in color, go online.

The curvature at the H-bond donor moiety in the H-bond potential energy curve represents the spring constant *k* of the O–D harmonic oscillator (Fig. 5). The harmonic oscillator frequency, *ν*_O–D_, is proportional to the square-root of the spring constant (*ν*_O–D_∝*k*^1/2^). As Δp*K*_a_ decreases, *k* also decreases, thereby leading to a decrease in *ν*_O–D_ (Fig. 5). Consequently, there exists a positive correlation between *ν*_O–D_ and Δp*K*_a_.

**Figure 6:**
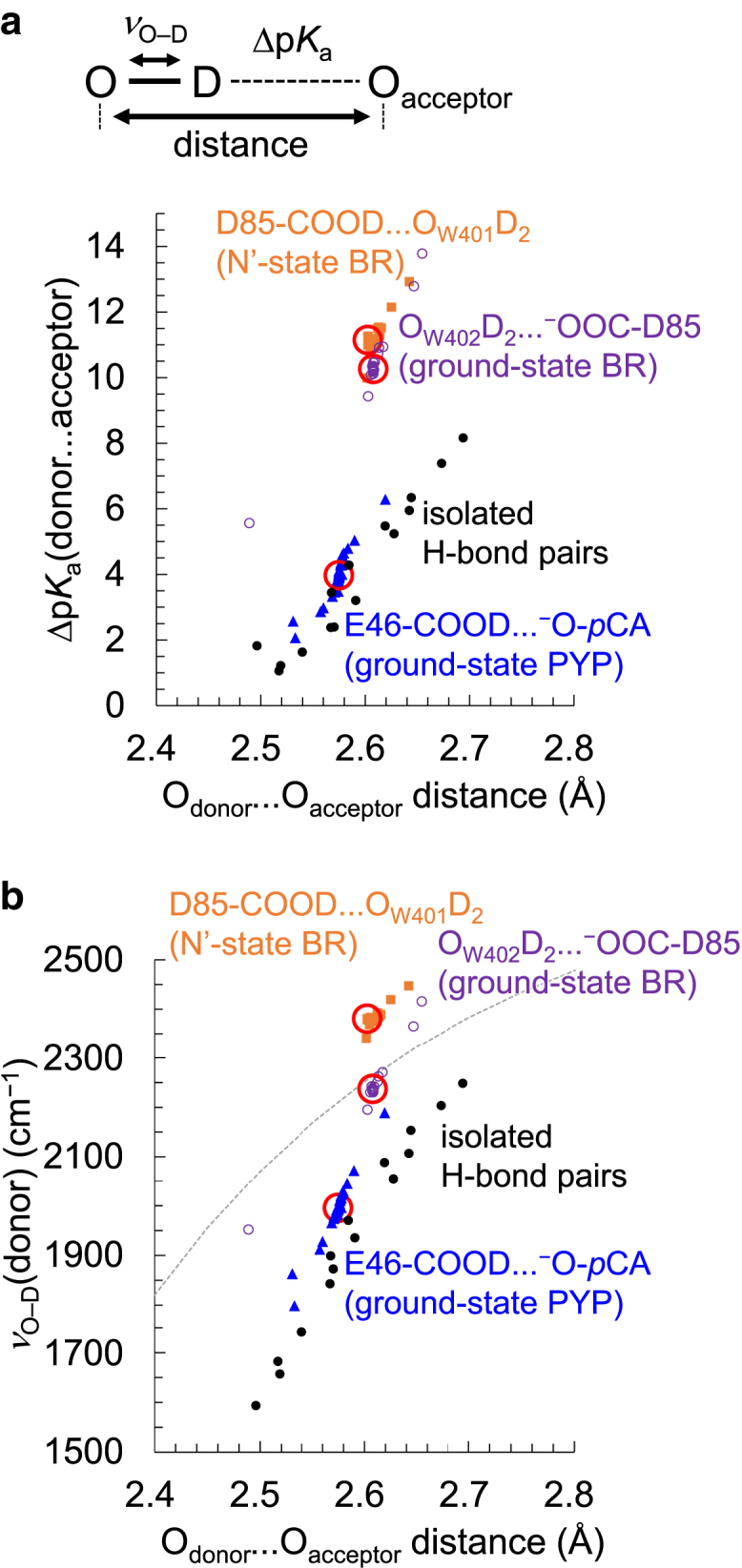
H-bond characteristics and the donor and acceptor (O_donor_ … O_acceptor_) distances. (*a*) Δp*K*_a_(donor … acceptor) and O_donor_ … O_acceptor_ distances: the ground-state BR (*purple open circles*), the N′-state BR (*orange squares*), the ground-state PYP (*blue triangles*), and isolated 15 H-bond pairs listed in Table 1 (*black closed circles*). (*b*) *ν*_O–D_(donor) and O_donor_ … O_acceptor_ distances. The gray dotted curve indicates the correlation deduced from solid hydrates (59). The values in the original (not charge-depleted) structures are red circled. To see this figure in color, go online.

Δp*K*_a_(donor … acceptor) is less correlated with the H-bond donor … acceptor distance than *ν*_O–D_(donor) (Fig. 6 *a*). This is consistent with the view that the characteristics of H-bonds, including low-barrier H-bonds (i.e., Δp*K*_a_(donor … acceptor) ≈ 0), cannot be judged by the distance but by the shape of the potential-energy profile for the H-bonds (17).

The H-bond distance is often correlated with *ν*_O–D_(donor). The correlation between H-bond distances and *ν*_O–D_(donor) observed for solid hydrates (59) is also observed for the ground-state BR (14). The present result demonstrates that *ν*_O–D_(donor) is more highly correlated with Δp*K*_a_(donor … acceptor) than with H-bond distances (Figs. 3 *a* and 6 *b*).

### ***ν***_C=O_(donor) and **Δ**p*K*_a_(donor … acceptor) in H-bonds

*ν*_C=O_ for protonated carboxylate (1710–1770 cm^−1^) is specifically high with respect to deprotonated carboxylate (∼1400 and ∼1570 cm^−1^) (21). The influence of the residue on *ν*_C=O_(donor) and Δp*K*_a_(donor … acceptor) is analyzed for the H-bond between protonated Asp85 and W401 in the N′-state BR and the H-bond between protonated Glu46 and deprotonated *p*CA in the ground-state PYP. *ν*_C=O_(Asp85) is weakly associated with Δp*K*_a_(Asp85-COOH … O_W401_H_2_) in the N′ state structure of BR (Fig. 3 *b*). *ν*_C=O_(Glu46) is also weakly associated with Δp*K*_a_(Glu46-COOH … ^–^O-*p*CA) in the ground-state structure of PYP (Fig. 3 *b*).

**Figure 7:**
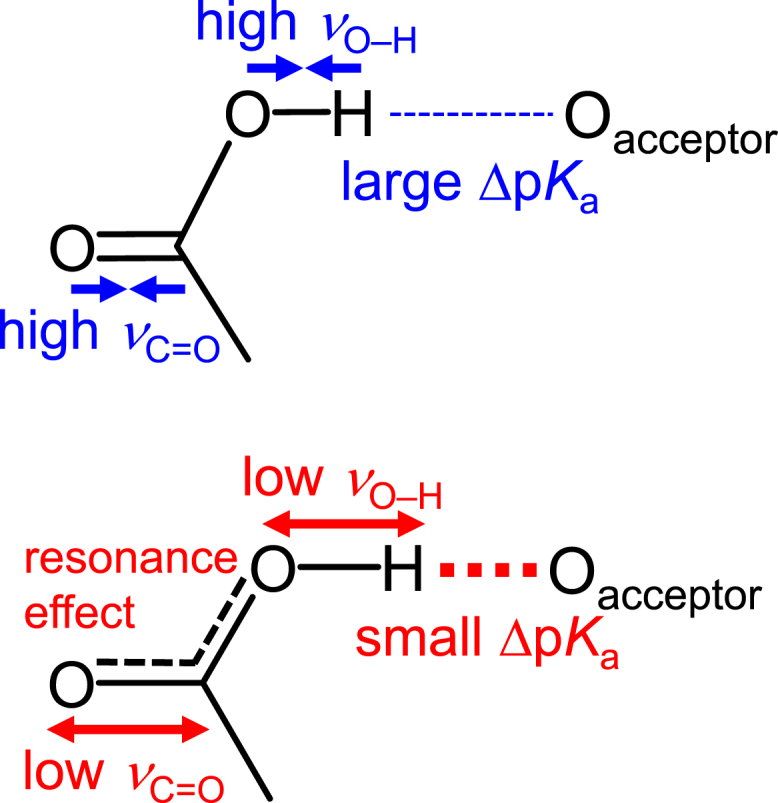
Relationship among *ν*_C=O_(donor), *ν*_O–__H_(donor), and Δp*K*_a_(donor … acceptor). To see this figure in color, go online.

The small Δp*K*_a_(donor … acceptor) leads to a decrease in *ν*_O–H_(donor) (i.e., the weakened O–H bond strength) (Fig. 3 *a*). Consequently, the resonance effect between the C=O and C–O characters is pronounced, causing a decrease in the double-bond character of the C=O bond, resulting in a decrease in *ν*_C=O_(donor) (Figs. 7 and S5 *a*). Indeed, a decrease in *ν*_O–D_(donor) (i.e., an elongated O–D distance) results in a shorter C–O distance (i.e., pronounced double-bond character) and a longer C=O distance (i.e., pronounced single-bond character) (Fig. S5, *b* and *c*). Note that the O–D and C=O bond distances are highly correlated with *ν*_O–D_(donor) and *ν*_C=O_(donor), respectively (Fig. S6). Thus, a positive correlation exists between *ν*_C=O_(donor) and Δp*K*_a_(donor … acceptor) (Fig. 7).

However, the correlation between *ν*_C=O_(Asp85) and Δp*K*_a_(Asp85-COOH … O_W401_H_2_) in the N′-state BR is not consistent with the correlation between *ν*_C=O_(Glu46) and Δp*K*_a_(Glu46-COOH … ^–^O-*p*CA) in the ground-state PYP (Fig. 3 *b*), even though the correlation between *ν*_O–D_(Asp85) and Δp*K*_a_(Asp85-COOD … O_W401_D_2_) is consistent with the correlation between *ν*_O–D_(Glu46) and Δp*K*_a_(Glu46-COOD … ^–^O-*p*CA) (Fig. 3 *a*). The weaker correlation between *ν*_C=O_(donor) and Δp*K*_a_(donor … acceptor) is due to the following factors: 1) O–D for *ν*_O–D_(donor) directly donates an H-bond to the acceptor group, whereas the C=O bond for *ν*_C=O_(donor) does not directly contain a proton. Nevertheless, the *ν*_C=O_(donor) increases/decreases as the *ν*_O–D_(donor) increases/decreases (Fig. S5) (22). 2) *ν*_C=O_(donor) may additionally be shifted due to an H-bond interaction with an adjacent group or electrostatic interactions with polar and charged groups (25,60). Thus, the correlation between *ν*_C=O_(donor) and Δp*K*_a_(donor … acceptor) is specific to the local protein environment.

### Residues that decrease ***ν***_O–D_(W402) and migrate the proton toward Asp85 in the ground-state BR

**Figure 8:**
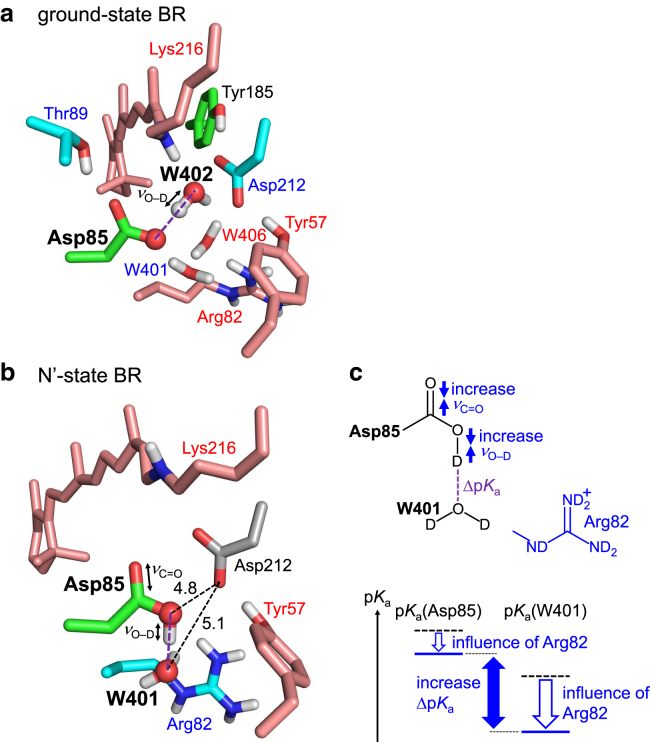
Key residues that influence *ν*_O–D_ and *ν*_C=O_ in BR. (*a*) QM/MM-optimized structure of the ground-state BR. Residues and water molecules that decrease and increase *ν*_O–D_(W402) are red and blue labeled, respectively. (*b*) QM/MM-optimized structure of the N′-state BR. Residues that decrease and increase *ν*_O–D_(Asp85) are red and blue labeled, respectively. Values indicate distances (Å). (*c*) Schematic illustration of the influence of Arg82: (*top*) on *ν*_O–D_(Asp85) and *ν*_C=O_(Asp85); (*bottom*) on Δp*K*_a_(Asp85-COOD … O_W401_D_2_) in the N′-state BR. To see this figure in color, go online.

W402 donates H-bonds to Asp85 and Asp212 near the Schiff base (Fig. 8 *a*). p*K*_a_(Asp212) is lower than p*K*_a_(Asp85), as 1) Tyr57 and Tyr185 donate H-bonds to Asp212 and 2) Arg82 decreases p*K*_a_(Asp212) more significantly than p*K*_a_(Asp85) (14). Thus, Δp*K*_a_(DO_W402_–D … ^–^OOC-Asp85) is smaller than Δp*K*_a_(DO_W402_–D … ^–^OOC-Asp212) (i.e., the release of the proton toward Asp212 is more energetically uphill than that toward Asp85) and *ν*_O–D_(O_W402_–D … Asp85) (2171 cm^−1^ (9,10)) is significantly lower than *ν*_O–D_(O_W402_–D … Asp212) (2636 cm^−1^ (9)). This is why the H-bond between W402 and Asp85 has the lowest *ν*_O–D_(water) in BR (9,10,13,14), the likely origin of the proton-pumping activity. The present QM/MM calculation indicates that the positively charged retinal Schiff base, the H-bond donor to W402, contributes to the decrease in p*K*_a_(W402) with respect to p*K*_a_(Asp85) and migration of the proton toward Asp85 most significantly among all sites in the ground-state BR (Tables 2 and S3). Arg82 and Tyr57, which decrease p*K*_a_(Asp212) with respect to p*K*_a_(Asp85), also contribute to migration of the W402 proton toward Asp85 and the decrease in *ν*_O–D_(O_W402_–D … Asp85) (Table 2).

**Table 2:** Residues and water molecules that decrease/increase *ν*_O–D_(W402) (cm^−1^) and Δp*K*_a_(DO_W402_–D … ^–^OOC-Asp85) most significantly in the ground-state BR

Decreasing *ν*_O–D_(W402)	Δ*ν*_O–D_	ΔΔp*K*_a_	Increasing *ν*_O–D_(W402)	Δ*ν*_O–D_	ΔΔp*K*_a_
Proton transfer (PT) toward Asp85			PT toward W402		
Retinal Schiff base	−178	−3.5	Asp212	286	4.7
Arg82	−34	−0.7	H_2_O-401	55	1.0
Tyr57	−26	−0.7	Thr89	42	0.8
H_2_O-406	−17	−0.5			

Thr89 increases *ν*_O–D_(O_W402_–D … Asp85), as it donates an H-bond to Asp85 and inhibits migration of the W402 proton toward Asp85 (Table 2). Indeed, *ν*_O–D_(W402) decreases by ∼80 cm^−1^ upon mutation of Thr89 to alanine (10), which is consistent with the present result.

### Protonated Asp85 in the N′-state BR

Asp85 is protonated in the N′-state BR (22,61), which is a prerequisite for the subsequent release of the proton toward the extracellular side. Although Arg82 decreases p*K*_a_(Asp85), Arg82 is the residue that contributes most to protonation of Asp85 among all sites in the N′-state BR, as suggested by the increase in *ν*_O–D_(Asp85) (Tables 3 and S4) and *ν*_C=O_(Asp85) (Table 4). In the N′-state BR, Arg82 is closer to W401 (6.0 Å) than Asp85 (7.8 Å) (Fig. 8 *b*). Arg82 decreases p*K*_a_(W401) more significantly than p*K*_a_(Asp85) and increases Δp*K*_a_(Asp85-COOD … O_W401_D_2_) (Fig. 8 *c*). Eventually, Arg82 contributes to the fixation of the proton at the H-bond donor (Asp85) moiety, increasing *ν*_O–D_(Asp85) and *ν*_C=O_(Asp85) (Tables 3 and 4).

**Table 3:** Residues that decrease/increase *ν*_O–D_(Asp85) (cm^−1^) and Δp*K*_a_(Asp85-COOD … O_W401_D_2_) most significantly in the N′-state BR

Decreasing *ν*_O–D_(Asp85)	Δ*ν*_O–D_	ΔΔp*K*_a_	Increasing *ν*_O–D_(Asp85)	Δ*ν*_O–D_	ΔΔp*K*_a_
PT toward W401			PT toward Asp85		
Retinal Schiff base	−67	−1.8	Arg82	39	1.2
Tyr57	−12	−0.4	Arg7	12	0.4
Glu194	−12	−0.4			
Asp212	−4	−0.3

**Table 4:** Residues that decrease/increase *ν*_C=O_(Asp85) (cm^−1^) and Δp*K*_a_(Asp85-COOH … O_W401_H_2_) most significantly in the N′-state BR

Decreasing *ν*_C=O_(Asp85)	Δ*ν*_C=O_	ΔΔp*K*_a_	Increasing *ν*_C=O_(Asp85)	Δ*ν*_C=O_	ΔΔp*K*_a_
PT toward W401			PT toward Asp85		
Retinal Schiff base	−36	−1.8	Asp212	12	−0.3
			Arg82	7	1.2
			Arg7	3	0.4

In both the ground and N′-state BRs, the Schiff base is closer to the H-bond donor (W402 in the ground-state BR/Asp85 in the N′-state BR) than the acceptor (Asp85 in the ground-state BR/W401 in the N′-state BR) (Fig. 8, *a* and *b*). The Schiff base decreases p*K*_a_(donor) more significantly than p*K*_a_(acceptor) and decreases Δp*K*_a_(donor … acceptor). Thus, the Schiff base contributes to the migration of the proton from the H-bond donor toward the acceptor, decreasing *ν*_O–D_(W402) in the ground-state BR (Table 2) and *ν*_O–D_(Asp85) and *ν*_C=O_(Asp85) in the N′-state BR (Tables 3 and 4).

As Asp212 is equidistant from Asp85 (4.8 Å) and W401 (5.1 Å) in the N′-state BR (Fig. 8 *b*), the increase in p*K*_a_(Asp85) caused by Asp212 is the same as the increase in p*K*_a_(W401) caused by Asp212. Thus, Asp212 does not significantly contribute to Δp*K*_a_(Asp85-COOD … O_W401_D_2_) (−0.3) and *ν*_O–D_(Asp85) (−4 cm^−1^) irrespective of a negatively charged residue (Table 3).

However, Asp212 increases *ν*_C=O_(Asp85) by 12 cm^−1^ (Table 4), because the electrostatic interaction between Asp212 and Asp85 specifically decreases the C=O distance in Asp85 (Fig. 8 *b*). The present case indicates that C=O distances are additionally affected by the local protein environment, which is a reason for the weak correlation between *ν*_C=O_(donor) and Δp*K*_a_(donor … acceptor).

### Protonated Glu46 in the ground-state PYP

Protonated Glu46 is a prerequisite for the formation of a stable H-bond with deprotonated *p*CA in the ground-state PYP (33,34,35,36,37,38,39). The present result indicates that the protonated state of Arg52 (33) and the H-bond donation from Tyr42 to *p*CA contribute most to protonation of Glu46 in the ground-state PYP (Tables 5, 6, and S5). In particular, Tyr42 contributes to an increase in *ν*_C=O_(Glu46) of 6 cm^−1^ in the ground-state PYP (Table 6). Consistently, FTIR studies suggested that *ν*_C=O_(Glu46) decreases by 12 cm^−1^ upon mutation of Tyr42 to phenylalanine (62).

**Table 5:** Residues that decrease/increase *ν*_O–D_(Glu46) (cm^−1^) and Δp*K*_a_(Glu46-COOD … ^–^O-*p*CA) most significantly in the ground-state PYP

Decreasing *ν*_O–D_(Glu46)	Δ*ν*_O–D_	ΔΔp*K*_a_	Increasing *ν*_O–D_(Glu46)	Δ*ν*_O–D_	ΔΔp*K*_a_
PT toward *p*CA			PT toward Glu46		
Asp97	−193	−2.3	Arg52	199	1.9
Arg124	−75	−1.1	Tyr42	134	1.4
Asp71	−50	−0.8	Asp48	84	1.1

**Table 6:** Residues that decrease/increase *ν*_C=O_(Glu46) (cm^−1^) and Δp*K*_a_(Glu46-COOH … ^–^O-*p*CA) most significantly in the ground-state PYP

Decreasing *ν*_C=O_(Glu46)	Δ*ν*_C=O_	ΔΔp*K*_a_	Increasing *ν*_C=O_(Glu46)	Δ*ν*_C=O_	ΔΔp*K*_a_
PT toward *p*CA			PT toward Glu46		
Arg124	−12	−1.1	Arg52	13	1.9
Asp97	−9	−2.3	Asp48	6	1.1
Lys123	−3	−0.6	Tyr42	6	1.4

**Figure 9:**
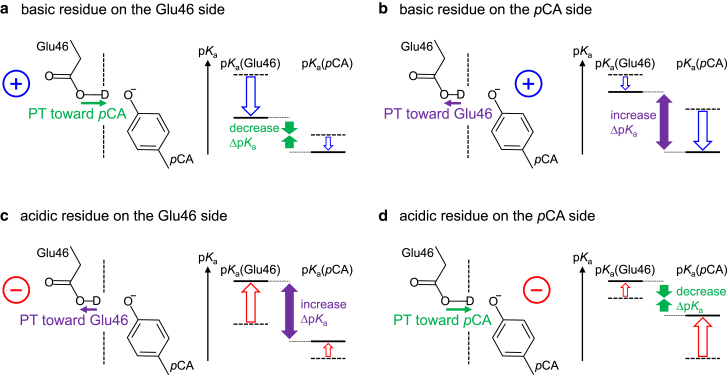
Electrostatic influence and shift in Δp*K*_a_(Glu46 … *p*CA) in the ground-state PYP. (*a*) Decrease in Δp*K*_a_(Glu46 … *p*CA) due to the influence of a basic residue on the Glu46 side. (*b*) Increase in Δp*K*_a_(Glu46 … *p*CA) due to the influence of a basic residue on the *p*CA side. The blue open arrows indicate the influence of a basic residue on p*K*_a_(Glu46) and p*K*_a_(*p*CA). (*c*) Increase in Δp*K*_a_(Glu46 … *p*CA) due to the influence of an acidic residue on the Glu46 side. (*d*) Decrease in Δp*K*_a_(Glu46 … *p*CA) due to the influence of an acidic residue on the *p*CA side. The red open arrows indicate the influence of an acidic residue on p*K*_a_(Glu46) and p*K*_a_(*p*CA). The green and purple closed arrows indicate the decrease and increase in Δp*K*_a_(Glu46 … *p*CA) due to the electrostatic influence of the residue, respectively. To see this figure in color, go online.

While estimating vibrational frequencies solely from the “p*K*_a_” (of the H-bond donor or acceptor moiety) may be adequate as an initial approach in some cases (e.g., (9,63)), the consideration of “Δp*K*_a_” (of the H-bond donor and acceptor moieties) is required for a more comprehensive and detailed discussion as presented in this study. Importantly, the shifts in *ν*_O–D_(donor) and *ν*_C=O_(donor) caused by an external charged group does not merely originate from the shift in p*K*_a_(donor) alone, but from the total shift in Δp*K*_a_(donor … acceptor). This is because the shifts in *ν*_O–D_(donor) and *ν*_C=O_(donor) originate not only from the electrostatic interaction between the charged group and the H-bond donor moiety but also from the electrostatic interaction between the charged group and the H-bond acceptor moiety. Thus, the position of the charged group with respect to both the H-bond donor and acceptor groups are crucial to the resulting shifts in these properties (Fig. 9). Positively charged basic residues commonly decrease p*K*_a_(Glu46). However, Lys123 and Arg124 decrease Δp*K*_a_(Glu46 … *p*CA), whereas Arg52 increases Δp*K*_a_(Glu46 … *p*CA) (Tables 5 and 6). Lys123 and Arg124 are closer to Glu46 than *p*CA (Fig. S7). Lys123 and Arg124 decrease p*K*_a_(Glu46) more significantly than p*K*_a_(*p*CA), which decreases Δp*K*_a_(Glu46 … *p*CA), *ν*_O–D_(Glu46), and *ν*_C=O_(Glu46) (Fig. 9 *a*; Tables 5 and 6). On the other hand, Arg52 is closer to *p*CA than Glu46 (Fig. S7). Arg52 decreases p*K*_a_(*p*CA) more significantly than p*K*_a_(Glu46), which increases Δp*K*_a_(Glu46 … *p*CA), *ν*_O–D_(Glu46), and *ν*_C=O_(Glu46) (Fig. 9 *b*; Tables 5 and 6). Thus, while Arg52 decreases p*K*_a_(Glu46), it conversely increases Δp*K*_a_(Glu46 … *p*CA), leading to the fixation of proton at the Glu46 moiety and consequently increasing *ν*_O–D_(Glu46) and *ν*_C=O_(Glu46).

The contribution of the acidic residue to Δp*K*_a_(Glu46 … *p*CA), *ν*_O–D_(Glu46), and *ν*_C=O_(Glu46) also depends on the position of the acidic residue (Fig. 9). Negatively charged acidic residues commonly increase p*K*_a_(Glu46). Asp48 is closer to Glu46 than *p*CA (Fig. S7). Asp48 increases p*K*_a_(Glu46) more significantly than p*K*_a_(*p*CA), which increases Δp*K*_a_(Glu46 … *p*CA), *ν*_O–D_(Glu46), and *ν*_C=O_(Glu46) (Fig. 9 *c*; Tables 5 and 6). On the other hand, Asp71 and Asp97 are closer to *p*CA than Glu46 (Fig. S7). Asp71 and Asp97 increase p*K*_a_(*p*CA) more significantly than p*K*_a_(Glu46), which decreases Δp*K*_a_(Glu46 … *p*CA), *ν*_O–D_(Glu46), and *ν*_C=O_(Glu46) (Fig. 9 *d*; Tables 5 and 6). Thus, while Asp71 and Asp97 increase p*K*_a_(Glu46), they conversely decrease Δp*K*_a_(Glu46 … *p*CA), leading to the proton release toward the *p*CA moiety and consequently decreasing *ν*_O–D_(Glu46) and *ν*_C=O_(Glu46).

## Conclusions

The O–D stretching vibrational frequency of the H-bond donor [*ν*_O–D_(donor)] is correlated with the p*K*_a_ difference between the H-bond donor and acceptor [Δp*K*_a_(donor … acceptor)] in different protein environments, as well as in the absence of a protein environment (Fig. 3 *a*). This correlation suggests that *ν*_O–D_(donor) can be a useful tool for estimating Δp*K*_a_(donor … acceptor) in proteins (Eq. 2). On the other hand, the correlation between *ν*_C=O_(donor) and Δp*K*_a_(donor … acceptor) is weaker, mainly due to the fact that the C=O bond does not directly contain a proton (Fig. 3 *b*). However, the shift in *ν*_C=O_(donor) may still be used to estimate the shift in Δp*K*_a_(donor … acceptor), especially when comparing similar protein environments (e.g., comparison between reaction intermediate states in the same protein). It should be noted that the location of a charged/polar residue with respect to the H-bond donor and acceptor groups also plays a crucial role in the direction of the shifts in *ν*_O–D_(donor), *ν*_C=O_(donor), and Δp*K*_a_(donor … acceptor) (Figs. 8 and 9). For instance, a basic residue near the H-bond acceptor group would decrease p*K*_a_(acceptor) more significantly than p*K*_a_(donor), leading to an increase in Δp*K*_a_(donor … acceptor) and *ν*_O–D_(donor)/*ν*_C=O_(donor). Conversely, an acidic residue near the H-bond acceptor group would increase p*K*_a_(acceptor) more significantly than p*K*_a_(donor), resulting in a decrease in Δp*K*_a_(donor … acceptor) and *ν*_O–D_(donor)/*ν*_C=O_(donor). These results provide insights into the factors that contribute to proton transfer in protein environments and suggest that experimentally measured *ν*_O–D_(donor) and *ν*_C=O_(donor) values can be used to estimate Δp*K*_a_(donor … acceptor), and provides a useful framework for understanding and investigating proton transfer events in proteins.

## Author contributions

H.I. designed the research. M.T., K.S., and H.I performed the research. M.T., K.S., and H.I. analyzed data. M.T. and H.I. wrote the paper.

## Supporting material

Document S1. Figures S1–S7 and Tables S1–S5

Data S1. QM/MM-optimized geometry

Document S2. Article plus supporting material

## References

[1] Oesterhelt D., Stoeckenius W. (1971). Rhodopsin-like protein from the purple membrane of *Halobacterium halobium*. Nat. New Biol..

[2] Luecke H., Schobert B., Lanyi J.K. (1999). Structure of bacteriorhodopsin at 1.55 Å resolution. J. Mol. Biol..

[3] Kandori H., Yamazaki Y., Maeda A. (1995). Water-mediated proton-transfer in proteins: an FTIR study of bacteriorhodopsin. J. Am. Chem. Soc..

[4] Maeda A., Sasaki J., Lanyi J.K. (1994). Interaction of aspartate-85 with a water molecule and the protonated Schiff base in the L intermediate of bacteriorhodopsin: a Fourier-transform infrared spectroscopic study. Biochemistry.

[5] Ernst O.P., Lodowski D.T., Kandori H. (2014). Microbial and animal rhodopsins: structures, functions, and molecular mechanisms. Chem. Rev..

[6] Engelhard M., Gerwert K., Siebert F. (1985). Light-driven protonation changes of internal aspartic acids of bacteriorhodopsin: an investigation by static and time-resolved infrared difference spectroscopy using [4-^13^C] aspartic acid labeled purple membrane. Biochemistry.

[7] Kandori H. (2004). Hydration switch model for the proton transfer in the Schiff base region of bacteriorhodopsin. Biochim. Biophys. Acta.

[8] Benedict W.S., Gailar N., Plyler E.K. (1956). Rotation-vibration spectra of deuterated water vapor. J. Chem. Phys..

[9] Shibata M., Kandori H. (2005). FTIR studies of internal water molecules in the Schiff base region of bacteriorhodopsin. Biochemistry.

[10] Shibata M., Tanimoto T., Kandori H. (2003). Water molecules in the Schiff base region of bacteriorhodopsin. J. Am. Chem. Soc..

[11] Kandori H. (2020). Structure/function study of photoreceptive proteins by FTIR spectroscopy. Bull. Chem. Soc. Jpn..

[12] Muroda K., Nakashima K., Kandori H. (2012). Protein-bound water as the determinant of asymmetric functional conversion between light-driven proton and chloride pumps. Biochemistry.

[13] Hayashi S., Ohmine I. (2000). Proton transfer in bacteriorhodopsin: structure, excitation, IR spectra, and potential energy surface analyses by an *ab initio* QM/MM method. J. Phys. Chem. B.

[14] Saito K., Kandori H., Ishikita H. (2012). Factors that differentiate the H-bond strengths of water near the Schiff bases in bacteriorhodopsin and *Anabaena* sensory rhodopsin. J. Biol. Chem..

[15] Perrin C.L., Nielson J.B. (1997). "Strong" hydrogen bonds in chemistry and biology. Annu. Rev. Phys. Chem..

[16] Ishikita H., Saito K. (2014). Proton transfer reactions and hydrogen-bond networks in protein environments. J. R. Soc. Interface.

[17] Schutz C.N., Warshel A. (2004). The low barrier hydrogen bond (LBHB) proposal revisited: the case of the Asp...His pair in serine proteases. Proteins.

[18] Hasegawa N., Jonotsuka H., Takeda K. (2018). X-ray structure analysis of bacteriorhodopsin at 1.3 Å resolution. Sci. Rep..

[19] Schobert B., Brown L.S., Lanyi J.K. (2003). Crystallographic structures of the M and N intermediates of bacteriorhodopsin: assembly of a hydrogen-bonded chain of water molecules between Asp-96 and the retinal Schiff base. J. Mol. Biol..

[20] Anderson S., Crosson S., Moffat K. (2004). Short hydrogen bonds in photoactive yellow protein. Acta Crystallogr. D Biol. Crystallogr..

[21] Barth A. (2000). The infrared absorption of amino acid side chains. Prog. Biophys. Mol. Biol..

[22] Saito K., Xu T., Ishikita H. (2022). Correlation between C=O stretching vibrational frequency and p*K*_a_ shift of carboxylic acids. J. Phys. Chem. B.

[23] Braiman M.S., Bousché O., Rothschild K.J. (1991). Protein dynamics in the bacteriorhodopsin photocycle: submillisecond Fourier-transform infrared-spectra of the L-photointermediates, M-photointermediates, and N-photointermediates. Proc. Natl. Acad. Sci. USA.

[24] Xie A., Hoff W.D., Hellingwerf K.J. (1996). Glu46 donates a proton to the 4-hydroxycinnamate anion chromophore during the photocycle of photoactive yellow protein. Biochemistry.

[25] Takei K., Takahashi R., Noguchi T. (2008). Correlation between the hydrogen-bond structures and the C=O stretching frequencies of carboxylic acids as studied by density functional theory calculations: theoretical basis for interpretation of infrared bands of carboxylic groups in proteins. J. Phys. Chem. B.

[26] Meyer T.E., Yakali E., Tollin G. (1987). Properties of a water-soluble, yellow protein isolated from a halophilic phototrophic bacterium that has photochemical activity analogous to sensory rhodopsin. Biochemistry.

[27] Meyer T.E. (1985). Isolation and characterization of soluble cytochromes, ferredoxins and other chromophoric proteins from the halophilic phototrophic bacterium *Ectothiorhodospira halophila*. Biochim. Biophys. Acta.

[28] Sprenger W.W., Hoff W.D., Hellingwerf K.J. (1993). The eubacterium *Ectothiorhodospira halophila* is negatively phototactic, with a wavelength dependence that fits the absorption spectrum of the photoactive yellow protein. J. Bacteriol..

[29] Hoff W.D., Düx P., Hellingwerf K.J. (1994). Thiol ester-linked *p*-coumaric acid as a new photoactive prosthetic group in a protein with rhodopsin-like photochemistry. Biochemistry.

[30] Kort R., Vonk H., Hellingwerf K.J. (1996). Evidence for *trans-cis* isomerization of the *p*-coumaric acid chromophore as the photochemical basis of the photocycle of photoactive yellow protein. FEBS Lett..

[31] Hellingwerf K.J., Hendriks J., Gensch T. (2003). Photoactive yellow protein, a new type of photoreceptor protein: Will this “Yellow Lab” bring us where we want to go?. J. Phys. Chem. A.

[32] Hoff W.D., van Stokkum I.H., Hellingwerf K.J. (1994). Measurement and global analysis of the absorbance changes in the photocycle of the photoactive yellow protein from *Ectothiorhodospira halophila*. Biophys. J..

[33] Saito K., Ishikita H. (2012). Energetics of short hydrogen bonds in photoactive yellow protein. Proc. Natl. Acad. Sci. USA.

[34] Saito K., Ishikita H. (2012). H atom positions and nuclear magnetic resonance chemical shifts of short H bonds in photoactive yellow protein. Biochemistry.

[35] Saito K., Ishikita H. (2013). Formation of an unusually short hydrogen bond in photoactive yellow protein. Biochim. Biophys. Acta.

[36] Graen T., Inhester L., Groenhof G. (2016). The low barrier hydrogen bond in the photoactive yellow protein: A vacuum artifact absent in the crystal and solution. J. Am. Chem. Soc..

[37] Thomson B., Both J., Boxer S.G. (2019). Perturbation of short hydrogen bonds in photoactive yellow protein via noncanonical amino acid incorporation. J. Phys. Chem. B.

[38] Yoshimura Y., Oktaviani N.A., Mulder F.A.A. (2017). Unambiguous determination of protein arginine ionization states in solution by NMR spectroscopy. Angew. Chem. Int. Ed..

[39] Wang J. (2019). Visualization of H atoms in the X-ray crystal structure of photoactive yellow protein: Does it contain low-barrier hydrogen bonds?. Protein Sci..

[40] Tsujimura M., Tamura H., Ishikita H. (2022). Absorption wavelength along chromophore low-barrier hydrogen bonds. iScience.

[41] (2012). Jaguar. version 7.9.

[42] Brooks B.R., Bruccoleri R.E., Karplus M. (1983). CHARMM: a program for macromolecular energy, minimization, and dynamics calculations. J. Comput. Chem..

[43] MacKerell A.D., Bashford D., Karplus M. (1998). All-atom empirical potential for molecular modeling and dynamics studies of proteins. J. Phys. Chem. B.

[44] Jo S., Kim T., Im W. (2008). CHARMM-GUI: a web-based graphical user interface for CHARMM. J. Comput. Chem..

[45] Bayly C.I., Cieplak P., Kollman P.A. (1993). A well-behaved electrostatic potential based method using charge restraints for deriving atomic charges: the RESP model. J. Phys. Chem..

[46] Rabenstein B., Ullmann G.M., Knapp E.W. (1998). Energetics of electron-transfer and protonation reactions of the quinones in the photosynthetic reaction center of *Rhodopseudomonas viridis*. Biochemistry.

[47] Rabenstein B., Ullmann G.M., Knapp E.-W. (1998). Calculation of protonation patterns in proteins with structural relaxation and molecular ensembles - application to the photosynthetic reaction center. Eur. Biophys. J..

[48] Bashford D., Karplus M. (1990). p*K*_a_'s of ionizable groups in proteins: atomic detail from a continuum electrostatic model. Biochemistry.

[49] Nozaki Y., Tanford C. (1967). Acid-base titrations in concentrated guanidine hydrochloride. Dissociation constants of the guanidinium ion and of some amino acids. J. Am. Chem. Soc..

[50] Tanokura M. (1983). ^1^H nuclear magnetic resonance titration curves and microenvironments of aromatic residues in bovine pancreatic ribonuclease A. J. Biochem..

[51] Tanokura M. (1983). ^1^H-NMR study on the tautomerism of the imidazole ring of histidine residues: I. Microscopic p*K* values and molar ratios of tautomers in histidine-containing peptides. Biochim. Biophys. Acta.

[52] Tanokura M. (1983). ^1^H-NMR study on the tautomerism of the imidazole ring of histidine residues: II. Microenvironments of histidine-12 and histidine-119 of bovine pancreatic ribonuclease A. Biochim. Biophys. Acta.

[53] Rabenstein B., Knapp E.-W. (2001). Calculated pH-dependent population and protonation of carbon-monoxy-myoglobin conformers. Biophys. J..

[54] (2012). QSite. Version 5.8.

[55] Jorgensen W.L., Maxwell D.S., Tirado-Rives J. (1996). J. Development and testing of the OPLS all-atom force field on conformational energetics and properties of organic liquids. J. Am. Chem. Soc..

[56] Scott A.P., Radom L. (1996). Harmonic vibrational frequencies: an evaluation of Hartree-Fock, Møller-Plesset, quadratic configuration interaction, density functional theory, and semiempirical scale factors. J. Phys. Chem..

[57] Ikeda T., Saito K., Ishikita H. (2017). The existence of an isolated hydronium ion in the interior of proteins. Angew. Chem. Int. Ed..

[58] (2022). CRC Handbook of Chemistry and Physics.

[59] Mikenda W. (1986). Stretching frequency versus bond distance correlation of O–D(H), Y (Y = N, O, S, Se, Cl, Br, I) hydrogen-bonds in solid hydrates. J. Mol. Struct..

[60] Nie B., Stutzman J., Xie A. (2005). A vibrational spectral maker for probing the hydrogen-bonding status of protonated Asp and Glu residues. Biophys. J..

[61] Dioumaev A.K., Brown L.S., Lanyi J.K. (2001). Coupling of the reisomerization of the retinal, proton uptake, and reprotonation of Asp-96 in the N photointermediate of bacteriorhodopsin. Biochemistry.

[62] Joshi C.P., Otto H., Heyn M.P. (2009). Strong hydrogen bond between glutamic acid 46 and chromophore leads to the intermediate spectral form and excited state proton transfer in the Y42F mutant of the photoreceptor photoactive yellow protein. Biochemistry.

[63] Nack M., Radu I., Heberle J. (2010). The DC gate in Channelrhodopsin-2: crucial hydrogen bonding interaction between C128 and D156. Photochem. Photobiol. Sci..

