# Supplementary material for: Stretching vibrational frequencies and pKa differences in H-bond networks of protein environments
Source: Biophys J. 2023 Oct 14;122(22):4336–47. doi: 10.1016/j.bpj.2023.10.012 (PMC10722396; doi:10.1016/j.bpj.2023.10.012)
Supplement: Document S1. Figures S1–S7 and Tables S1–S5 [file mmc1.pdf]

Biophysical Journal, Volume *122*

Supplemental information

Stretching vibrational frequencies and pK_a differences in H-bond networks of protein environments

Masaki Tsujimura, Keisuke Saito, and Hiroshi Ishikita

Supporting Information

Stretching vibrational frequencies and pK_a differences in H-bond networks of protein environments

Masaki Tsujimura [1*], Keisuke Saito [2,3], Hiroshi Ishikita [2,3*]

1) Department of Advanced Interdisciplinary Studies, The University of Tokyo, 4-6-1 Komaba, Meguro-ku, Tokyo 153-8904, Japan

2) Department of Applied Chemistry, The University of Tokyo, 7-3-1 Hongo, Bunkyo-ku, Tokyo 113-8654, Japan

3) Research Center for Advanced Science and Technology, The University of Tokyo, 4-6-1 Komaba, Meguro-ku, Tokyo 153-8904, Japan

Table S1. Residues with non-standard protonation states in the ground-state BR.

residue	protonation state
Asp96	protonated
Asp115	protonated
Glu194	protonated

Table S2. Residues with non-standard protonation states in the N'-state BR.

residue	protonation state
Asp85	protonated
Asp96	protonated
Asp115	protonated
Glu204	protonated

Table S3. Contributions of residues to ΔpK_a(DO$_{W402}$–D... $^-$OOC-Asp85) in the ground-state BR calculated by using a QM/MM approach and by solving the Poisson-Boltzmann equation (PBE) [a].

**decreasing Δp*K*a** [PT toward *p*CA]	**QM/MM**	**PBE**	**increasing Δp*K*a** [PT toward Glu46]	**QM/MM**	**PBE**
retinal Schiff base	−3.5	−4.2	Asp212	4.7	5.7
Arg82	−0.7	−1.3	Thr89	0.8	1.2
Tyr57	−0.7	−0.7			

[a] PBE was solved using the crystal structure (instead of using the QM/MM-optimized structure). The pK_a value employed as a reference for H_2O/OH^- is 15.74. Atomic charges of OH^- are obtained from the CHARMM22 parameter set.

Table S4. Contributions of residues to ΔpK_a(Asp85-COOD...$O_{W401}D_2$) in the N'-state BR calculated by using a QM/MM approach and by solving PBE [a].

**decreasing ΔpK_a** [PT toward *p*CA]	**QM/MM**	**PBE**	**increasing ΔpK_a** [PT toward Glu46]	**QM/MM**	**PBE**
retinal Schiff base	−1.8	−1.7	Arg82	1.2	2.4
Tyr57	−0.4	−0.3	Arg7	0.4	0.2
Glu194	−0.4	−0.5			
Asp212	−0.3	−1.1			

[a] PBE was solved using the crystal structure (instead of using the QM/MM-optimized structure). The pK_a value employed as a reference for H_3O^+/H_2O is −1.74. Atomic charges of H_3O^+ are obtained from Ref. [1].

Table S5. Contributions of residues to ΔpK_a(Glu46-COOD... $^{-}$O-*p*CA) in the ground-state PYP calculated by using a QM/MM approach and by solving PBE [a].

**decreasing ΔpK_a** [PT toward *p*CA]	**QM/MM**	**PBE**	**increasing ΔpK_a** [PT toward Glu46]	**QM/MM**	**PBE**
Asp97	−2.3	−0.6	Arg52	1.9	1.1
Arg124	−1.1	−0.2	Tyr42	1.4	1.2
Asp71	−0.8	−0.1	Asp48	1.1	0.2
Lys123	−0.6	−0.1			

[a] PBE was solved using the crystal structure (instead of using the QM/MM-optimized structure). The experimentally measured pK_a value employed as a reference is 8.8 for *p*CA [2]. Note that the contributions of residues to ν_{O-D}, $\nu_{C=O}$, and ΔpK_a values in the ground-state PYP (Tables 5–6) might potentially be overestimated due to the absence of the solvent effect in the present QM/MM calculations. In fact, when dielectric constants of 4 for the protein interior and 80 for bulk water are applied, the contributions of residues to ΔpK_a appear to be lower than those calculated using a QM/MM approach.

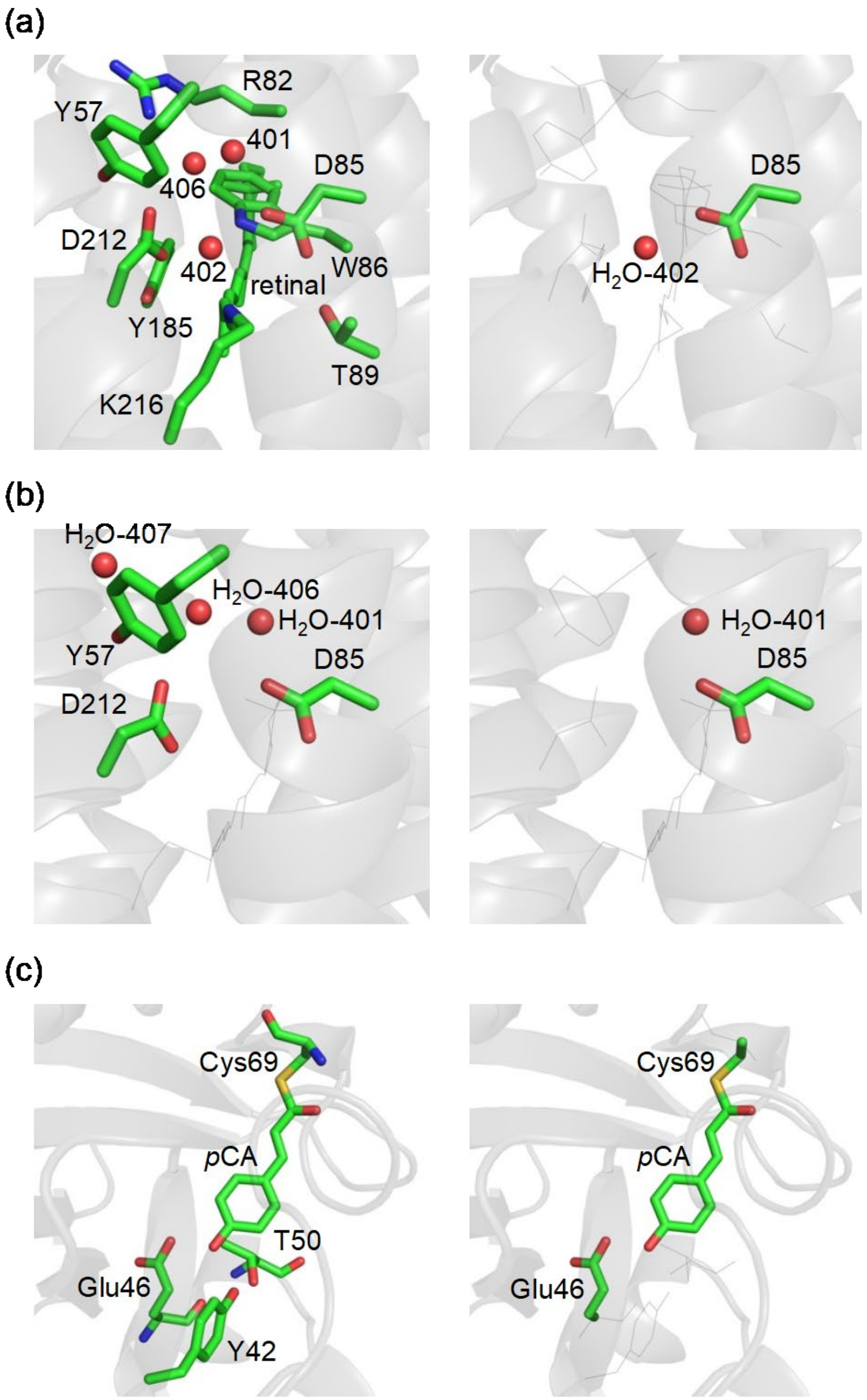

Figure S1. QM region defined in the present study. The initial QM region (left panel) and the redefined QM region (right panel). (a) The ground-state BR (PDB ID: 5ZIM [3]). (b) The N'-state BR (1P8U [4]). (c) The ground-state PYP (1OT9 [5]). Amino acid residues and cofactors in the QM region are shown as green sticks. Water oxygen atoms in the QM region are shown as red spheres.

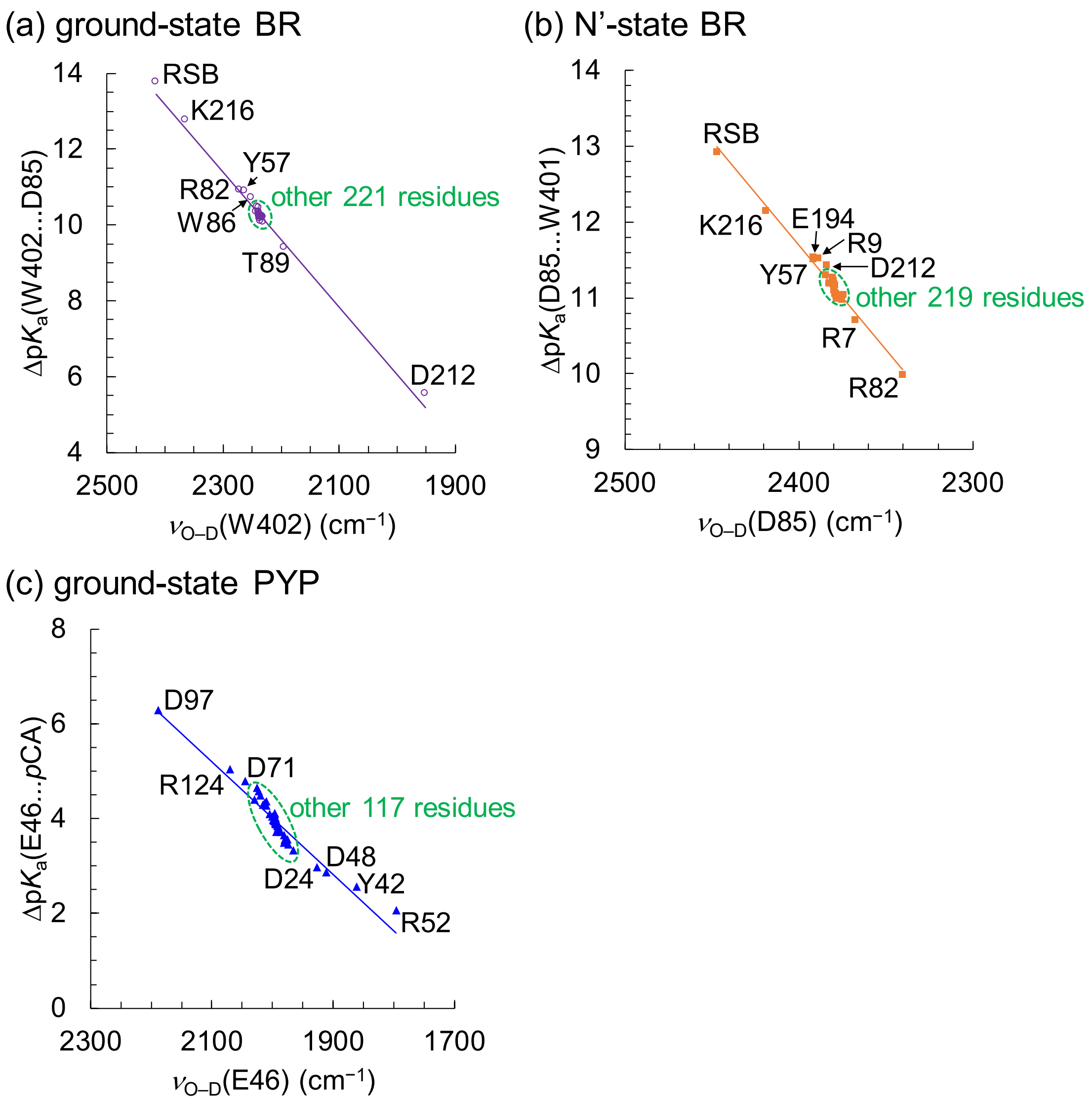

Figure S2. $\nu_{O\text{–}D}$(donor) and ΔpK_a calculated in the absence of the electrostatic influence of each residue. (a) Ground-state BR. RSB denotes retinal Schiff base. (b) N'-state BR. (c) Ground-state PYP.

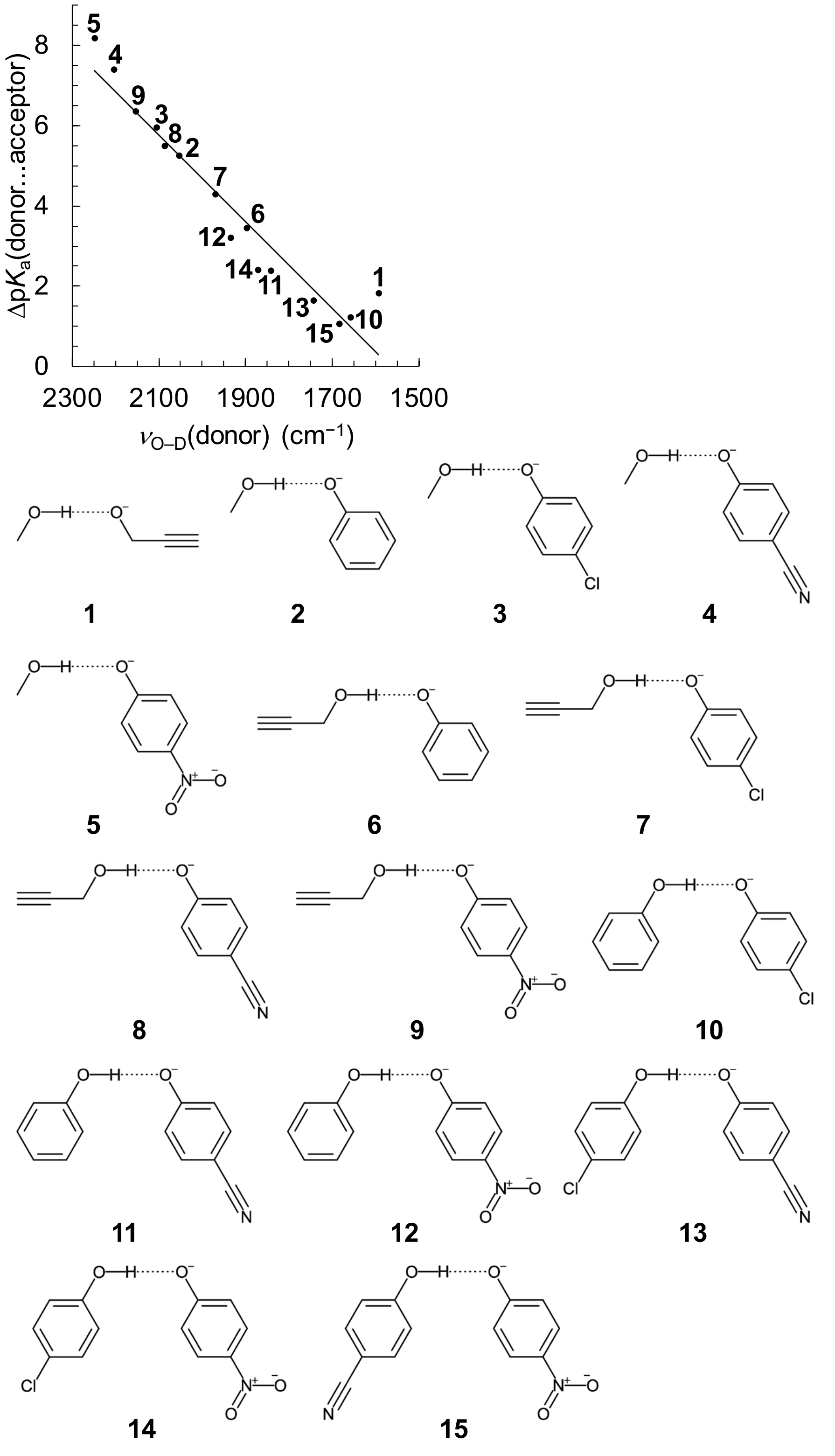

Figure S3. $\nu_{O\text{–}D}$(donor) and ΔpK_a of isolated 15 H-bond pairs. Correlation between $\nu_{O\text{–}D}$(donor) and ΔpK_a (upper panel). Structures of the H-bond pairs (lower panel).

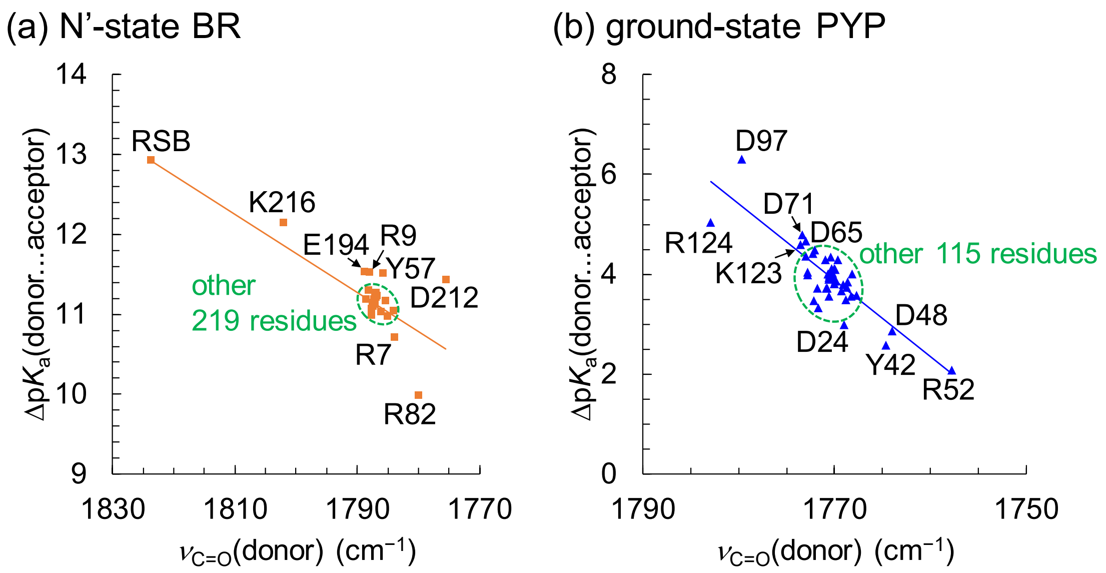

Figure S4. $\nu_{C=O}$(donor) and ΔpK_a calculated in the absence of the electrostatic influence of each residue. (a) N'-state BR. (b) Ground-state PYP.

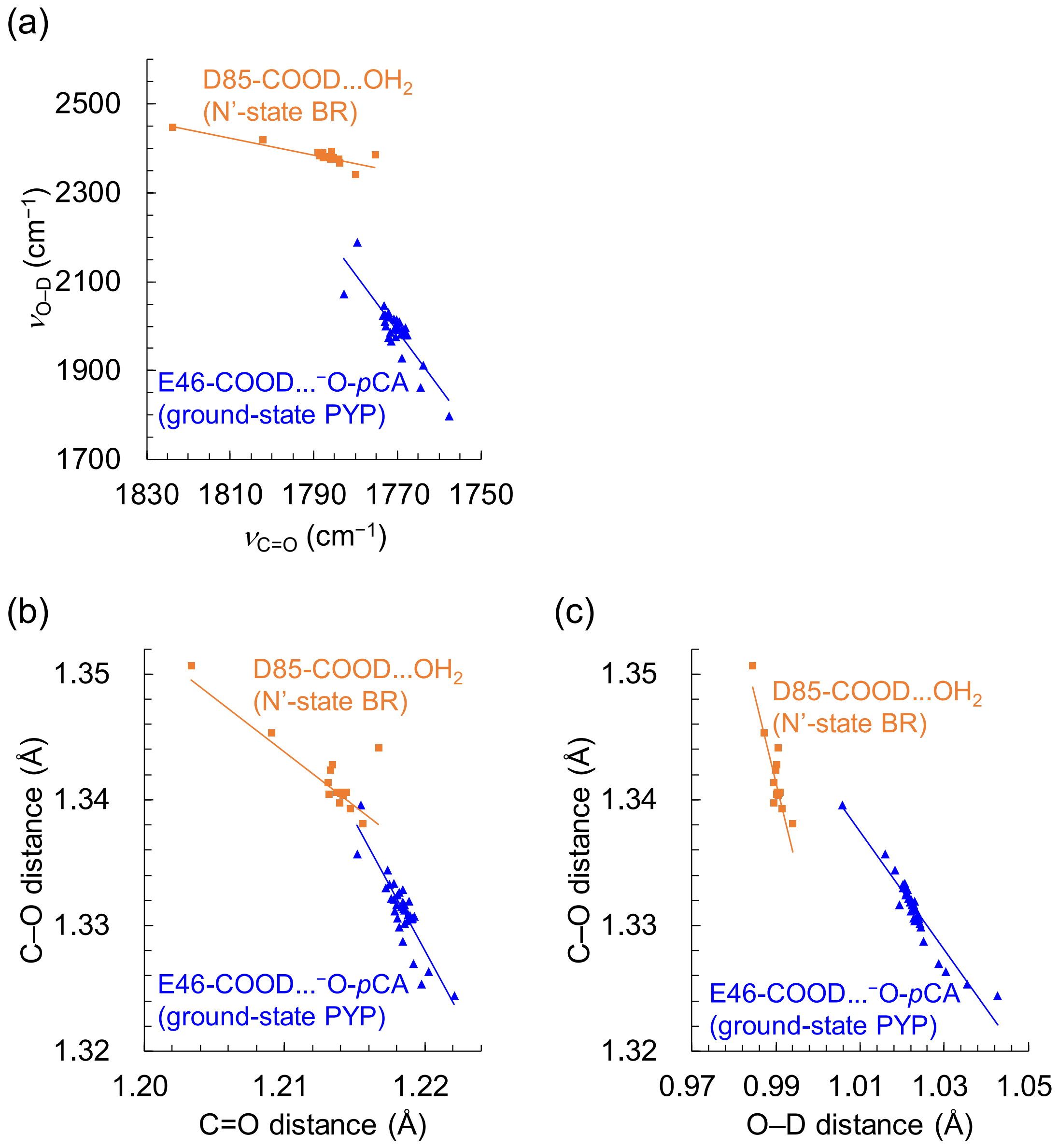

Figure S5. Relationship between $\nu_{C=O}$ and $\nu_{O–D}$ of protonated carboxylate. (a) $\nu_{C=O}$ and $\nu_{O–D}$: the N'-state BR (orange squares); the ground-state PYP (blue triangles). (b) The C=O distance and the C–O distance. (c) The C–O distance and the O–D distance.

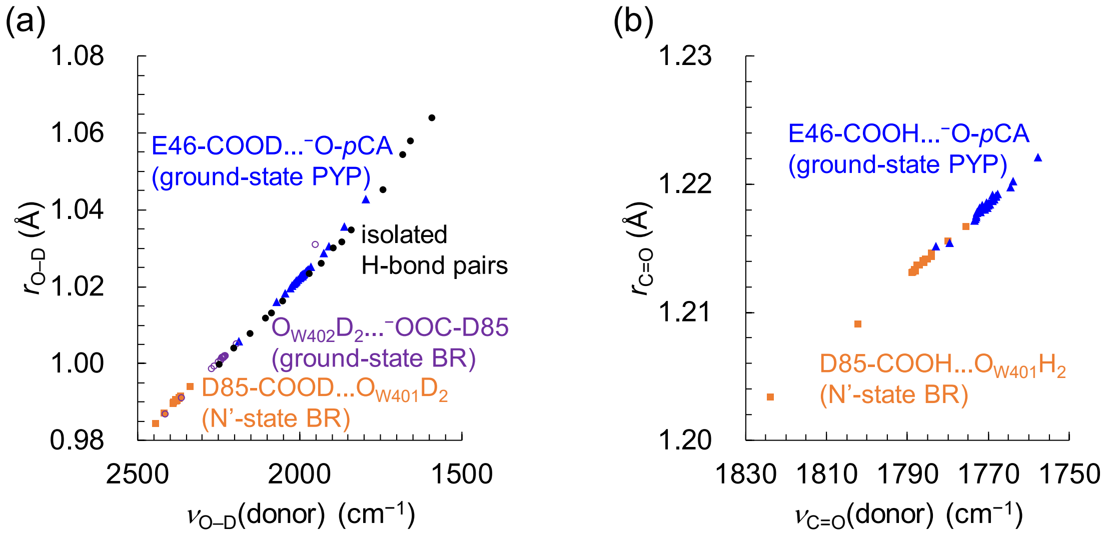

Figure S6. Stretching vibrational frequencies and bond distances obtained from the contribution of each residue. (a) $\nu_{O–D}$(donor) and the O–D bond distance ($r_{O–D}$) in the ground-state BR (purple open circles), the N'-state BR (orange squares), the ground-state PYP (blue triangles), and isolated 15 H-bond pairs listed in Table 1 (black closed circles). (b) $\nu_{C=O}$(donor) and the C=O bond distance ($r_{C=O}$) in the N'-state BR (orange squares) and the ground-state PYP (blue triangles).

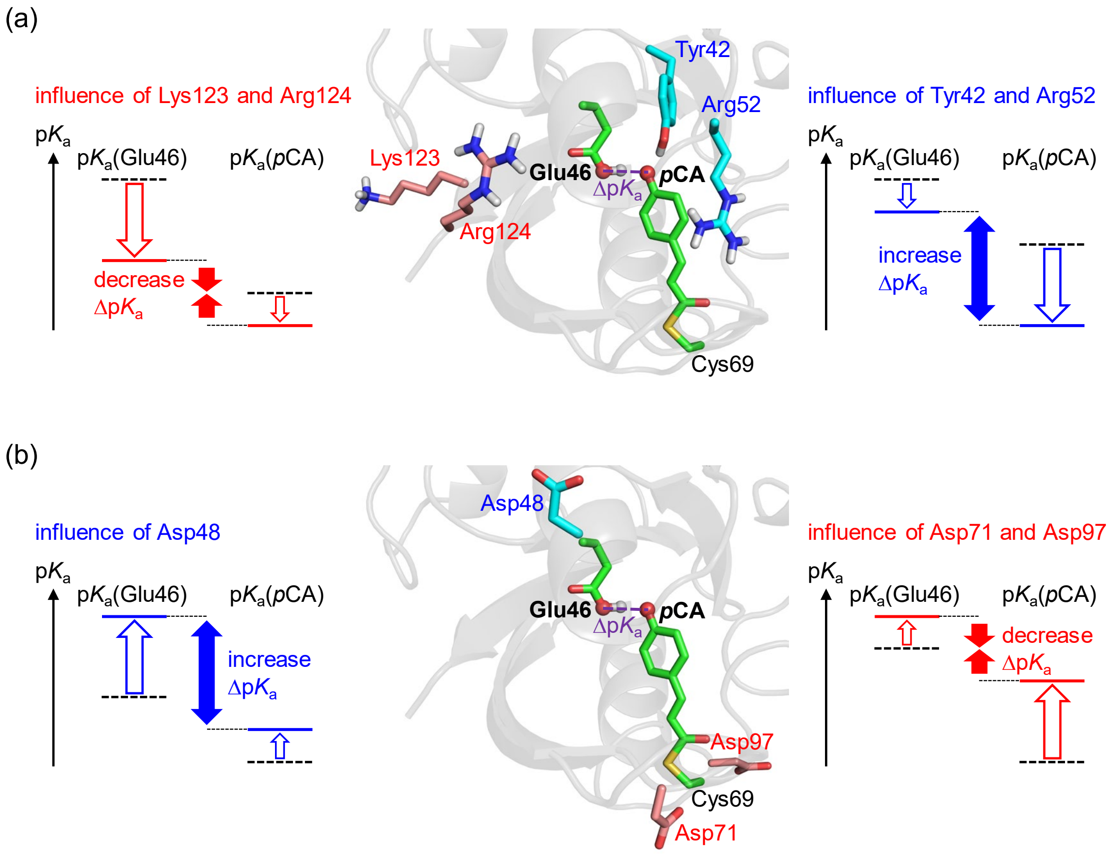

Figure S7. Key residues that influence ΔpK_a(Glu46...*p*CA), $\nu_{O\text{–}D}$(Glu46), and $\nu_{C=O}$(Glu46) in the ground-state PYP. (a) Influence of Tyr42 and basic residues. (b) Influence of acidic residues. Residues that decrease and increase ΔpK_a(Glu46...*p*CA)/ $\nu_{O\text{–}D}$(Glu46)/ $\nu_{C=O}$(Glu46) are red and blue labeled, respectively.

REFERENCES

1 Sagnella, D. E. & Voth, G. A. Structure and dynamics of hydronium in the ion channel gramicidin A. *Biophys. J.* **70**, 2043-2051 (1996).

2 Kroon, A. R. *et al.* Spectral tuning, fluorescence, and photoactivity in hybrids of photoactive yellow protein, reconstituted with native or modified chromophores. *J. Biol. Chem.* **271**, 31949-31956 (1996).

3 Hasegawa, N., Jonotsuka, H., Miki, K. & Takeda, K. X-ray structure analysis of bacteriorhodopsin at 1.3 Å resolution. *Sci. Rep.* **8**, 13123 (2018).

4 Schobert, B., Brown, L. S. & Lanyi, J. K. Crystallographic structures of the M and N intermediates of bacteriorhodopsin: assembly of a hydrogen-bonded chain of water molecules between Asp-96 and the retinal Schiff base. *J. Mol. Biol.* **330**, 553-570 (2003).

5 Anderson, S., Crosson, S. & Moffat, K. Short hydrogen bonds in photoactive yellow protein. *Acta Crystallogr. D Biol. Crystallogr.* **60**, 1008-1016 (2004).
