# Supplementary material for: Stretching vibrational frequencies and pKa differences in H-bond networks of protein environments
Source: Biophys J. 2023 Oct 14;122(22):4336–47. doi: 10.1016/j.bpj.2023.10.012 (PMC10722396; doi:10.1016/j.bpj.2023.10.012)
Supplement: Document S2. Article plus supporting material [file mmc3.pdf]

Biophysical Journal
Article

Biophysical Society

Stretching vibrational frequencies and pK_a differences in H-bond networks of protein environments

Masaki Tsujimura,[1,*] Keisuke Saito,[2,3] and Hiroshi Ishikita[2,3,*]
[1]Department of Advanced Interdisciplinary Studies, The University of Tokyo, Meguro-ku, Tokyo, Japan; [2]Department of Applied Chemistry, The University of Tokyo, Bunkyo-ku, Tokyo, Japan; and [3]Research Center for Advanced Science and Technology, The University of Tokyo, Meguro-ku, Tokyo, Japan

ABSTRACT The experimentally measured stretching vibrational frequencies of O–D [ν_{O-D}(donor)] and C=O [$\nu_{C=O}$(donor)] H-bond donor groups can provide valuable information about the H-bonds in proteins. Here, using a quantum mechanical/molecular mechanical approach, the relationship between these vibrational frequencies and the difference in pK_a values between H-bond donor and acceptor groups [ΔpK_a(donor … acceptor)] in bacteriorhodopsin and photoactive yellow protein environments was investigated. The results show that ν_{O-D}(donor) is correlated with ΔpK_a(donor … acceptor), regardless of the specific protein environment. $\nu_{C=O}$(donor) is also correlated with ΔpK_a(donor … acceptor), although the correlation is weak because the C=O bond does not have a proton. Importantly, the shifts in ν_{O-D}(donor) and $\nu_{C=O}$(donor) are not caused by changes in pK_a (donor) alone, but rather by changes in ΔpK_a(donor … acceptor). Specifically, a decrease in ΔpK_a(donor … acceptor) can lead to proton release from the H-bond donor group toward the acceptor group, resulting in shifts in the vibrational frequencies of the protein environment. These findings suggest that changes in the stretching vibrational frequencies, in particular ν_{O-D}(donor), can be used to monitor proton transfer in protein environments.

SIGNIFICANCE The stretching vibrational frequencies of O–D [ν_{O-D}(donor)] and C=O [$\nu_{C=O}$(donor)] bonds in proteins are valuable tools for understanding H-bond characteristics in proteins. This study used a quantum mechanical/molecular mechanical approach to investigate the relationship between these vibrational frequencies and the difference in pK_a between H-bond donor and acceptor groups [ΔpK_a(donor … acceptor)] in bacteriorhodopsin and photoactive yellow protein environments. The results reveal that ν_{O-D}(donor) is strongly correlated with ΔpK_a(donor … acceptor), providing a means of measuring pK_a values that are otherwise often difficult to directly obtain. This finding has significant implications for understanding biological processes such as proton transport, protein stability, and enzyme catalysis.

INTRODUCTION

Bacteriorhodopsin (BR) is a light-driven proton pump membrane protein (1,2). The chromophore is all-*trans* retinal covalently attached to Lys216 via the protonated Schiff base (Fig. 1 *a*) (2). The identification of water molecules at the Schiff base moiety was initially based on findings from Fourier transform infrared (FTIR) spectroscopy (3,4). These findings were subsequently confirmed in the X-ray crystal structure, revealing the presence of conserved water molecules identified as W402, W401, and W406 in the ground-state BR (2). In the ground state, the protonated Schiff base forms a pentagonal H-bond network with Asp85, Asp212, W402, W401, and W406 (Fig. 1 *a*) (2). Specifically, W402 accepts an H-bond from the Schiff base and also donates H-bonds to Asp85 and Asp212. W401 and W406 donate H-bonds to Asp85 and Asp212, respectively. The photocycle is initiated by the all-*trans* to 13-*cis* photoisomerization of the retinal chromophore (5). The proton released from the retinal Schiff base is transferred toward the extracellular side (5–7). Proton transfer can be investigated using FTIR spectroscopy and measuring the vibrational frequencies of H-bonds along the proton transfer pathway, because H-bond formation decreases the O–D stretching vibrational frequency for D_2O, $\nu_{O-D}(D_2O)$

Submitted April 6, 2023, and accepted for publication October 12, 2023.
*Correspondence: mtsujimura@protein.rcast.u-tokyo.ac.jp or hiro@appchem.t.u-tokyo.ac.jp
Editor: Jeremy David Schmit.
https://doi.org/10.1016/j.bpj.2023.10.012
© 2023 Biophysical Society.
This is an open access article under the CC BY license (http://creativecommons.org/licenses/by/4.0/).

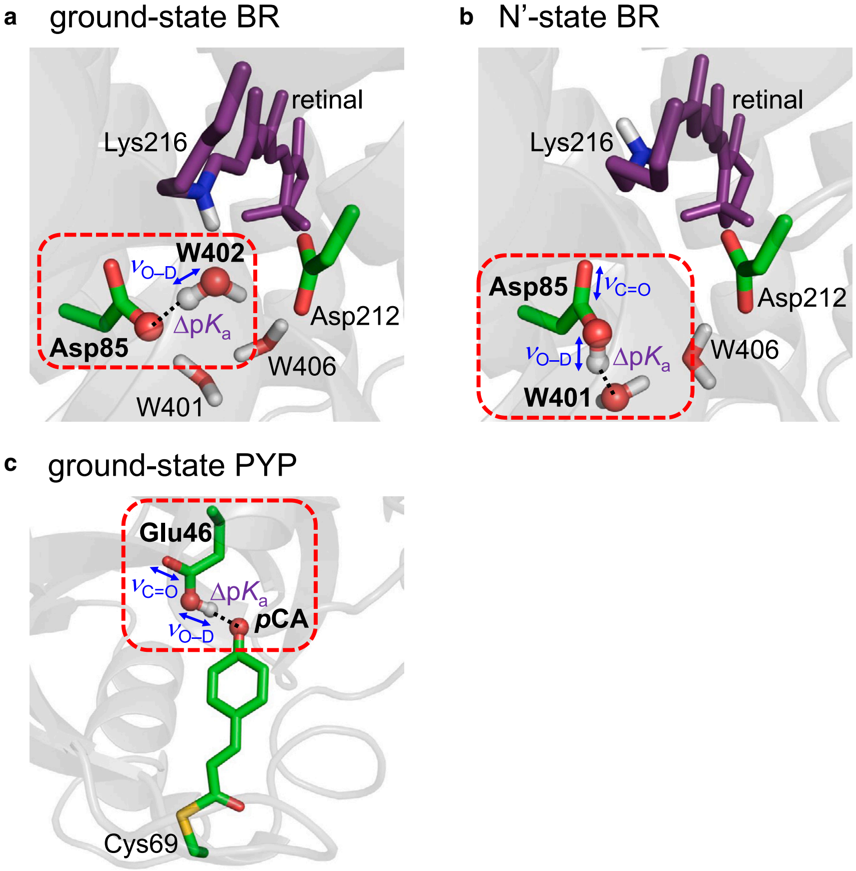

FIGURE 1 H-bonds investigated in this study. (*a*) [DO_{W402}–D … $^{-}$OOC-Asp85] in the ground-state BR (PDB: 5ZIM (18)). W402 serves as an H-bond donor, whereas Asp85 serves as an H-bond acceptor. (*b*) [Asp85-COOD … $O_{W401}D_2$] in the N′-state BR (PDB: 1P8U (19)). Asp85 serves as an H-bond donor, whereas W401 serves as an H-bond acceptor. (*c*) [Glu46-COOD … $^{-}$O-*p*CA] in the ground-state PYP (PDB: 1OT9 (20)). Glu46 serves as an H-bond donor, whereas *p*CA serves as an H-bond acceptor. Dotted lines indicate H-bonds. To see this figure in color, go online.

(= ~2700 cm^{-1} in the gas phase (8)) to ~2000–2600 cm^{-1} (9,10). A common feature of proton-pumping microbial rhodopsins is the presence of a water molecule with $\nu_{O\text{–}D}(D_2O)$ < 2400 cm^{-1} in the ground state (11,12). In particular, the lowest $\nu_{O\text{–}D}(D_2O)$ was observed at ~2200 cm^{-1} in the ground-state BR using FTIR spectroscopy (9,10). The water molecule (W402 (2)), which donates H-bonds to Asp85 and Asp212 near the Schiff base, has the lowest $\nu_{O\text{–}D}(D_2O)$ (Fig. 1 *a*) (9,10,13,14). In the two H-bonds, the W402 … Asp85 H-bond exhibits the lowest $\nu_{O\text{–}D}(D_2O)$ in the ground state BR, which plays a key role in the proton-pumping activity (9,10,13,14).

Before exploring the details of H-bonds, it is crucial to clarify the difference between "H^+ donor/acceptor" and "H-bond donor/acceptor." During proton transfer (i.e., transient state), the proton migrates from the "(more acidic) H^+ donor" moiety toward the "(more basic) H^+ acceptor" moiety. However, when considering H-bonds (i.e., resulting equilibrium state), the proton is predominantly localized at the "(more basic) H-bond donor" moiety rather than the "(more acidic) H-bond acceptor" moiety (15,16). Thus, in standard H-bonds, pK_a[(H-bond) donor] is higher than pK_a[(H-bond) acceptor], resulting in a positive pK_a difference between the H-bond donor and acceptor moieties [ΔpK_a(donor ... acceptor) = pK_a(donor) – pK_a(acceptor) > 0]. Notably, the concept of low-barrier hydrogen bonds arises when ΔpK_a(donor ... acceptor) = 0 (17). In this context, a decrease in $\nu_{O\text{–}D}(D_2O)$ corresponds to a decrease in ΔpK_a(donor … acceptor), leading to the migration of the proton toward the H-bond acceptor moiety (14,16). This explanation provides a comprehensive framework for understanding why low $\nu_{O\text{–}D}(D_2O)$ values are indicative of proton-pumping activity.

In BR, the H-bond network that proceeds from the intracellular side via the retinal Schiff base toward the extracellular side involves not only water molecules but also acidic residues, e.g., Asp85, Asp96, Glu194, and Glu204, all of which form proton transfer pathways (5–7). The C=O stretching vibrational frequency ($\nu_{C=O}$) for protonated carboxylate (1710–1770 cm^{-1}) is significantly higher than that for deprotonated carboxylate (~1400 and ~1570 cm^{-1} for the symmetric and asymmetric stretching vibrations, respectively) (21). Thus, from the remarkable shift in $\nu_{C=O}$, a change in the protonation state can be unambiguously detected, although carboxylate is more likely deprotonated due to the low pK_a value.

Notably, protonated carboxylate is reported in an intermediate state of BR. The release of the proton from protonated Asp96 toward the deprotonated retinal Schiff base occurs in the M to N transition (5,7). Accordingly, the adjacent counterion, Asp85, is fully protonated in the M state with

pK_a(Asp85) = 13 (22) and $\nu_{C=O}$(Asp85) = 1761 cm^{-1} (23) and partially protonated in the N state with pK_a(Asp85) = 7 (22) and $\nu_{C=O}$(Asp85) = 1756 cm^{-1} (Fig. 1 *b*) (23). The observed shift in $\nu_{C=O}$ is clearly distinct with respect to the spectral resolution, which is approximately in the range of 2–4 cm^{-1}, as reported in (23-25). pK_a(Asp85) = 7 in the N state was estimated based on the N′-state structure of the V49A mutant BR (22). The H-bond partner of Asp85 is a water molecule (W401 (19)) (Fig. 1 *b*). Because the Schiff base is closer to Asp85 (5.5 Å (19)) than to W401 (7.7 Å (19)), protonation of the Schiff base decreases pK_a(Asp85) more significantly than pK_a(W401). Thus, the decrease in pK_a(Asp85) in the M to N transition corresponds to the decrease in the pK_a difference between Asp85 and the H-bond acceptor water molecule [ΔpK_a(Asp85 … $O_{W401}H_2$)]. The M to N state transition is the only case among the photocycle intermediates in BR, where both $\nu_{C=O}$ and pK_a are reported.

A water-soluble photosensor protein, photoactive yellow protein (PYP) (26–28), is also one of the limited cases in which both pK_a and $\nu_{C=O}$ have been reported for some intermediate states during the photocycle. The *trans* to *cis* photoisomerization of *p*-coumaric acid (*p*CA) leads to the ground (pG) to pR state transition (29–32). In the pG state, protonated Glu46 donates an H-bond to *p*CA (Fig. 1 *c*) (33–39). In contrast, Glu46 forms a low-barrier H-bond with *p*CA in the pR state, which leads to proton transfer from Glu46 toward *p*CA (16,35,40). The pK_a difference between Glu46 and the H-bond acceptor *p*CA [ΔpK_a(Glu46 … *p*CA)] decreases from ~3 to ~0 in the pG to pR state transition (33,35), as $\nu_{C=O}$(Glu46) decreases from 1740 to 1732 cm^{-1} (24).

Both Asp85 in the M to N transition of BR and Glu46 in the pG to pR transition of PYP commonly show that $\nu_{C=O}$(donor) decreases as ΔpK_a(donor … acceptor) decreases. This tendency is consistent with that observed for saturated carboxylates in water (i.e., in the absence of the protein environment) (22). The decrease in ΔpK_a(donor … acceptor) corresponds to migration of the carboxylate proton toward the H-bond acceptor group due to formation of a low-barrier H-bond (16). The decrease in $\nu_{C=O}$(donor) also corresponds to the migration of the carboxylate proton toward the H-bond acceptor group (24,35). Based on these observations, ΔpK_a(donor … acceptor) may be estimated from experimentally measured $\nu_{C=O}$(donor) in the protein environment as long as the carboxylate exists in the same protein environment. This may also hold true for ΔpK_a(donor … acceptor) and experimentally measured ν_{O-D}(donor) in the protein environment. However, to the best of our knowledge, successful examples of estimating ΔpK_a(donor … acceptor) from experimentally measured ν_{O-D}(donor)/$\nu_{C=O}$(donor) in protein environments have not been reported. Thus, it remains unclear whether these vibrational frequencies are directly linked with the pK_a values of the H-bonds.

Here, we investigated how ν_{O-D}(donor) and $\nu_{C=O}$(donor) are linked with the pK_a difference between the H-bond donor and acceptor moieties in the protein environment. Since the H-bond pairs, in which both ΔpK_a(donor … acceptor) and ν_{O-D}(donor)/$\nu_{C=O}$(donor) are reported, are limited (e.g., Glu46 … *p*CA in the ground state of PYP (24,33,35)), the influences of the residues on ΔpK_a(donor … acceptor) and ν_{O-D}(donor)/$\nu_{C=O}$(donor) values for the focused H-bond pairs are also calculated in the protein environments, using a quantum mechanical/molecular mechanical (QM/MM) approach.

METHODS

Electrostatic calculations

The geometries of 15 H-bond pairs were optimized using the restricted density functional theory method with the B3LYP functional and the 6-31G* basis set, using the Jaguar program code (41). The H-bonds in the protein environments are investigated using the x-ray crystal structures of the ground-state BR (PDB: 5ZIM (18)), the N′-state BR (V49A mutant) (PDB: 1P8U (19)), and the ground-state PYP (PDB: 1OT9 (20)). The H atom positions were optimized using the CHARMM program code (42). Atomic charges and force field parameters were obtained from the CHARMM22 parameter set (43) for amino acids and water and CHARMM-GUI (44) for the retinal Schiff base. Atomic charges of *p*CA were determined by fitting the electrostatic potential using the restrained electrostatic potential procedure (45) as empirically recommended for the subsequent step (solving the linear Poisson-Boltzmann equation) (46,47). To ensure internal consistency, the restrained electrostatic potential charges were also used for constructing the initial geometry of *p*CA in this study. It is worth mentioning that *p*CA was included in the QM region and the geometry was QM/MM-optimized when calculating ν_{O-D}(donor), $\nu_{C=O}$(donor), and ΔpK_a(donor … acceptor) (see below).

Using the resulting atomic coordinates (Data S1), the protonation pattern in the ground- and N′-state BR was determined based on the electrostatic continuum model, solving the linear Poisson-Boltzmann equation with the MEAD program (48). The experimentally measured pK_a values employed as references were 12.0 for Arg, 4.0 for Asp, 9.5 for Cys, 4.4 for Glu, 10.4 for Lys, 9.6 for Tyr, (49), and 7.0 and 6.6 for the N_ε and N_δ atoms of His, respectively (50–52). The dielectric constants were set to 4 for the protein interior and 80 for water. All water molecules were considered implicitly. All computations were performed at 300 K, pH 7.0, and with an ionic strength of 100 mM. The linear Poisson-Boltzmann equation was solved using a three-step grid-focusing procedure at resolutions of 2.5, 1.0, and 0.3 Å. The ensemble of the protonation pattern was sampled by the Monte Carlo method with the Karlsberg program (53). The Monte Carlo sampling yielded the protonation probabilities of each residue. Residues whose calculated protonation states are nonstandard (i.e., protonated acidic and deprotonated basic groups) are listed in Tables S1 and S2. All titratable groups in the ground-state PYP were kept in the standard protonation states except for the Glu46 … *p*CA pair, as done in previous studies (40). For the Glu46 … *p*CA pair, the Glu46-COOH … $^{-}$O-*p*CA protonation pattern was adopted (40).

To calculate the pK_a values of the H-bond donor and acceptor under investigation and compare with the ΔpK_a(donor … acceptor) values calculated using a QM/MM approach (Tables S3–S5), the difference in the pK_a value in the protein relative to the reference system was calculated and then added to the known reference pK_a value. All other titratable sites were fully equilibrated to the protonation state of the target site during titration. A bias potential was applied to obtain an equal amount of both protonation states ([protonated] = [deprotonated]), yielding the pK_a value as the resulting bias potential.

QM/MM calculations

Geometry optimization

The geometry was optimized using a QM/MM approach. The restricted density functional theory method was employed with the B3LYP functional and the 6-31G* basis set, using the QSite program code (54). The QM region was first defined as follows (Fig. S1): the retinal, the side chains of Lys216 (Schiff base), Tyr57, Arg82, Asp85, Trp86, Thr89, Tyr185, and Asp212, and the three water molecules at the Schiff base moiety (W402, 401, and 406) for the ground-state BR; the side chains of Tyr57, Asp85, and Asp212 and the adjacent water molecules (W401, 406, and 407 (19)) for the N′-state BR; and the entireties of *p*CA, Glu46, Thr50, and Cys69 and the side chain of Tyr42 for the ground-state PYP. All atomic coordinates were fully relaxed in the QM region. In the MM region, the positions of H atoms were optimized using the OPLS2005 force field (55), while the positions of the heavy atoms were fixed. The protonation pattern of titratable residues was implemented in the atomic partial charges of the corresponding MM region.

Analysis of electrostatic contributions to stretching vibrational frequency and ΔpK_a values

The electrostatic contribution of a focusing residue in the MM region to ν_{O-D}(donor), $\nu_{C=O}$(donor), and ΔpK_a(donor … acceptor) in the QM region was evaluated through the following steps: 1) removing the atomic charges of the focusing side chain in the MM region, thereby eliminating its electrostatic influence (i.e., absence of the residue's electrostatic contribution), 2) reoptimizing the geometry using a QM/MM approach, and 3) calculating the resulting shifts in ν_{O-D}(donor), $\nu_{C=O}$(donor), and ΔpK_a(donor … acceptor). To facilitate this analysis, the QM region was redefined to solely include the H-bond donor and acceptor under investigation: W402 and the side chain of Asp85 for the ground-state BR; the side chain of Asp85 and W401 for the N′-state BR; and the entirety of *p*CA and the side chains of Glu46 and Cys69 for the ground-state PYP (Fig. S1). All atomic coordinates were fully relaxed in the QM region, while all atomic coordinates were fixed in the MM region. This approach was taken to avoid unrealistic displacements of superficial H atoms in the MM region caused by the removal of atomic charges, since such displacements might potentially introduce artifacts to the resulting shifts in ν_{O-D}(donor), $\nu_{C=O}$(donor), and ΔpK_a(donor … acceptor).

To obtain the potential energy profiles for proton transfer, the quantum-chemically optimized geometry was used as the initial geometry. The H atom under investigation was moved from the H-bond donor atom (D) toward the acceptor atom (A) by 0.05 Å, after which the geometry was optimized by constraining the H–A distance, and the energy was calculated. These procedures were repeated until the H atom reached the A atom. The H-bond acceptor moiety was defined at the point where the H–A distance was 1.00 Å. All atomic coordinates were fully relaxed in the QM region, whereas all atomic coordinates were fixed in the MM region. The energy difference between the [D–H … A] and [D … H–A] states [ΔE(donor … acceptor)] was converted into ΔpK_a(donor … acceptor) using Eq. 1 (see below).

Vibrational frequencies were calculated using the same level of theory as the geometry optimizations based on the quantum-chemically optimized structures. The calculated frequencies were scaled by using a standard factor of 0.9614 for the B3LYP functional (56). ν_{O-D} was calculated by deuterating only the hydrogen atom of the H-bond donor moiety and avoiding mode coupling. $\nu_{C=O}$ was calculated for the COOH group (not for the deuterated COOD group).

RESULTS AND DISCUSSION

Estimation of ΔpK_a(donor … acceptor) in H-bonds

To calculate ΔpK_a(donor … acceptor) in the protein environments using a QM/MM approach, the potential-energy profiles for all 15 possible H-bond pairs from 6 alcohol/phenol molecules (Table 1) are analyzed. In all H-bond pairs, the H-bond donor is –OH. The energy difference between the [O_{donor}–H … $^{-}O_{acceptor}$] and [O_{donor}^{-} … H–$O_{acceptor}$] states [ΔE(donor … acceptor)] obtained from the potential-energy profile is highly correlated with the experimentally measured ΔpK_a(donor … acceptor) at 298 K for the H-bond pair, as suggested previously (15–17,57) (Fig. 2; Table 1). The correlation is best described by the following equation (coefficient of determination $R^2 = 0.99$):

$$\Delta pK_a(\text{donor ... acceptor}) = 0.39\ [\text{mol/kcal}]\ \Delta E(\text{donor ... acceptor}) \quad (1)$$

The accuracy of the ΔpK_a predictions is demonstrated in Fig. 2, showing a deviation of only 0.26 pK_a units. This approach has also been successfully employed and validated in a previous study (57). pK_a is linked to the reaction Gibbs free energy for deprotonation with a factor of $1/(RT\ln 10)$ (= 0.73 mol/kcal at 298 K). As ΔE(donor … acceptor) in Eq. 1 was calculated in vacuum, the difference between the factor of 0.73 in $1/(RT\ln 10)$ and the factor of 0.39 in Eq. 1 is primarily attributed to the insufficient consideration of the solvent reorganization in the surrounding environment.

In the ground-state PYP, Glu46 donates an H-bond to *p*CA, forming a standard H-bond (33–39). pK_a(Glu46) = 8.6 and pK_a(*p*CA) = 5.4 (i.e., ΔpK_a(Glu46 … *p*CA) = 3.2) are reported for the ground-state PYP (33). In the present study, ΔE(Glu46 … *p*CA) = 10.2 kcal/mol deduced from the potential-energy profile for the Glu46 … *p*CA H-bond leads to ΔpK_a(Glu46 … *p*CA) = 4.0 using Eq. 1, which is comparable with the reported value. These observations validate the reliability and consistency of the present approach. Below, ΔpK_a(donor … acceptor) is calculated by

TABLE 1 Isolated 15 H-bond pairs

H-bond donor		H-bond acceptor		
	pK_a[a]		pK_a[a]	ΔpK_a
methanol	15.5	2-propyn-1-ol	13.6	1.9
		phenol	9.99	5.5
		4-chlorophenol	9.41	6.1
		4-cyanophenol	7.97	7.5
		4-nitrophenol	7.15	8.4
2-propyn-1-ol	13.6	phenol	9.99	3.6
		4-chlorophenol	9.41	4.2
		4-cyanophenol	7.97	5.6
		4-nitrophenol	7.15	6.5
phenol	9.99	4-chlorophenol	9.41	0.58
		4-cyanophenol	7.97	2.0
		4-nitrophenol	7.15	2.8
4-chlorophenol	9.41	4-cyanophenol	7.97	1.4
		4-nitrophenol	7.15	2.3
4-cyanophenol	7.97	4-nitrophenol	7.15	0.82

[a]Ref. (58).

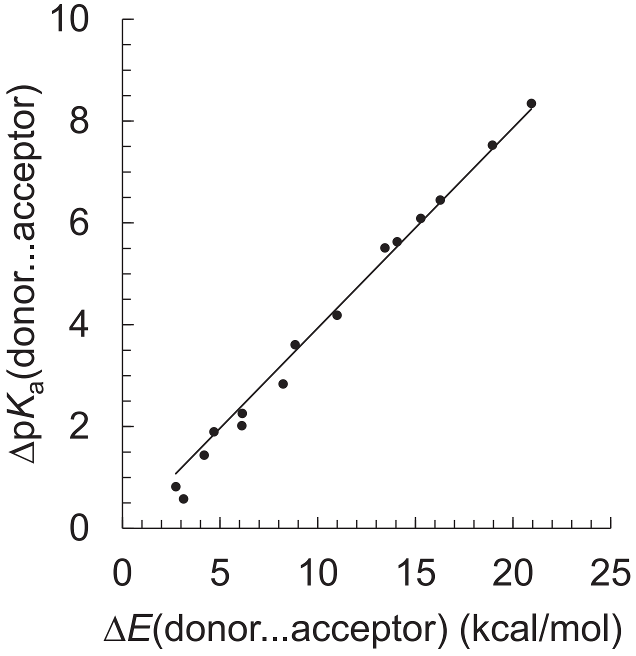

FIGURE 2 ΔpK_a(donor … acceptor) and ΔE(donor … acceptor) obtained from 15 H-bond pairs. See Table 1 for the H-bond donor/acceptor groups.

analyzing the potential-energy profile for the H-bond and using Eq. 1.

$\nu_{O\text{–}D}$(donor) and ΔpK_a(donor … acceptor) in H-bonds

To understand the relationship between $\nu_{O\text{–}D}$(donor) and ΔpK_a(donor … acceptor), the influence of the residue on $\nu_{O\text{–}D}$(donor) and ΔpK_a(donor … acceptor) is analyzed using the following three H-bonds in the protein crystal structures.

1) In the ground-state BR, ionized Asp85 accepts an H-bond from the adjacent water molecule (W402). Thus, $\nu_{O\text{–}D}$(W402) and ΔpK_a(DO_{W402}–D … $^{-}$OOC-Asp85) are calculated for the H-bond between deprotonated Asp85 and W402 in the ground-state structure (Fig. 3 *a*). In addition, the contributions of all BR residues (227 residues, excluding Asp85) to $\nu_{O\text{–}D}$(W402) and ΔpK_a(DO_{W402}–D … $^{-}$OOC-Asp85) are also calculated (Fig. 4 *a*).
2) In contrast, protonated Asp85 donates an H-bond to W401 in the N′ state. Thus, $\nu_{O\text{–}D}$(Asp85) and ΔpK_a(Asp85-COOD … $O_{W401}D_2$) are calculated for the H-bond between protonated Asp85 and W401 in the N′-state structure (Fig. 3 *a*). In addition, the contributions of all BR residues (226 residues, excluding Asp85) to $\nu_{O\text{–}D}$(Asp85) and ΔpK_a(Asp85-COOD … $O_{W401}D_2$) are also calculated (Fig. 4 *b*).
3) In the ground-state PYP, protonated Glu46 donates an H-bond to deprotonated *p*CA. Thus, $\nu_{O\text{–}D}$(Glu46) and ΔpK_a(Glu46-COOD … $^{-}$O-*p*CA) are calculated for the H-bond between protonated Glu46 and deprotonated *p*CA in the ground-state PYP (Fig. 3 *a*). In addition, the contributions of all PYP residues (124 residues, excluding Glu46) to $\nu_{O\text{–}D}$(Glu46) and ΔpK_a(Glu46-COOD … $^{-}$O-*p*CA) are also calculated (Fig. 4 *c*).

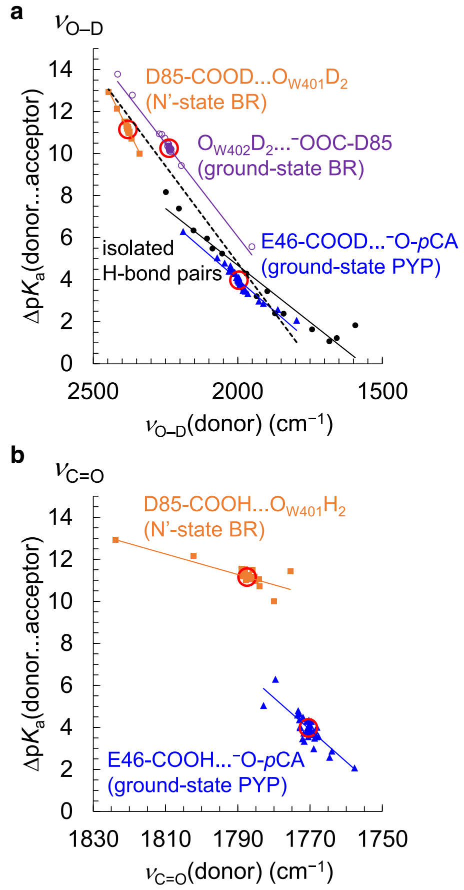

FIGURE 3 $\nu_{O\text{–}D}$(donor)/$\nu_{C=O}$(donor) and ΔpK_a in the protein environments obtained from the contribution of each residue. (*a*) $\nu_{O\text{–}D}$(donor) and ΔpK_a(donor … acceptor) in the ground-state BR (*purple open circles*, $R^2 = 0.99$), the N′-state BR (*orange squares*, $R^2 = 0.97$), the ground-state PYP (*blue triangles*, $R^2 = 0.93$), and isolated 15 H-bond pairs listed in Table 1 (*black closed circles*, $R^2 = 0.93$). The dashed black line indicates the fitting line for all data points of the ground-state BR, the N′-state BR, and the ground-state PYP ($R^2 = 0.90$). (*b*) $\nu_{C=O}$(donor) and ΔpK_a(donor … acceptor) in the N′-state BR (*orange squares*, $R^2 = 0.66$) and the ground-state PYP (*blue triangles*, $R^2 = 0.70$). The values in the original (not charge-depleted) structures are red circled. See Figs. S2–S4 for the details of each data point. To see this figure in color, go online.

Remarkably, $\nu_{O\text{–}D}$(donor) is correlated with ΔpK_a(donor … acceptor) among the three protein crystal structures, using the following equation (the *dashed black line* in Fig. 3 *a*):

$$\Delta pK_a(\text{donor ... acceptor}) \;=\; 0.019\,[\text{cm}]\,\nu_{O-D}(\text{donor}) - 32 \tag{2}$$

Eq. 2 can also reproduce $\nu_{O\text{–}D}$(donor) and ΔpK_a(donor … acceptor) for the 15 H-bond pairs listed in Table 1,

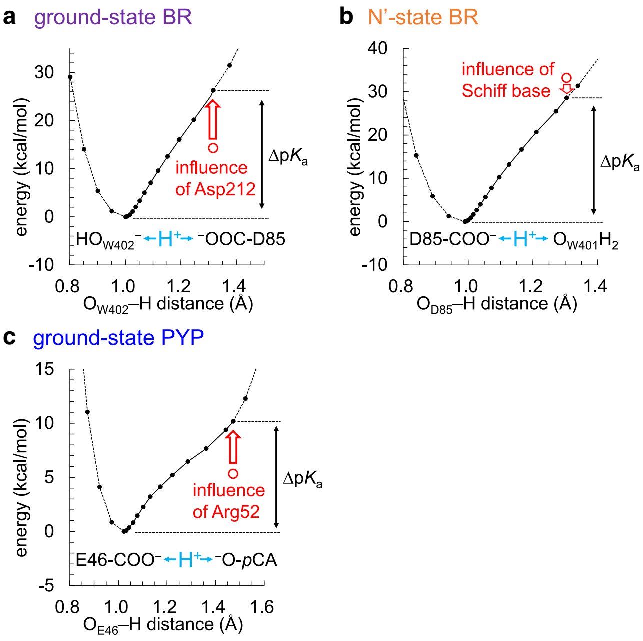

FIGURE 4 Potential energy profiles for the H-bonds. (*a*) Ground-state BR. (*b*) N′-state BR. (*c*) Ground-state PYP. Red open circles indicate the energy calculated in the absence of the electrostatic influence of the representative group (Asp212 in the ground-state BR, retinal Schiff base in the N′-state BR, and Arg52 in the ground-state PYP). Red arrows indicate the electrostatic contribution of the representative group to ΔpK_a(donor … acceptor). To see this figure in color, go online.

even in the absence of the protein environment (Fig. 3 *a*). These results suggest that ΔpK_a(donor … acceptor) is predominantly determined by $\nu_{O\text{–}D}$(donor) irrespective of the presence (BR and PYP) and absence of the protein environment.

The curvature at the H-bond donor moiety in the H-bond potential energy curve represents the spring constant k of the O–D harmonic oscillator (Fig. 5). The harmonic oscillator frequency, $\nu_{O\text{–}D}$, is proportional to the square-root of the spring constant ($\nu_{O\text{–}D} \propto k^{1/2}$). As ΔpK_a decreases, k also decreases, thereby leading to a decrease in $\nu_{O\text{–}D}$ (Fig. 5). Consequently, there exists a positive correlation between $\nu_{O\text{–}D}$ and ΔpK_a.

ΔpK_a(donor … acceptor) is less correlated with the H-bond donor … acceptor distance than $\nu_{O\text{–}D}$(donor) (Fig. 6 *a*). This is consistent with the view that the characteristics of H-bonds, including low-barrier H-bonds (i.e., ΔpK_a(donor … acceptor) ≈ 0), cannot be judged by the distance but by the shape of the potential-energy profile for the H-bonds (17).

The H-bond distance is often correlated with $\nu_{O\text{–}D}$(donor). The correlation between H-bond distances and $\nu_{O\text{–}D}$(donor) observed for solid hydrates (59) is also observed for the ground-state BR (14). The present result demonstrates that $\nu_{O\text{–}D}$(donor) is more highly correlated with ΔpK_a(donor … acceptor) than with H-bond distances (Figs. 3 *a* and 6 *b*).

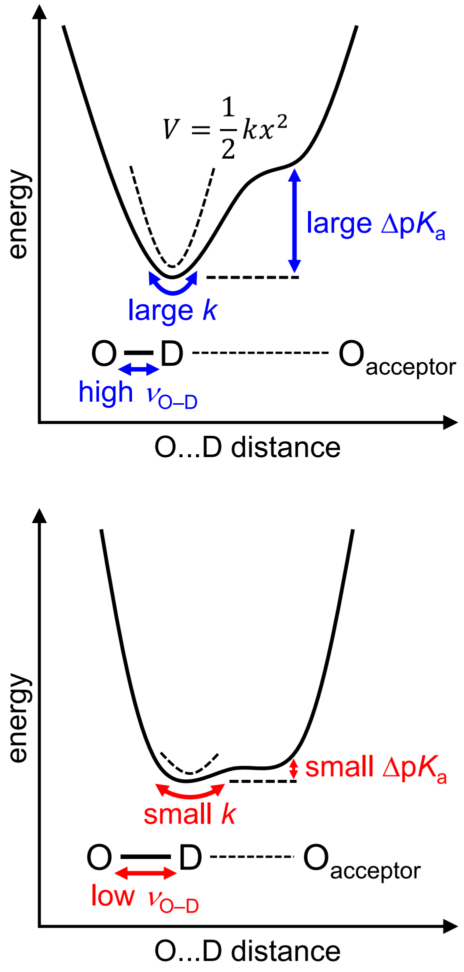

FIGURE 5 Relationship between $\nu_{O\text{–}D}$(donor) and ΔpK_a(donor … acceptor). The dotted curve indicates the fitting of the H-bond potential energy curve using a harmonic oscillator potential, $V = kx^2/2$, where k is the spring constant and x is the O–D bond distance. To see this figure in color, go online.

$\nu_{C=O}$(donor) and ΔpK_a(donor … acceptor) in H-bonds

$\nu_{C=O}$ for protonated carboxylate (1710–1770 cm^{-1}) is specifically high with respect to deprotonated carboxylate (~1400 and ~1570 cm^{-1}) (21). The influence of the residue on $\nu_{C=O}$(donor) and ΔpK_a(donor … acceptor) is analyzed for the H-bond between protonated Asp85 and W401 in the N′-state BR and the H-bond between protonated Glu46 and deprotonated *p*CA in the ground-state PYP. $\nu_{C=O}$(Asp85) is weakly associated with ΔpK_a(Asp85-COOH … $O_{W401}H_2$) in the N′ state structure of BR (Fig. 3 *b*). $\nu_{C=O}$(Glu46) is also weakly associated with ΔpK_a(Glu46-COOH … $^{-}$O-*p*CA) in the ground-state structure of PYP (Fig. 3 *b*).

The small ΔpK_a(donor … acceptor) leads to a decrease in $\nu_{O\text{–}H}$(donor) (i.e., the weakened O–H bond strength) (Fig. 3 *a*). Consequently, the resonance effect between the C=O and C–O characters is pronounced, causing a decrease in

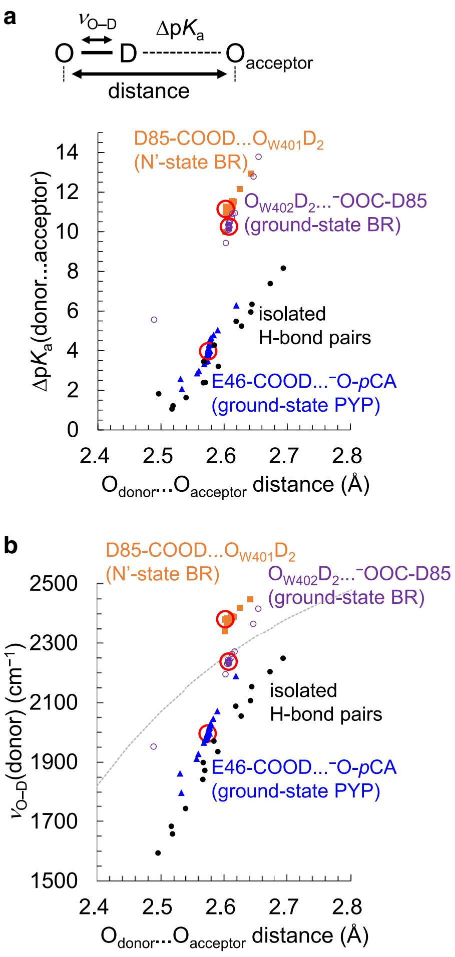

FIGURE 6 H-bond characteristics and the donor and acceptor (O_{donor} … $O_{acceptor}$) distances. (*a*) ΔpK_a(donor … acceptor) and O_{donor} … $O_{acceptor}$ distances: the ground-state BR (*purple open circles*), the N′-state BR (*orange squares*), the ground-state PYP (*blue triangles*), and isolated 15 H-bond pairs listed in Table 1 (*black closed circles*). (*b*) $\nu_{O\text{–}D}$(donor) and O_{donor} … $O_{acceptor}$ distances. The gray dotted curve indicates the correlation deduced from solid hydrates (59). The values in the original (not charge-depleted) structures are red circled. To see this figure in color, go online.

the double-bond character of the C=O bond, resulting in a decrease in $\nu_{C=O}$(donor) (Figs. 7 and S5 *a*). Indeed, a decrease in $\nu_{O\text{–}D}$(donor) (i.e., an elongated O–D distance) results in a shorter C–O distance (i.e., pronounced double-bond character) and a longer C=O distance (i.e., pronounced single-bond character) (Fig. S5, *b* and *c*). Note that the O–D and C=O bond distances are highly correlated with $\nu_{O\text{–}D}$(donor) and $\nu_{C=O}$(donor), respectively (Fig. S6). Thus, a positive correlation exists between $\nu_{C=O}$(donor) and ΔpK_a(donor … acceptor) (Fig. 7).

However, the correlation between $\nu_{C=O}$(Asp85) and ΔpK_a(Asp85-COOH … $O_{W401}H_2$) in the N′-state BR is not consistent with the correlation between $\nu_{C=O}$(Glu46) and ΔpK_a(Glu46-COOH … ⁻O-*p*CA) in the ground-state PYP (Fig. 3 *b*), even though the correlation between $\nu_{O\text{–}D}$(Asp85) and ΔpK_a(Asp85-COOD … $O_{W401}D_2$) is

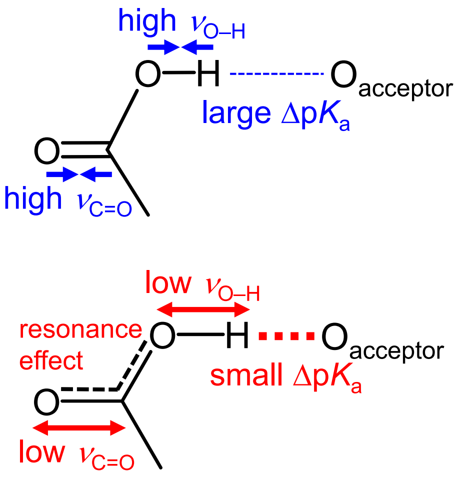

FIGURE 7 Relationship among $\nu_{C=O}$(donor), $\nu_{O\text{–}H}$(donor), and ΔpK_a (donor … acceptor). To see this figure in color, go online.

consistent with the correlation between $\nu_{O\text{–}D}$(Glu46) and ΔpK_a(Glu46-COOD … ⁻O-*p*CA) (Fig. 3 *a*). The weaker correlation between $\nu_{C=O}$(donor) and ΔpK_a(donor … acceptor) is due to the following factors: 1) O–D for $\nu_{O\text{–}D}$(donor) directly donates an H-bond to the acceptor group, whereas the C=O bond for $\nu_{C=O}$(donor) does not directly contain a proton. Nevertheless, the $\nu_{C=O}$(donor) increases/decreases as the $\nu_{O\text{–}D}$(donor) increases/decreases (Fig. S5) (22). 2) $\nu_{C=O}$(donor) may additionally be shifted due to an H-bond interaction with an adjacent group or electrostatic interactions with polar and charged groups (25,60). Thus, the correlation between $\nu_{C=O}$(donor) and ΔpK_a(donor … acceptor) is specific to the local protein environment.

Residues that decrease $\nu_{O\text{–}D}$(W402) and migrate the proton toward Asp85 in the ground-state BR

W402 donates H-bonds to Asp85 and Asp212 near the Schiff base (Fig. 8 *a*). pK_a(Asp212) is lower than pK_a(Asp85), as 1) Tyr57 and Tyr185 donate H-bonds to Asp212 and 2) Arg82 decreases pK_a(Asp212) more significantly than pK_a(Asp85) (14). Thus, ΔpK_a(DO_{W402}–D … ⁻OOC-Asp85) is smaller than ΔpK_a(DO_{W402}–D … ⁻OOC-Asp212) (i.e., the release of the proton toward Asp212 is more energetically uphill than that toward Asp85) and $\nu_{O\text{–}D}$(O_{W402}–D … Asp85) (2171 cm^{-1} (9,10)) is significantly lower than $\nu_{O\text{–}D}$(O_{W402}–D … Asp212) (2636 cm^{-1} (9)). This is why the H-bond between W402 and Asp85 has the lowest $\nu_{O\text{–}D}$(water) in BR (9,10,13,14), the likely origin of the proton-pumping activity. The present QM/MM calculation indicates that the positively charged retinal Schiff base, the H-bond donor to W402, contributes to the decrease in pK_a(W402) with respect to pK_a(Asp85) and migration of the proton toward Asp85 most significantly among all sites in the ground-state BR (Tables 2 and S3). Arg82 and Tyr57, which decrease pK_a(Asp212) with respect to pK_a(Asp85), also contribute to migration of the W402

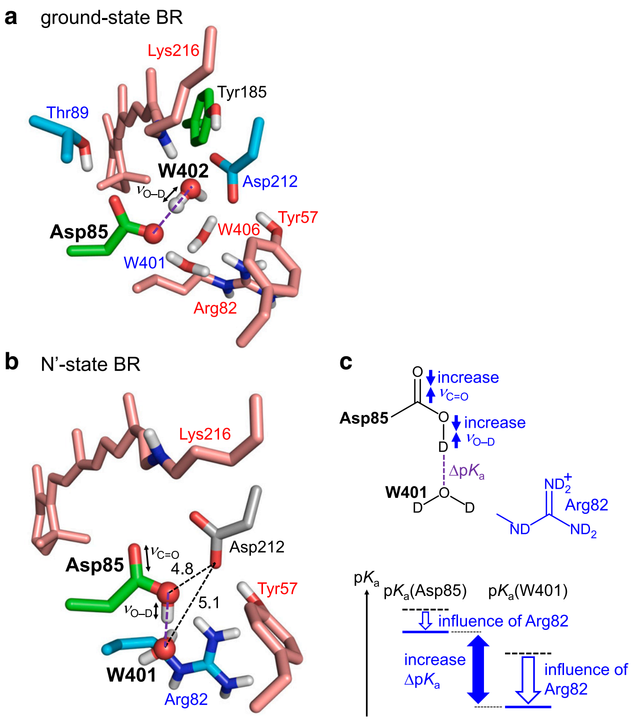

FIGURE 8 Key residues that influence $\nu_{O\text{–}D}$ and $\nu_{C=O}$ in BR. (*a*) QM/MM-optimized structure of the ground-state BR. Residues and water molecules that decrease and increase $\nu_{O\text{–}D}$(W402) are red and blue labeled, respectively. (*b*) QM/MM-optimized structure of the N′-state BR. Residues that decrease and increase $\nu_{O\text{–}D}$(Asp85) are red and blue labeled, respectively. Values indicate distances (Å). (*c*) Schematic illustration of the influence of Arg82: (*top*) on $\nu_{O\text{–}D}$(Asp85) and $\nu_{C=O}$(Asp85); (*bottom*) on ΔpK_a(Asp85-COOD … $O_{W401}D_2$) in the N′-state BR. To see this figure in color, go online.

proton toward Asp85 and the decrease in $\nu_{O\text{–}D}$(O_{W402}–D … Asp85) (Table 2).

Thr89 increases $\nu_{O\text{–}D}$(O_{W402}–D … Asp85), as it donates an H-bond to Asp85 and inhibits migration of the W402 proton toward Asp85 (Table 2). Indeed, $\nu_{O\text{–}D}$(W402) decreases by ~80 cm^{-1} upon mutation of Thr89 to alanine (10), which is consistent with the present result.

Protonated Asp85 in the N′-state BR

Asp85 is protonated in the N′-state BR (22,61), which is a prerequisite for the subsequent release of the proton toward the extracellular side. Although Arg82 decreases pK_a(Asp85), Arg82 is the residue that contributes most to protonation of Asp85 among all sites in the N′-state BR, as suggested by the increase in $\nu_{O\text{–}D}$(Asp85) (Tables 3 and S4) and $\nu_{C=O}$(Asp85) (Table 4). In the N′-state BR, Arg82 is closer to W401 (6.0 Å) than Asp85 (7.8 Å) (Fig. 8 *b*). Arg82 decreases pK_a(W401) more significantly than pK_a(Asp85) and increases ΔpK_a(Asp85-COOD … O_{W401} D_2) (Fig. 8 *c*). Eventually, Arg82 contributes to the fixation of the proton at the H-bond donor (Asp85) moiety, increasing $\nu_{O\text{–}D}$(Asp85) and $\nu_{C=O}$(Asp85) (Tables 3 and 4).

TABLE 2 Residues and water molecules that decrease/increase $\nu_{O\text{–}D}$(W402) (cm^{-1}) and ΔpK_a(DO_{W402}–D … $^{-}$OOC-Asp85) most significantly in the ground-state BR

Decreasing $\nu_{O\text{–}D}$(W402)			Increasing $\nu_{O\text{–}D}$(W402)		
Proton transfer (PT) toward Asp85	$\Delta\nu_{O\text{–}D}$	$\Delta\Delta pK_a$	PT toward W402	$\Delta\nu_{O\text{–}D}$	$\Delta\Delta pK_a$
Retinal Schiff base	−178	−3.5	Asp212	286	4.7
Arg82	−34	−0.7	H_2O-401	55	1.0
Tyr57	−26	−0.7	Thr89	42	0.8
H_2O-406	−17	−0.5			

In both the ground and N′-state BRs, the Schiff base is closer to the H-bond donor (W402 in the ground-state BR/Asp85 in the N′-state BR) than the acceptor (Asp85 in the ground-state BR/W401 in the N′-state BR) (Fig. 8, *a* and *b*). The Schiff base decreases pK_a(donor) more significantly than pK_a(acceptor) and decreases ΔpK_a(donor … acceptor). Thus, the Schiff base contributes to the migration of the proton from the H-bond donor toward the acceptor, decreasing $\nu_{O\text{–}D}$(W402) in the ground-state BR (Table 2) and $\nu_{O\text{–}D}$(Asp85) and $\nu_{C=O}$(Asp85) in the N′-state BR (Tables 3 and 4).

As Asp212 is equidistant from Asp85 (4.8 Å) and W401 (5.1 Å) in the N′-state BR (Fig. 8 *b*), the increase in pK_a(Asp85) caused by Asp212 is the same as the increase in pK_a(W401) caused by Asp212. Thus, Asp212 does not significantly contribute to ΔpK_a(Asp85-COOD … $O_{W401}D_2$) (−0.3) and $\nu_{O\text{–}D}$(Asp85) (−4 cm^{-1}) irrespective of a negatively charged residue (Table 3).

However, Asp212 increases $\nu_{C=O}$(Asp85) by 12 cm^{-1} (Table 4), because the electrostatic interaction between Asp212 and Asp85 specifically decreases the C=O distance in Asp85 (Fig. 8 *b*). The present case indicates that C=O distances are additionally affected by the local protein environment, which is a reason for the weak correlation between $\nu_{C=O}$(donor) and ΔpK_a(donor … acceptor).

Protonated Glu46 in the ground-state PYP

Protonated Glu46 is a prerequisite for the formation of a stable H-bond with deprotonated *p*CA in the ground-state PYP (33–39). The present result indicates that the protonated state of Arg52 (33) and the H-bond donation from Tyr42

TABLE 3 Residues that decrease/increase $\nu_{O\text{–}D}$(Asp85) (cm^{-1}) and ΔpK_a(Asp85-COOD … $O_{W401}D_2$) most significantly in the N′-state BR

Decreasing $\nu_{O\text{–}D}$(Asp85)			Increasing $\nu_{O\text{–}D}$(Asp85)		
PT toward W401	$\Delta\nu_{O\text{–}D}$	$\Delta\Delta pK_a$	PT toward Asp85	$\Delta\nu_{O\text{–}D}$	$\Delta\Delta pK_a$
Retinal Schiff base	−67	−1.8	Arg82	39	1.2
Tyr57	−12	−0.4	Arg7	12	0.4
Glu194	−12	−0.4			
Asp212	−4	−0.3			

TABLE 4 Residues that decrease/increase $\nu_{C=O}$(Asp85) (cm^{-1}) and ΔpK_a(Asp85-COOH … $O_{W401}H_2$) most significantly in the N′-state BR

Decreasing $\nu_{C=O}$(Asp85)			Increasing $\nu_{C=O}$(Asp85)		
PT toward W401	$\Delta\nu_{C=O}$	$\Delta\Delta pK_a$	PT toward Asp85	$\Delta\nu_{C=O}$	$\Delta\Delta pK_a$
Retinal Schiff base	−36	−1.8	Asp212	12	−0.3
			Arg82	7	1.2
			Arg7	3	0.4

TABLE 6 Residues that decrease/increase $\nu_{C=O}$(Glu46) (cm^{-1}) and ΔpK_a(Glu46-COOH … $^{-}$O-*p*CA) most significantly in the ground-state PYP

Decreasing $\nu_{C=O}$(Glu46)			Increasing $\nu_{C=O}$(Glu46)		
PT toward *p*CA	$\Delta\nu_{C=O}$	$\Delta\Delta pK_a$	PT toward Glu46	$\Delta\nu_{C=O}$	$\Delta\Delta pK_a$
Arg124	−12	−1.1	Arg52	13	1.9
Asp97	−9	−2.3	Asp48	6	1.1
Lys123	−3	−0.6	Tyr42	6	1.4

to *p*CA contribute most to protonation of Glu46 in the ground-state PYP (Tables 5, 6, and S5). In particular, Tyr42 contributes to an increase in $\nu_{C=O}$(Glu46) of 6 cm^{-1} in the ground-state PYP (Table 6). Consistently, FTIR studies suggested that $\nu_{C=O}$(Glu46) decreases by 12 cm^{-1} upon mutation of Tyr42 to phenylalanine (62).

While estimating vibrational frequencies solely from the "pK_a" (of the H-bond donor or acceptor moiety) may be adequate as an initial approach in some cases (e.g., (9,63)), the consideration of "ΔpK_a" (of the H-bond donor and acceptor moieties) is required for a more comprehensive and detailed discussion as presented in this study. Importantly, the shifts in $\nu_{O–D}$(donor) and $\nu_{C=O}$(donor) caused by an external charged group does not merely originate from the shift in pK_a(donor) alone, but from the total shift in ΔpK_a(donor … acceptor). This is because the shifts in $\nu_{O–D}$(donor) and $\nu_{C=O}$(donor) originate not only from the electrostatic interaction between the charged group and the H-bond donor moiety but also from the electrostatic interaction between the charged group and the H-bond acceptor moiety. Thus, the position of the charged group with respect to both the H-bond donor and acceptor groups are crucial to the resulting shifts in these properties (Fig. 9). Positively charged basic residues commonly decrease pK_a(Glu46). However, Lys123 and Arg124 decrease ΔpK_a(Glu46 … *p*CA), whereas Arg52 increases ΔpK_a(Glu46 … *p*CA) (Tables 5 and 6). Lys123 and Arg124 are closer to Glu46 than *p*CA (Fig. S7). Lys123 and Arg124 decrease pK_a(Glu46) more significantly than pK_a(*p*CA), which decreases ΔpK_a(Glu46 … *p*CA), $\nu_{O–D}$(Glu46), and $\nu_{C=O}$(Glu46) (Fig. 9 *a*; Tables 5 and 6). On the other hand, Arg52 is closer to *p*CA than Glu46 (Fig. S7). Arg52 decreases pK_a(*p*CA) more significantly than pK_a(Glu46), which increases ΔpK_a(Glu46 … *p*CA), $\nu_{O–D}$(Glu46), and $\nu_{C=O}$(Glu46) (Fig. 9 *b*; Tables 5 and 6). Thus, while Arg52 decreases pK_a(Glu46), it conversely increases ΔpK_a(Glu46 … *p*CA), leading to the fixation of proton at the Glu46 moiety and consequently increasing $\nu_{O–D}$(Glu46) and $\nu_{C=O}$(Glu46).

TABLE 5 Residues that decrease/increase $\nu_{O–D}$(Glu46) (cm^{-1}) and ΔpK_a(Glu46-COOD … $^{-}$O-*p*CA) most significantly in the ground-state PYP

Decreasing $\nu_{O–D}$(Glu46)			Increasing $\nu_{O–D}$(Glu46)		
PT toward *p*CA	$\Delta\nu_{O–D}$	$\Delta\Delta pK_a$	PT toward Glu46	$\Delta\nu_{O–D}$	$\Delta\Delta pK_a$
Asp97	−193	−2.3	Arg52	199	1.9
Arg124	−75	−1.1	Tyr42	134	1.4
Asp71	−50	−0.8	Asp48	84	1.1

The contribution of the acidic residue to ΔpK_a(Glu46 … *p*CA), $\nu_{O–D}$(Glu46), and $\nu_{C=O}$(Glu46) also depends on the position of the acidic residue (Fig. 9). Negatively charged acidic residues commonly increase pK_a(Glu46). Asp48 is closer to Glu46 than *p*CA (Fig. S7). Asp48 increases pK_a(Glu46) more significantly than pK_a(*p*CA), which increases ΔpK_a(Glu46 … *p*CA), $\nu_{O–D}$(Glu46), and $\nu_{C=O}$(Glu46) (Fig. 9 *c*; Tables 5 and 6). On the other hand, Asp71 and Asp97 are closer to *p*CA than Glu46 (Fig. S7). Asp71 and Asp97 increase pK_a(*p*CA) more significantly than pK_a(Glu46), which decreases ΔpK_a(Glu46 … *p*CA), $\nu_{O–D}$(Glu46), and $\nu_{C=O}$(Glu46) (Fig. 9 *d*; Tables 5 and 6). Thus, while Asp71 and Asp97 increase pK_a(Glu46), they conversely decrease ΔpK_a(Glu46 … *p*CA), leading to the proton release toward the *p*CA moiety and consequently decreasing $\nu_{O–D}$(Glu46) and $\nu_{C=O}$(Glu46).

CONCLUSIONS

The O–D stretching vibrational frequency of the H-bond donor [$\nu_{O–D}$(donor)] is correlated with the pK_a difference between the H-bond donor and acceptor [ΔpK_a(donor … acceptor)] in different protein environments, as well as in the absence of a protein environment (Fig. 3 *a*). This correlation suggests that $\nu_{O–D}$(donor) can be a useful tool for estimating ΔpK_a(donor … acceptor) in proteins (Eq. 2). On the other hand, the correlation between $\nu_{C=O}$(donor) and ΔpK_a(donor … acceptor) is weaker, mainly due to the fact that the C=O bond does not directly contain a proton (Fig. 3 *b*). However, the shift in $\nu_{C=O}$(donor) may still be used to estimate the shift in ΔpK_a(donor … acceptor), especially when comparing similar protein environments (e.g., comparison between reaction intermediate states in the same protein). It should be noted that the location of a charged/polar residue with respect to the H-bond donor and acceptor groups also plays a crucial role in the direction of the shifts in $\nu_{O–D}$(donor), $\nu_{C=O}$(donor), and ΔpK_a(donor … acceptor) (Figs. 8 and 9). For instance, a basic residue near the H-bond acceptor group would decrease pK_a(acceptor) more significantly than pK_a(donor), leading to an increase

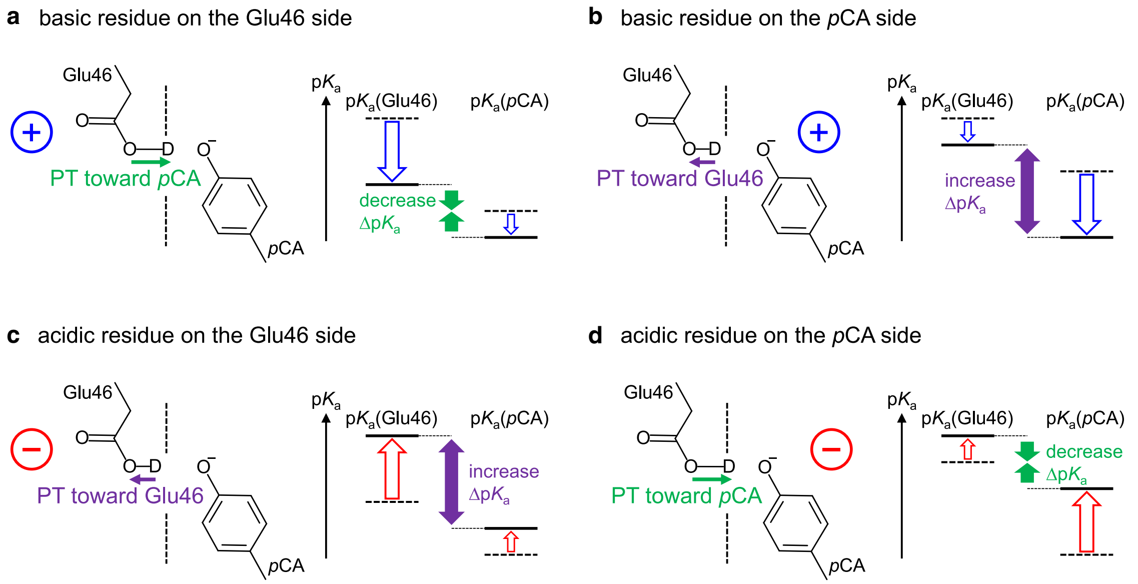

FIGURE 9 Electrostatic influence and shift in ΔpK_a(Glu46 … *p*CA) in the ground-state PYP. (*a*) Decrease in ΔpK_a(Glu46 … *p*CA) due to the influence of a basic residue on the Glu46 side. (*b*) Increase in ΔpK_a(Glu46 … *p*CA) due to the influence of a basic residue on the *p*CA side. The blue open arrows indicate the influence of a basic residue on pK_a(Glu46) and pK_a(*p*CA). (*c*) Increase in ΔpK_a(Glu46 … *p*CA) due to the influence of an acidic residue on the Glu46 side. (*d*) Decrease in ΔpK_a(Glu46 … *p*CA) due to the influence of an acidic residue on the *p*CA side. The red open arrows indicate the influence of an acidic residue on pK_a(Glu46) and pK_a(*p*CA). The green and purple closed arrows indicate the decrease and increase in ΔpK_a(Glu46 … *p*CA) due to the electrostatic influence of the residue, respectively. To see this figure in color, go online.

in ΔpK_a(donor … acceptor) and ν_{O-D}(donor)/$\nu_{C=O}$(donor). Conversely, an acidic residue near the H-bond acceptor group would increase pK_a(acceptor) more significantly than pK_a(donor), resulting in a decrease in ΔpK_a(donor … acceptor) and ν_{O-D}(donor)/$\nu_{C=O}$(donor). These results provide insights into the factors that contribute to proton transfer in protein environments and suggest that experimentally measured ν_{O-D}(donor) and $\nu_{C=O}$(donor) values can be used to estimate ΔpK_a(donor … acceptor), and provides a useful framework for understanding and investigating proton transfer events in proteins.

SUPPORTING MATERIAL

Supporting material can be found online at https://doi.org/10.1016/j.bpj.2023.10.012.

AUTHOR CONTRIBUTIONS

H.I. designed the research. M.T., K.S., and H.I performed the research. M.T., K.S., and H.I. analyzed data. M.T. and H.I. wrote the paper.

ACKNOWLEDGMENTS

This research was supported by JSPS KAKENHI (JP20H03217 and JP23H02444 to H.I., JP23H04963 to K.S., and JP22KJ1109 to M.T.), and the Interdisciplinary Computational Science Program in CCS, University of Tsukuba.

DECLARATION OF INTERESTS

The authors declare no competing interests.

REFERENCES

1. Oesterhelt, D., and W. Stoeckenius. 1971. Rhodopsin-like protein from the purple membrane of *Halobacterium halobium*. *Nat. New Biol.* 233:149–152.
2. Luecke, H., B. Schobert, …, J. K. Lanyi. 1999. Structure of bacteriorhodopsin at 1.55 Å resolution. *J. Mol. Biol.* 291:899–911.
3. Kandori, H., Y. Yamazaki, …, A. Maeda. 1995. Water-mediated proton-transfer in proteins: an FTIR study of bacteriorhodopsin. *J. Am. Chem. Soc.* 117:2118–2119.
4. Maeda, A., J. Sasaki, …, J. K. Lanyi. 1994. Interaction of aspartate-85 with a water molecule and the protonated Schiff base in the L intermediate of bacteriorhodopsin: a Fourier-transform infrared spectroscopic study. *Biochemistry.* 33:1713–1717.
5. Ernst, O. P., D. T. Lodowski, …, H. Kandori. 2014. Microbial and animal rhodopsins: structures, functions, and molecular mechanisms. *Chem. Rev.* 114:126–163.
6. Engelhard, M., K. Gerwert, …, F. Siebert. 1985. Light-driven protonation changes of internal aspartic acids of bacteriorhodopsin: an investigation by static and time-resolved infrared difference spectroscopy using [4-^{13}C] aspartic acid labeled purple membrane. *Biochemistry.* 24:400–407.
7. Kandori, H. 2004. Hydration switch model for the proton transfer in the Schiff base region of bacteriorhodopsin. *Biochim. Biophys. Acta.* 1658:72–79.
8. Benedict, W. S., N. Gailar, and E. K. Plyler. 1956. Rotation-vibration spectra of deuterated water vapor. *J. Chem. Phys.* 24:1139–1165.
9. Shibata, M., and H. Kandori. 2005. FTIR studies of internal water molecules in the Schiff base region of bacteriorhodopsin. *Biochemistry.* 44:7406–7413.

10. Shibata, M., T. Tanimoto, and H. Kandori. 2003. Water molecules in the Schiff base region of bacteriorhodopsin. *J. Am. Chem. Soc.* 125:13312–13313.
11. Kandori, H. 2020. Structure/function study of photoreceptive proteins by FTIR spectroscopy. *Bull. Chem. Soc. Jpn.* 93:904–926.
12. Muroda, K., K. Nakashima, …, H. Kandori. 2012. Protein-bound water as the determinant of asymmetric functional conversion between light-driven proton and chloride pumps. *Biochemistry.* 51:4677–4684.
13. Hayashi, S., and I. Ohmine. 2000. Proton transfer in bacteriorhodopsin: structure, excitation, IR spectra, and potential energy surface analyses by an *ab initio* QM/MM method. *J. Phys. Chem. B.* 104:10678–10691.
14. Saito, K., H. Kandori, and H. Ishikita. 2012. Factors that differentiate the H-bond strengths of water near the Schiff bases in bacteriorhodopsin and *Anabaena* sensory rhodopsin. *J. Biol. Chem.* 287:34009–34018.
15. Perrin, C. L., and J. B. Nielson. 1997. "Strong" hydrogen bonds in chemistry and biology. *Annu. Rev. Phys. Chem.* 48:511–544.
16. Ishikita, H., and K. Saito. 2014. Proton transfer reactions and hydrogen-bond networks in protein environments. *J. R. Soc. Interface.* 11, 20130518.
17. Schutz, C. N., and A. Warshel. 2004. The low barrier hydrogen bond (LBHB) proposal revisited: the case of the Asp...His pair in serine proteases. *Proteins.* 55:711–723.
18. Hasegawa, N., H. Jonotsuka, …, K. Takeda. 2018. X-ray structure analysis of bacteriorhodopsin at 1.3 Å resolution. *Sci. Rep.* 8, 13123.
19. Schobert, B., L. S. Brown, and J. K. Lanyi. 2003. Crystallographic structures of the M and N intermediates of bacteriorhodopsin: assembly of a hydrogen-bonded chain of water molecules between Asp-96 and the retinal Schiff base. *J. Mol. Biol.* 330:553–570.
20. Anderson, S., S. Crosson, and K. Moffat. 2004. Short hydrogen bonds in photoactive yellow protein. *Acta Crystallogr. D Biol. Crystallogr.* 60:1008–1016.
21. Barth, A. 2000. The infrared absorption of amino acid side chains. *Prog. Biophys. Mol. Biol.* 74:141–173.
22. Saito, K., T. Xu, and H. Ishikita. 2022. Correlation between C=O stretching vibrational frequency and pK_a shift of carboxylic acids. *J. Phys. Chem. B.* 126:4999–5006.
23. Braiman, M. S., O. Bousché, and K. J. Rothschild. 1991. Protein dynamics in the bacteriorhodopsin photocycle: submillisecond Fourier-transform infrared-spectra of the L-photointermediates, M-photointermediates, and N-photointermediates. *Proc. Natl. Acad. Sci. USA* 88:2388–2392.
24. Xie, A., W. D. Hoff, …, K. J. Hellingwerf. 1996. Glu46 donates a proton to the 4-hydroxycinnamate anion chromophore during the photocycle of photoactive yellow protein. *Biochemistry.* 35:14671–14678.
25. Takei, K., R. Takahashi, and T. Noguchi. 2008. Correlation between the hydrogen-bond structures and the C=O stretching frequencies of carboxylic acids as studied by density functional theory calculations: theoretical basis for interpretation of infrared bands of carboxylic groups in proteins. *J. Phys. Chem. B.* 112:6725–6731.
26. Meyer, T. E., E. Yakali, …, G. Tollin. 1987. Properties of a water-soluble, yellow protein isolated from a halophilic phototrophic bacterium that has photochemical activity analogous to sensory rhodopsin. *Biochemistry.* 26:418–423.
27. Meyer, T. E. 1985. Isolation and characterization of soluble cytochromes, ferredoxins and other chromophoric proteins from the halophilic phototrophic bacterium *Ectothiorhodospira halophila.* *Biochim. Biophys. Acta.* 806:175–183.
28. Sprenger, W. W., W. D. Hoff, …, K. J. Hellingwerf. 1993. The eubacterium *Ectothiorhodospira halophila* is negatively phototactic, with a wavelength dependence that fits the absorption spectrum of the photoactive yellow protein. *J. Bacteriol.* 175:3096–3104.
29. Hoff, W. D., P. Düx, …, K. J. Hellingwerf. 1994. Thiol ester-linked *p*-coumaric acid as a new photoactive prosthetic group in a protein with rhodopsin-like photochemistry. *Biochemistry.* 33:13959–13962.
30. Kort, R., H. Vonk, …, K. J. Hellingwerf. 1996. Evidence for *trans-cis* isomerization of the *p*-coumaric acid chromophore as the photochemical basis of the photocycle of photoactive yellow protein. *FEBS Lett.* 382:73–78.
31. Hellingwerf, K. J., J. Hendriks, and T. Gensch. 2003. Photoactive yellow protein, a new type of photoreceptor protein: Will this “Yellow Lab” bring us where we want to go? *J. Phys. Chem. A.* 107:1082–1094.
32. Hoff, W. D., I. H. van Stokkum, …, K. J. Hellingwerf. 1994. Measurement and global analysis of the absorbance changes in the photocycle of the photoactive yellow protein from *Ectothiorhodospira halophila.* *Biophys. J.* 67:1691–1705.
33. Saito, K., and H. Ishikita. 2012. Energetics of short hydrogen bonds in photoactive yellow protein. *Proc. Natl. Acad. Sci. USA* 109:167–172.
34. Saito, K., and H. Ishikita. 2012. H atom positions and nuclear magnetic resonance chemical shifts of short H bonds in photoactive yellow protein. *Biochemistry.* 51:1171–1177.
35. Saito, K., and H. Ishikita. 2013. Formation of an unusually short hydrogen bond in photoactive yellow protein. *Biochim. Biophys. Acta.* 1827:387–394.
36. Graen, T., L. Inhester, …, G. Groenhof. 2016. The low barrier hydrogen bond in the photoactive yellow protein: A vacuum artifact absent in the crystal and solution. *J. Am. Chem. Soc.* 138:16620–16631.
37. Thomson, B., J. Both, and S. G. Boxer. 2019. Perturbation of short hydrogen bonds in photoactive yellow protein via noncanonical amino acid incorporation. *J. Phys. Chem. B.* 123:4844–4849.
38. Yoshimura, Y., N. A. Oktaviani, …, F. A. A. Mulder. 2017. Unambiguous determination of protein arginine ionization states in solution by NMR spectroscopy. *Angew. Chem. Int. Ed.* 56:239–242.
39. Wang, J. 2019. Visualization of H atoms in the X-ray crystal structure of photoactive yellow protein: Does it contain low-barrier hydrogen bonds? *Protein Sci.* 28:1966–1972.
40. Tsujimura, M., H. Tamura, …, H. Ishikita. 2022. Absorption wavelength along chromophore low-barrier hydrogen bonds. *iScience.* 25, 104247.
41. (2012). Jaguar. version 7.9. Schrödinger, LLC.
42. Brooks, B. R., R. E. Bruccoleri, …, M. Karplus. 1983. CHARMM: a program for macromolecular energy, minimization, and dynamics calculations. *J. Comput. Chem.* 4:187–217.
43. MacKerell, A. D., D. Bashford, …, M. Karplus. 1998. All-atom empirical potential for molecular modeling and dynamics studies of proteins. *J. Phys. Chem. B.* 102:3586–3616.
44. Jo, S., T. Kim, …, W. Im. 2008. CHARMM-GUI: a web-based graphical user interface for CHARMM. *J. Comput. Chem.* 29:1859–1865.
45. Bayly, C. I., P. Cieplak, …, P. A. Kollman. 1993. A well-behaved electrostatic potential based method using charge restraints for deriving atomic charges: the RESP model. *J. Phys. Chem.* 97:10269–10280.
46. Rabenstein, B., G. M. Ullmann, and E. W. Knapp. 1998. Energetics of electron-transfer and protonation reactions of the quinones in the photosynthetic reaction center of *Rhodopseudomonas viridis.* *Biochemistry.* 37:2488–2495.
47. Rabenstein, B., G. M. Ullmann, and E.-W. Knapp. 1998. Calculation of protonation patterns in proteins with structural relaxation and molecular ensembles - application to the photosynthetic reaction center. *Eur. Biophys. J.* 27:626–637.
48. Bashford, D., and M. Karplus. 1990. pK_a's of ionizable groups in proteins: atomic detail from a continuum electrostatic model. *Biochemistry.* 29:10219–10225.
49. Nozaki, Y., and C. Tanford. 1967. Acid-base titrations in concentrated guanidine hydrochloride. Dissociation constants of the guanidinium ion and of some amino acids. *J. Am. Chem. Soc.* 89:736–742.
50. Tanokura, M. 1983. ^{1}H nuclear magnetic resonance titration curves and microenvironments of aromatic residues in bovine pancreatic ribonuclease A. *J. Biochem.* 94:51–62.
51. Tanokura, M. 1983. ^{1}H-NMR study on the tautomerism of the imidazole ring of histidine residues: I. Microscopic pK values and molar

ratios of tautomers in histidine-containing peptides. *Biochim. Biophys. Acta.* 742:576–585.

52. Tanokura, M. 1983. ^{1}H-NMR study on the tautomerism of the imidazole ring of histidine residues: II. Microenvironments of histidine-12 and histidine-119 of bovine pancreatic ribonuclease A. *Biochim. Biophys. Acta.* 742:586–596.

53. Rabenstein, B., and E.-W. Knapp. 2001. Calculated pH-dependent population and protonation of carbon-monoxy-myoglobin conformers. *Biophys. J.* 80:1141–1150.

54. (2012). QSite. Version 5.8. Schrödinger, LLC.

55. Jorgensen, W. L., D. S. Maxwell, and J. Tirado-Rives. 1996. J. Development and testing of the OPLS all-atom force field on conformational energetics and properties of organic liquids. *J. Am. Chem. Soc.* 118:11225–11236.

56. Scott, A. P., and L. Radom. 1996. Harmonic vibrational frequencies: an evaluation of Hartree-Fock, Møller-Plesset, quadratic configuration interaction, density functional theory, and semiempirical scale factors. *J. Phys. Chem.* 100:16502–16513.

57. Ikeda, T., K. Saito, …, H. Ishikita. 2017. The existence of an isolated hydronium ion in the interior of proteins. *Angew. Chem. Int. Ed.* 56:9151–9154.

58. (2022). CRC Handbook of Chemistry and Physics, 103rd edition. CRC press.

59. Mikenda, W. 1986. Stretching frequency versus bond distance correlation of O–D(H), Y (Y = N, O, S, Se, Cl, Br, I) hydrogen-bonds in solid hydrates. *J. Mol. Struct.* 147:1–15.

60. Nie, B., J. Stutzman, and A. Xie. 2005. A vibrational spectral maker for probing the hydrogen-bonding status of protonated Asp and Glu residues. *Biophys. J.* 88:2833–2847.

61. Dioumaev, A. K., L. S. Brown, …, J. K. Lanyi. 2001. Coupling of the reisomerization of the retinal, proton uptake, and reprotonation of Asp-96 in the N photointermediate of bacteriorhodopsin. *Biochemistry.* 40:11308–11317.

62. Joshi, C. P., H. Otto, …, M. P. Heyn. 2009. Strong hydrogen bond between glutamic acid 46 and chromophore leads to the intermediate spectral form and excited state proton transfer in the Y42F mutant of the photoreceptor photoactive yellow protein. *Biochemistry.* 48:9980–9993.

63. Nack, M., I. Radu, …, J. Heberle. 2010. The DC gate in Channelrhodopsin-2: crucial hydrogen bonding interaction between C128 and D156. *Photochem. Photobiol. Sci.* 9:194–198.

Biophysical Journal, Volume *122*

Supplemental information

Stretching vibrational frequencies and pK_a differences in H-bond networks of protein environments

Masaki Tsujimura, Keisuke Saito, and Hiroshi Ishikita

Supporting Information

Stretching vibrational frequencies and pK_a differences in H-bond networks of protein environments

Masaki Tsujimura [1*], Keisuke Saito [2,3], Hiroshi Ishikita [2,3*]

1) Department of Advanced Interdisciplinary Studies, The University of Tokyo, 4-6-1 Komaba, Meguro-ku, Tokyo 153-8904, Japan

2) Department of Applied Chemistry, The University of Tokyo, 7-3-1 Hongo, Bunkyo-ku, Tokyo 113-8654, Japan

3) Research Center for Advanced Science and Technology, The University of Tokyo, 4-6-1 Komaba, Meguro-ku, Tokyo 153-8904, Japan

Table S1. Residues with non-standard protonation states in the ground-state BR.

residue	protonation state
Asp96	protonated
Asp115	protonated
Glu194	protonated

Table S2. Residues with non-standard protonation states in the N'-state BR.

residue	protonation state
Asp85	protonated
Asp96	protonated
Asp115	protonated
Glu204	protonated

Table S3. Contributions of residues to $\Delta pK_a(DO_{W402}–D... ^{-}OOC\text{-}Asp85)$ in the ground-state BR calculated by using a QM/MM approach and by solving the Poisson-Boltzmann equation (PBE) [a].

**decreasing ΔpK_a** [PT toward *p*CA]	**QM/MM**	**PBE**	**increasing ΔpK_a** [PT toward Glu46]	**QM/MM**	**PBE**
retinal Schiff base	−3.5	−4.2	Asp212	4.7	5.7
Arg82	−0.7	−1.3	Thr89	0.8	1.2
Tyr57	−0.7	−0.7			

[a] PBE was solved using the crystal structure (instead of using the QM/MM-optimized structure). The pK_a value employed as a reference for H_2O/OH^- is 15.74. Atomic charges of OH^- are obtained from the CHARMM22 parameter set.

Table S4. Contributions of residues to ΔpK_a(Asp85-COOD...$O_{W401}D_2$) in the N'-state BR calculated by using a QM/MM approach and by solving PBE [a].

**decreasing ΔpK_a** [PT toward *p*CA]	**QM/MM**	**PBE**	**increasing ΔpK_a** [PT toward Glu46]	**QM/MM**	**PBE**
retinal Schiff base	−1.8	−1.7	Arg82	1.2	2.4
Tyr57	−0.4	−0.3	Arg7	0.4	0.2
Glu194	−0.4	−0.5			
Asp212	−0.3	−1.1			

[a] PBE was solved using the crystal structure (instead of using the QM/MM-optimized structure). The pK_a value employed as a reference for H_3O^+/H_2O is −1.74. Atomic charges of H_3O^+ are obtained from Ref. [1].

Table S5. Contributions of residues to ΔpK_a(Glu46-COOD...$^{-}$O-*p*CA) in the ground-state PYP calculated by using a QM/MM approach and by solving PBE [a].

**decreasing ΔpK_a** [PT toward *p*CA]	**QM/MM**	**PBE**	**increasing ΔpK_a** [PT toward Glu46]	**QM/MM**	**PBE**
Asp97	−2.3	−0.6	Arg52	1.9	1.1
Arg124	−1.1	−0.2	Tyr42	1.4	1.2
Asp71	−0.8	−0.1	Asp48	1.1	0.2
Lys123	−0.6	−0.1			

[a] PBE was solved using the crystal structure (instead of using the QM/MM-optimized structure). The experimentally measured pK_a value employed as a reference is 8.8 for *p*CA [2]. Note that the contributions of residues to ν_{O-D}, $\nu_{C=O}$, and ΔpK_a values in the ground-state PYP (Tables 5–6) might potentially be overestimated due to the absence of the solvent effect in the present QM/MM calculations. In fact, when dielectric constants of 4 for the protein interior and 80 for bulk water are applied, the contributions of residues to ΔpK_a appear to be lower than those calculated using a QM/MM approach.

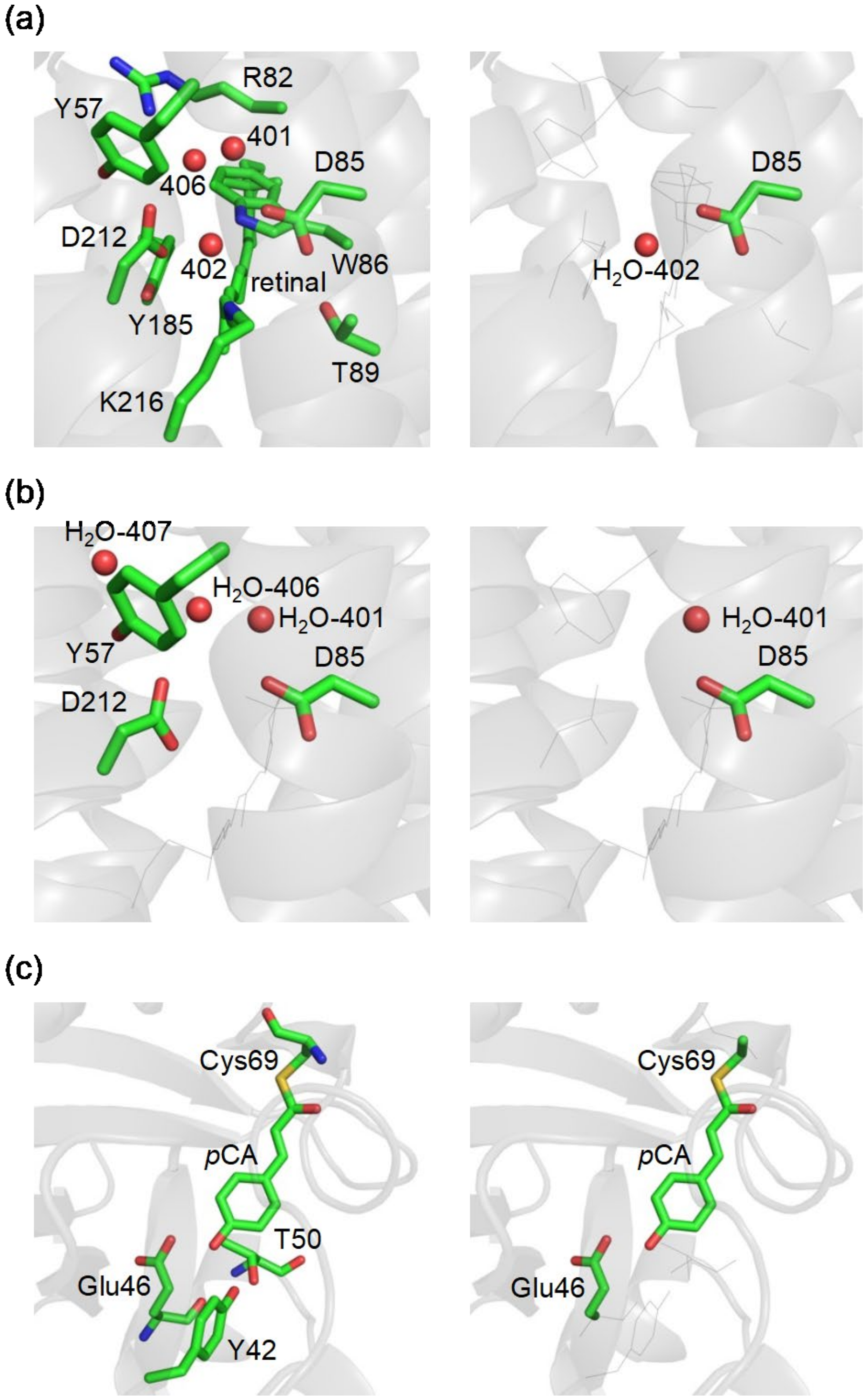

Figure S1. QM region defined in the present study. The initial QM region (left panel) and the redefined QM region (right panel). (a) The ground-state BR (PDB ID: 5ZIM [3]). (b) The N'-state BR (1P8U [4]). (c) The ground-state PYP (1OT9 [5]). Amino acid residues and cofactors in the QM region are shown as green sticks. Water oxygen atoms in the QM region are shown as red spheres.

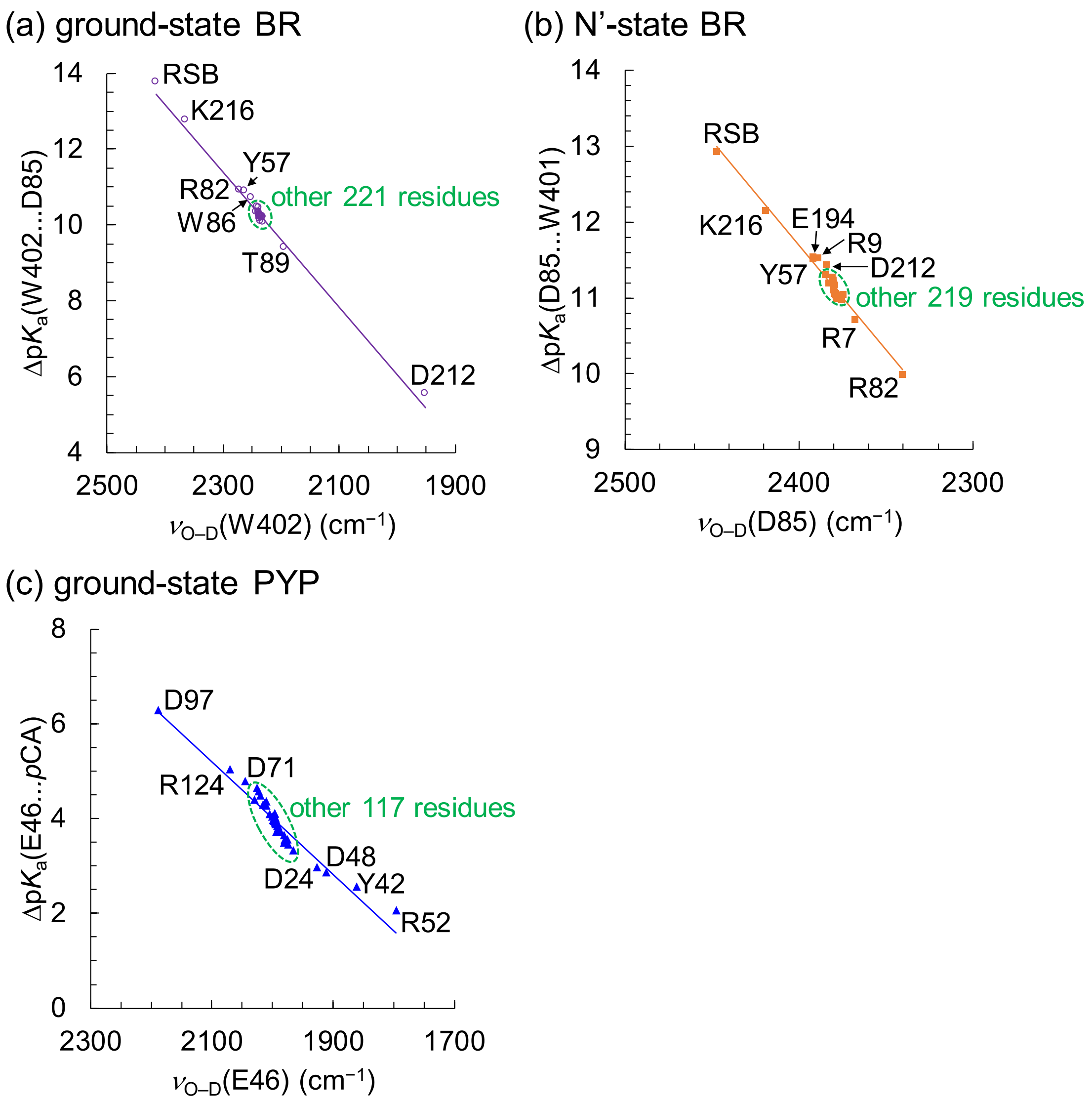

Figure S2. $\nu_{O–D}$(donor) and ΔpK_a calculated in the absence of the electrostatic influence of each residue. (a) Ground-state BR. RSB denotes retinal Schiff base. (b) N'-state BR. (c) Ground-state PYP.

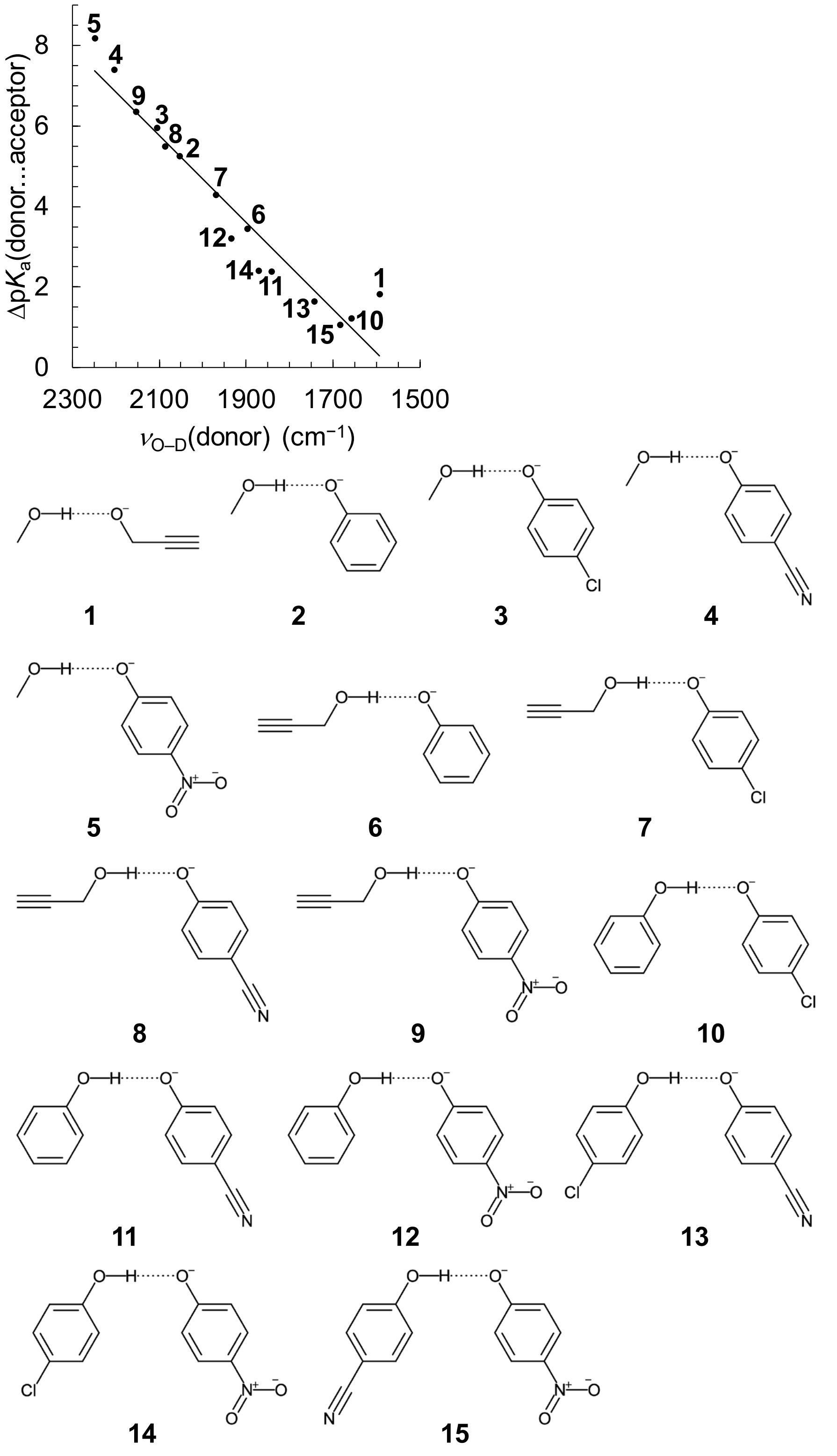

Figure S3. $\nu_{O\text{–}D}$(donor) and ΔpK_a of isolated 15 H-bond pairs. Correlation between $\nu_{O\text{–}D}$(donor) and ΔpK_a (upper panel). Structures of the H-bond pairs (lower panel).

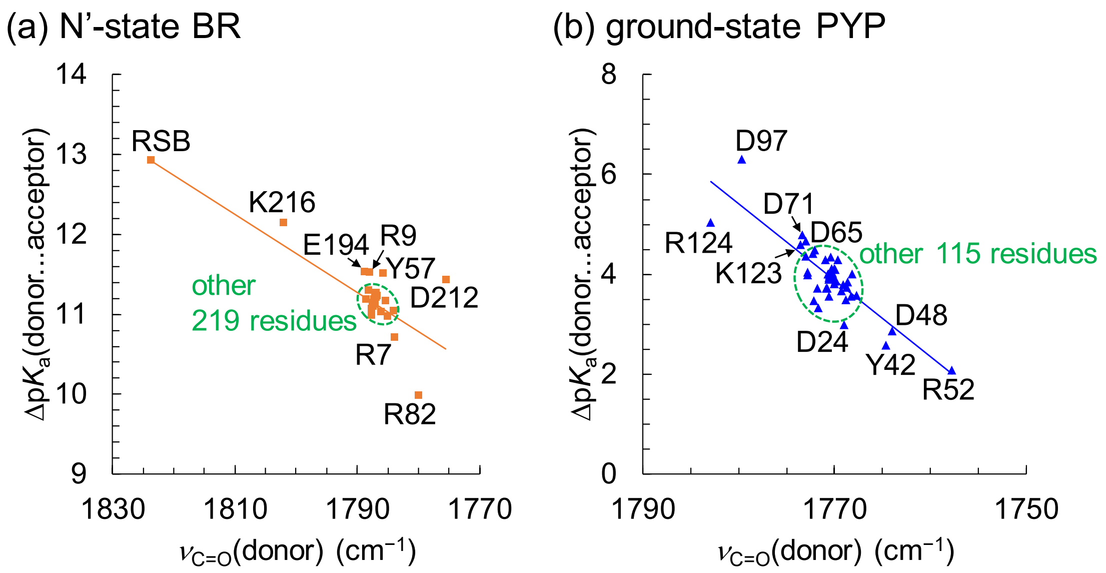

Figure S4. $\nu_{C=O}$(donor) and ΔpK_a calculated in the absence of the electrostatic influence of each residue. (a) N'-state BR. (b) Ground-state PYP.

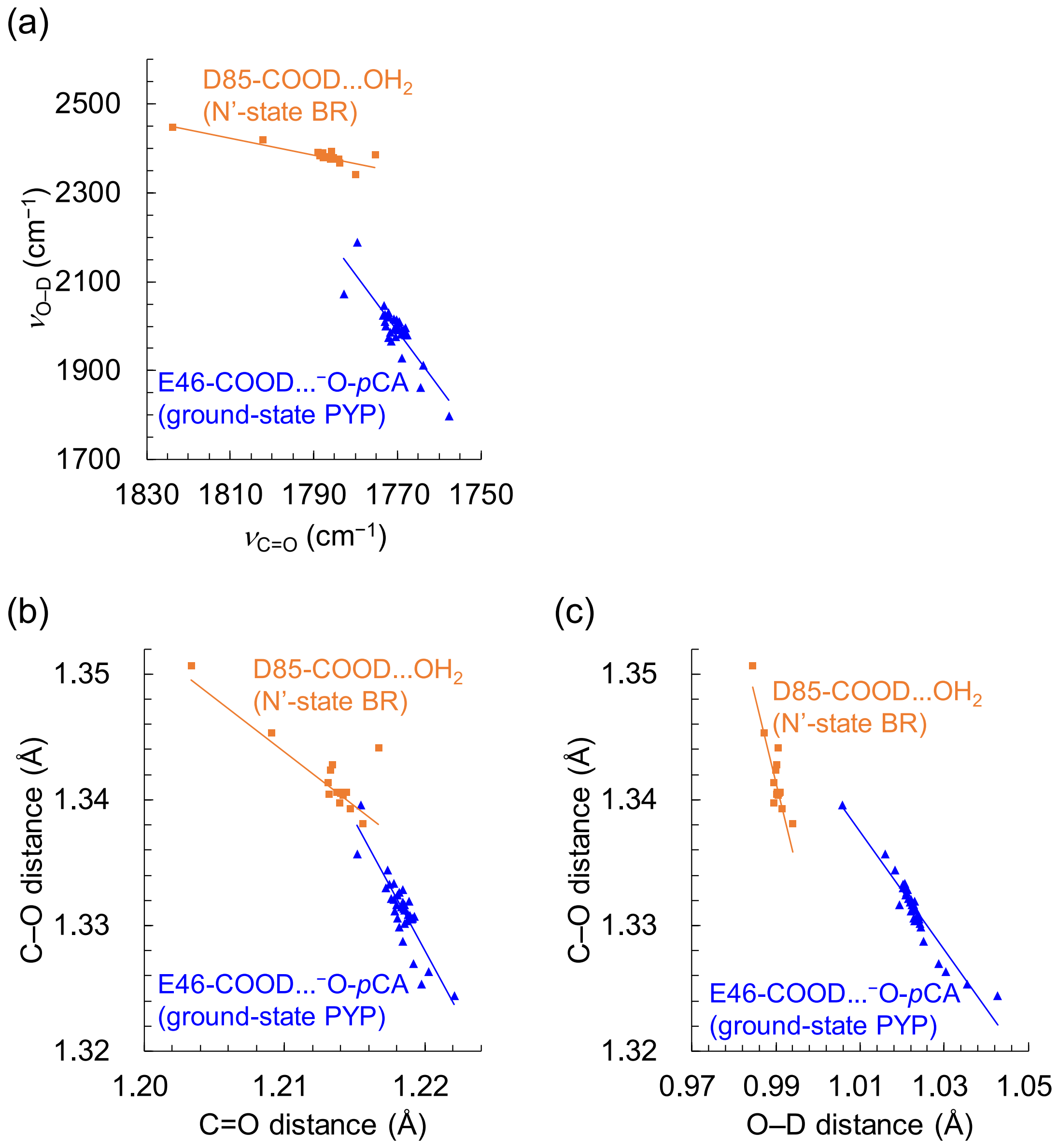

Figure S5. Relationship between $\nu_{C=O}$ and $\nu_{O–D}$ of protonated carboxylate. (a) $\nu_{C=O}$ and $\nu_{O–D}$: the N'-state BR (orange squares); the ground-state PYP (blue triangles). (b) The C=O distance and the C–O distance. (c) The C–O distance and the O–D distance.

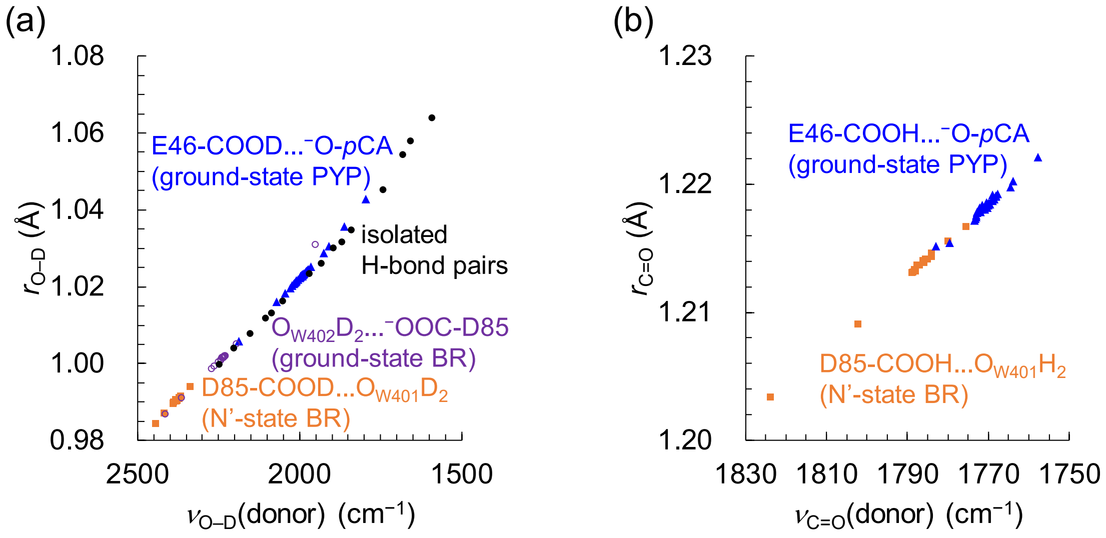

Figure S6. Stretching vibrational frequencies and bond distances obtained from the contribution of each residue. (a) $\nu_{O–D}$(donor) and the O–D bond distance ($r_{O–D}$) in the ground-state BR (purple open circles), the N'-state BR (orange squares), the ground-state PYP (blue triangles), and isolated 15 H-bond pairs listed in Table 1 (black closed circles). (b) $\nu_{C=O}$(donor) and the C=O bond distance ($r_{C=O}$) in the N'-state BR (orange squares) and the ground-state PYP (blue triangles).

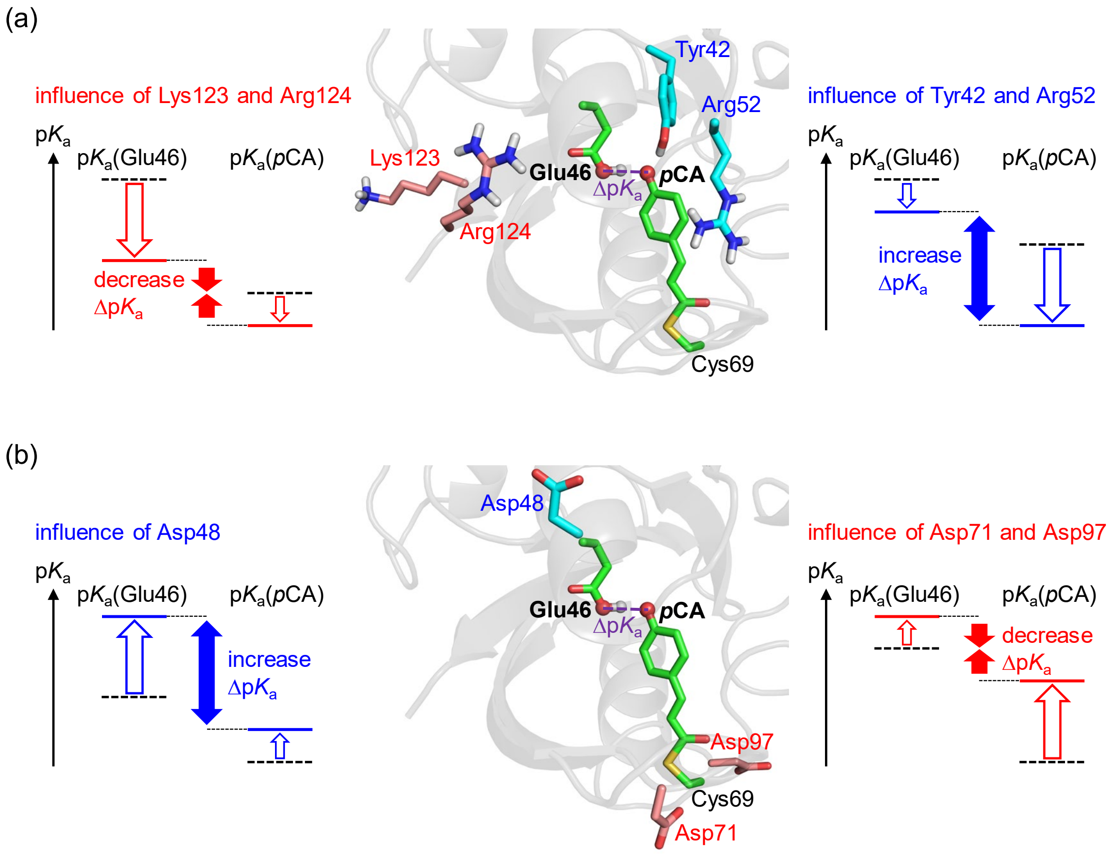

Figure S7. Key residues that influence ΔpK_a(Glu46...*p*CA), $\nu_{O\text{–}D}$(Glu46), and $\nu_{C=O}$(Glu46) in the ground-state PYP. (a) Influence of Tyr42 and basic residues. (b) Influence of acidic residues. Residues that decrease and increase ΔpK_a(Glu46...*p*CA)/ $\nu_{O\text{–}D}$(Glu46)/ $\nu_{C=O}$(Glu46) are red and blue labeled, respectively.

REFERENCES

1 Sagnella, D. E. & Voth, G. A. Structure and dynamics of hydronium in the ion channel gramicidin A. *Biophys. J.* **70**, 2043-2051 (1996).

2 Kroon, A. R. *et al.* Spectral tuning, fluorescence, and photoactivity in hybrids of photoactive yellow protein, reconstituted with native or modified chromophores. *J. Biol. Chem.* **271**, 31949-31956 (1996).

3 Hasegawa, N., Jonotsuka, H., Miki, K. & Takeda, K. X-ray structure analysis of bacteriorhodopsin at 1.3 Å resolution. *Sci. Rep.* **8**, 13123 (2018).

4 Schobert, B., Brown, L. S. & Lanyi, J. K. Crystallographic structures of the M and N intermediates of bacteriorhodopsin: assembly of a hydrogen-bonded chain of water molecules between Asp-96 and the retinal Schiff base. *J. Mol. Biol.* **330**, 553-570 (2003).

5 Anderson, S., Crosson, S. & Moffat, K. Short hydrogen bonds in photoactive yellow protein. *Acta Crystallogr. D Biol. Crystallogr.* **60**, 1008-1016 (2004).
